# Functional Measures in Non-COPD Chronic Respiratory Diseases: A Systematic Review

**DOI:** 10.3390/jcm13226887

**Published:** 2024-11-15

**Authors:** Camile Ludovico Zamboti, Heloise Angélico Pimpão, Larissa Dragonetti Bertin, Gabriela Garcia Krinski, Tathielle Garcia, Sandro Laerth Souza dos Santos Filho, Vinicius Cavalheri, Fabio Pitta, Carlos Augusto Camillo

**Affiliations:** 1 Laboratory of Research in Respiratory Physiotherapy (LFIP), Department of Physiotherapy, Londrina State University (UEL), Londrina 86038-3500, Brazil; 2 Department of Physiotherapy, Faculty of Science and Technology, São Paulo State University (UNESP), Presidente Prudente 19060-900, Brazil; 3 Research Center in Health Sciences, University Pitágoras UNOPAR, Londrina 86038-3500, Brazil; 4 Curtin School of Allied Health, Faculty of Health Sciences, Curtin University, Perth, WA 6102, Australia; 5 Allied Health, South Metropolitan Health Service, Perth 6150, Australia; 6 Exercise Medicine Research Institute, Edith Cowan University, Perth 6027, Australia

**Keywords:** functional status, lung disease, physical functional performance, reproducibility of results, systematic review

## Abstract

**Background/Objectives:** The extensive range of instruments designed for evaluating functional performance (FP) in chronic respiratory diseases (CRD) other than chronic obstructive pulmonary disease (COPD) presents a challenge in selecting the most appropriate one. Therefore, this systematic review aimed to summarise FP instruments, their measurement properties, their minimum clinically important differences, and their associations with CRD course-related events or prognosis in non-COPD CRD. **Methods:** Studies employing patient-reported or performance-based instruments to assess FP in non-COPD CRD were systematically identified in the PubMed, PEDro, Embase, and Cochrane Library databases. COPD-exclusive studies or those solely reporting exercise capacity tests were excluded. Examination focused on measurement properties and associations with CRD course-related events or prognosis. The risk of bias was evaluated using the COSMIN, Downs and Black, and PEDro checklists based on the study design. **Results:** A total of 216 studies across seven CRD categories [asthma, bronchiectasis, cystic fibrosis, interstitial lung disease (ILD), pulmonary arterial hypertension (PAH), pre-/post-lung-transplantation] from various study types were included. Thirty-three instruments were identified, with the SF-36 questionnaire’s physical function domain being the most commonly used patient-reported tool. The 1 min sit-to-stand test was the most extensively studied performance-based measure, with its measurement properties frequently reported in non-COPD CRD studies. Associations with events were infrequently documented, primarily in ILD and PAH studies related to mortality. **Conclusions:** Despite the prevalent use of FP instruments, limited information exists concerning their measurement properties and clinical implications. This review furnishes a concise summary of available evidence, aiding informed clinical decisions when selecting FP tools for non-COPD CRD.

## 1. Introduction

Chronic respiratory diseases (CRDs) encompass various diseases characterised by chronic inflammation and/or infection that compromise the airways and other structures of the lung [1]. The epidemiological causes among CRDs vary, with most being related to repeated exposure to noxious environmental stimuli or severe pulmonary infections or disorders diagnosed with immunological and genetic factors [2]. Each CRD is characterised by a specific disease course, varying the speed of the decline in pulmonary function and number and frequency of pulmonary exacerbation episodes, impacting the rate of the disease progression and risk for hospitalisation and mortality [3]. Some of the most common CRDs includes chronic obstructive pulmonary disease (COPD), asthma, bronchiectasis, and as interstitial lung disease (ILD) [4]. COPD is the most studied CRD [2], although other CRDs, such as asthma, bronchiectasis, and ILD, are also prevalent and contribute to the leading global causes of death [4].

Besides the differences in disease progression, individuals with CRDs often experience similarities in the symptoms or extrapulmonary manifestations, such as peripheral muscle weakness, progressive breathlessness, and reduced exercise tolerance, leading to decreased engagement in daily physical activity, thus affecting their functional performance [5]. Therefore, evaluating patients’ ability to carry out everyday tasks has become crucial in managing CRDs [6]. Functional performance (FP) constitutes activities necessary to meet basic needs and is part of functional status, encompassing tasks of various demands, from basic movements to high-effort activities [6,7].

Assessment of FP can be achieved using patient-reported tools, such as questionnaires or scales gauging FP domains, or performance-based tests measuring FP [6]. Patient-reported tools consider individuals’ perspectives on their capabilities and obstacles in engaging in activities. FP tests evaluate everyday tasks involving physical performance and body position changes [6,8]. While maximal effort tests such as cardiopulmonary exercise tests (CPETs) or the six-minute walk test (6MWT) provide significant physiological and prognostic information, FP tests offer practicality in space and time requirements and better simulate daily life activities [6,8].

Investigations into FP are prevalent in CRDs, particularly in COPD [7,9]. Several studies have explored FP in COPD, with recent systematic reviews summarizing FP tools, their measurement properties, and their correlation with mortality and hospitalisation [7,8,9,10,11,12]. Although FP is commonly assessed in patients with other CRDs [13,14,15] and is used to measure the efficacy of pulmonary rehabilitation [16] or its relationship with disease prognosis [17], a similar comprehensive summary is lacking for FP tools in non-COPD CRDs. Since the aetiology, treatment, prognosis, and patient characteristics in each CRD differ, FP tools cannot be used interchangeably across CRDs without Supporting Data proving their applicability and validity.

In this context, selecting the most suitable FP instrument for a specific CRD poses challenges due to the abundance of available tools, inconsistent measurement properties, and conflicting information [10]. Therefore, a systematic review investigating FP and providing a summary of their measurement properties will facilitate the selection of the most adequate tool for each non-COPD CRD in clinical practice. This systematic literature review aimed to identify, describe, and discuss FP instruments, both performance-based and patient-reported, used in non-COPD CRDs, as well as to report measurement properties and associations of instruments with events related to the course of CRD or prognosis in non-COPD CRD.

## 2. Materials and Methods

### 2.1. Protocol, Database Search, and Selection Criteria

The protocol for this systematic review was registered in PROSPERO (CRD42018102771). Four electronic databases were searched: the PubMed, PEDro, EMBASE, and Cochrane Library databases. Any article published until the final search conducted in May 2023 was included. The search included only terms that were related to or described functional tests in CRDs. The search terms used were a combination of: (lung or pulmonary or respiratory or cystic fibrosis) AND (walk/gait speed or sit-to-stand or chair-stand or 4MGS or muscle dysfunction or physical function or physical performance or functional capacity or physical fitness), adapted according to each database and presented in Table S1 (Supplementary Data). Inclusion criteria considered full manuscripts published in any language assessing FP in people with any non-COPD CRDs using patient-reported tools or performance-based tests.

In case of patient-reported tools that included domains to assess different outcomes, only those domains associated with FP were considered. Only instruments investigating activities motivated by personal bodily needs as activities of daily living (ADL), such as intermediate activities to enable these needs [6], were included. Performance-based tests were deemed FP if the effort done was needed to complete basic tasks or if the body position changed to perform shorter activities of daily living, such as sitting or rising from a chair [6]. The list of performance-based tests and patient-reported tools obtained in previous systematic reviews with COPD patients were used as a guide to select the instruments included [9,11]. Studies were excluded if they included tests requiring maximal effort to complete (i.e., 6MWT or CPET), or if they included tests not specifically measuring the ability to fulfil basic needs (i.e., movements and changes in body positions) [9]. Furthermore, questionnaires that did not provide specific measurements related to the FP domain were excluded. Studies with mixed results including people with COPD, lung cancer, and other CRDs, or studies with groups of children in which analysis by subgroups was not undertaken/reported, were excluded. Case reports, case series, and studies with only qualitative results were excluded.

Search yields were exported to the Mendeley Desktop 1.19.3© software (Elsevier, NY, USA), and duplicates were removed. Studies were screened via title and abstract by two independent reviewers (CZ and TG), and disagreements were resolved by an independent reviewer (CAC). The full text of all relevant studies was obtained and read to ensure the inclusion criteria were met. If there was insufficient information to include/exclude an article, the authors were contacted when possible.

### 2.2. Data Collection and Quality Appraisal

Data were extracted into an electronic spreadsheet. Type of CRD, characteristics of included patients, and information on FP instruments were collected. Metric and psychometric properties were collected for objective (i.e., tests) and subjective (i.e., questionnaires) instruments, respectively, including data on validity, reliability, and minimal clinically important difference (MCID). Correlation data in studies included without the intention to investigate validity were not considered as measurement properties. Furthermore, any information regarding association of performance with events related to the course of CRD or prognosis, such as mortality, hospitalisations, and exacerbation frequency, was extracted from the studies.

Risk of bias or quality was assessed by one reviewer (CZ) using different instruments according to the design of the included studies: (i) the COSMIN Risk of Bias Checklist for studies with assessment of metric or psychometric properties [18]; (ii) the PEDro scale for randomised clinical trials [19]; and (iii) the Downs and Black checklist for all other study types [20]. In the studies reviewed, only correlations related to validity, specifically concurrent or criterion validity, were reported. The criterion for determining the validity of the instrument was based on correlation values, with a threshold of at least 0.5. Correlations reported were deemed negligible to weak (r < 0.4), moderate (0.4 ≤ r < 0.69), or strong (0.7 ≤ r ≤ 1.0) [21]. Reliability was deemed moderate if intraclass correlation coefficient (ICC) values were between 0.50 and 0.75, good if between 0.76 and 0.90, and excellent if above 0.90 [22]. Additionally, the MCID of FP instruments, whenever available, was reported.

## 3. Results

**Figure 1:**
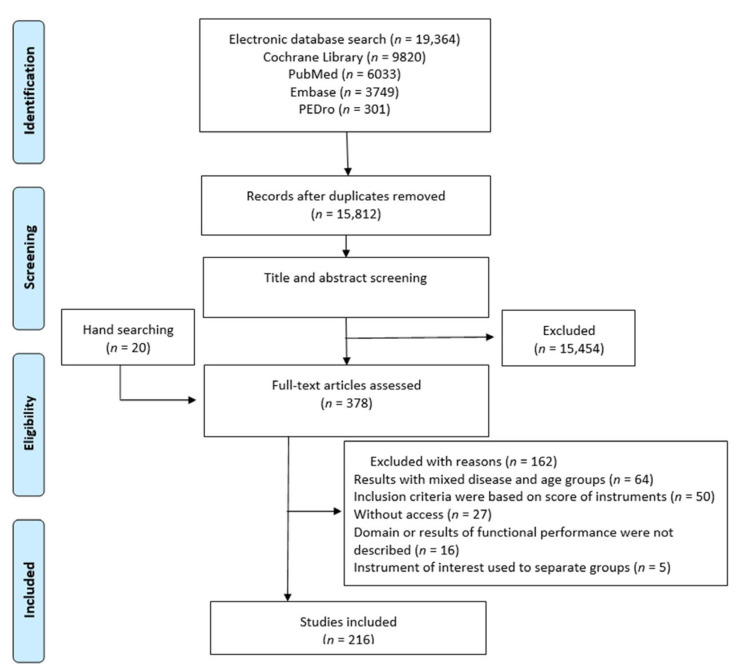
Study Flowchart.

From the 15.812 records identified in the database search, 216 studies [13,14,15,17,23,24,25,26,27,28,29,30,31,32,33,34,35,36,37,38,39,40,41,42,43,44,45,46,47,48,49,50,51,52,53,54,55,56,57,58,59,60,61,62,63,64,65,66,67,68,69,70,71,72,73,74,75,76,77,78,79,80,81,82,83,84,85,86,87,88,89,90,91,92,93,94,95,96,97,98,99,100,101,102,103,104,105,106,107,108,109,110,111,112,113,114,115,116,117,118,119,120,121,122,123,124,125,126,127,128,129,130,131,132,133,134,135,136,137,138,139,140,141,142,143,144,145,146,147,148,149,150,151,152,153,154,155,156,157,158,159,160,161,162,163,164,165,166,167,168,169,170,171,172,173,174,175,176,177,178,179,180,181,182,183,184,185,186,187,188,189,190,191,192,193,194,195,196,197,198,199,200,201,202,203,204,205,206,207,208,209,210,211,212,213,214,215,216,217,218,219,220,221,222,223,224,225,226,227,228,229,230,231,232] met eligibility criteria and were included in the review (Figure 1). Information on included studies and reasons for exclusion after full text assessment are described in Tables S2 and S3 (see Supplementary Data).

Seven subgroups of CRD were identified, and the subgroup with the most studies was interstitial lung disease (ILD) (*n* = 70 studies), followed by pulmonary arterial hypertension (PAH) (*n* = 67 studies), asthma (n = 48 studies), cystic fibrosis (CF) (*n* = 15 studies), and bronchiectasis (*n* = 14 studies). A low number of studies investigating patients with CRD waiting for (pre-LTx) (*n* = 2 studies) [136,233] and following lung transplantation (post-LTx) (*n* = 2 studies) [64,137] were found (Figure S1, Supplementary Data). A summary of the findings for pre-LTx and post-LTx patients is provided in Supplementary Data. There were no studies investigating FP in patients submitted to pulmonary resection in a specific respiratory disease; only mixed results with lung cancer patients were identified. Therefore, studies regarding pulmonary resection were excluded. Characteristics of patients per disease are described in Table S4 (Supplementary Data). The designs of the included studies were experimental studies (*n* = 93 studies), cross-sectional studies (*n* = 92 studies), and longitudinal cohorts (*n* = 31 studies).

**Figure 2:**
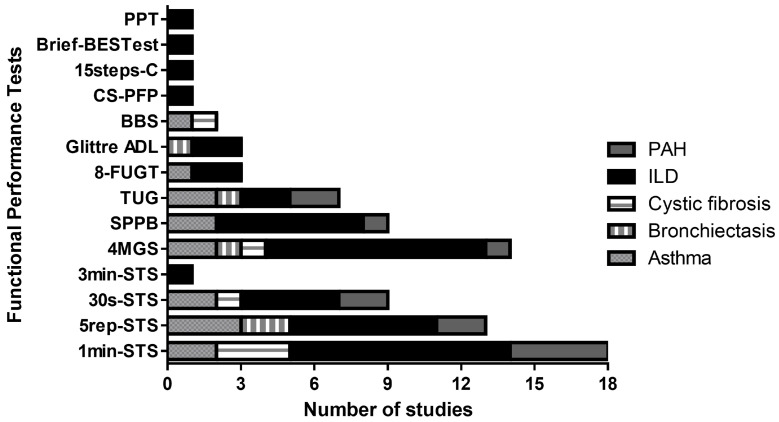
Studies using functional performance tests by chronic respiratory disease. Abbreviations: ILD–interstitial lung disease; PAH—pulmonary arterial hypertension; 15steps-C—15-step climbing; CS-PFP—continuous scale physical function performance test; Glittre ADL—Glittre Activities of Daily Living; 8-FUGT—8-foot up-and-go; TUG—timed up-and-go; SPPB—short physical performance battery; 4MGS—four-metre gait speed; 3min-STS—3 min sit-to-stand; 5rep-STS—5-repetition sit-to-stand; 30 s-STS—30 sec sit-to-stand; 1min-STS—1 min sit-to-stand; PPT—physical performance test.

In total, 33 instruments were identified (12 performance-based and 21 patient-reported tools). The most investigated performance-based test in non-COPD CRD patients was the one-minute sit-to-stand (1 min-STS) (*n* = 18 studies), followed by four-metre gait speed (4MGS) (*n* = 14 studies) and five-repetition sit-to-stand (5rep-STS) (*n* = 13 studies). All tests focused on the assessment of lower limb functional limitations. The full overview of performance-based tests according to disease is presented in Table 1, and a summary is provided in Figure 2.

**Figure 3:**
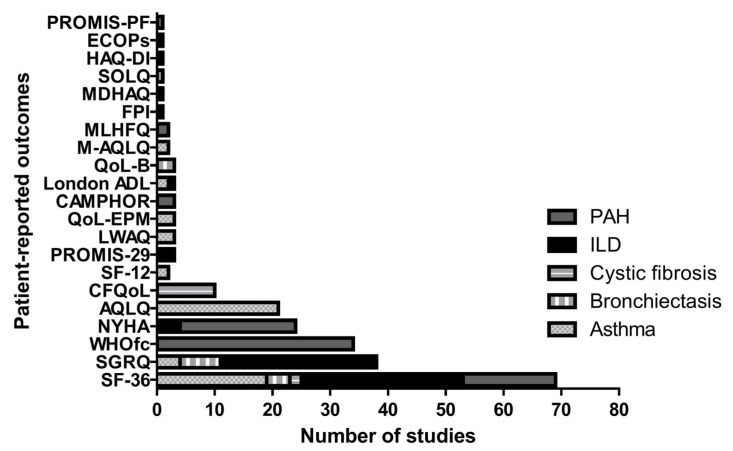
Frequency of patient-reported tools per disease. Abbreviations: ILD—interstitial lung disease; PAH—pulmonary arterial hypertension; pre-LTx—patients with chronic respiratory disease awaiting lung transplantation; post-LTx—patients with chronic respiratory disease who underwent lung transplantation; SF-36—Medical Outcomes Study 36-Item Short Form of Health Survey; SGRQ—Saint’s George Respiratory Questionnaire; WHOfc—World Health Organisation functional class; NYHA—New York Heart Association; AQLQ—Asthma Quality of Life Questionnaire; CFQoL—Cystic Fibrosis Quality of Life; SF-12—Medical Outcomes Study 12-Item Short Form of Health Survey; PROMIS-29—Patient-Reported Outcomes Measurement Information System; LWAQ—Living with Asthma Questionnaire; M-AQLQ—Mini Asthma Quality of Life Questionnaire; CAMPHOR—Cambridge Pulmonary Hypertension Outcome Review; QoL-EPM—Asthma Quality of Life from Escola Paulista de Medicina; MLHFQ—Minnesota Living with Heart Failure Questionnaire; QoL-B—Quality of Life in Bronchiectasis; London ADL—London Activities of Daily Living; FPI—functional performance inventory; SOLQ—Seattle Obstructive Lung Questionnaire; ECOPs—Eastern Cooperative Oncology Performance Status; PROMIS-PF—Patient-Reported Outcomes Measurement Information System Physical Function Short Form 8a; MDHAQ—Multidimensional Health Assessment Questionnaire; HAQ-DI—Health Assessment Questionnaire Disability Index.

The most common patient-reported tools to assess the domain of FP were the physical function domain (PFd) and physical component score (PCS) of the Medical Outcomes Study 36-item Short Form of Health Survey (SF-36) (*n* = 72 studies), followed by the activities domain of Saint George’s Respiratory Questionnaire (SGRQ) (*n* = 39 studies) and the World Health Organisation functional class (WHOfc) (*n* = 34 studies). The full overview of the patient-reported instruments according to disease is depicted in Table 2, and a summary is provided in Figure 3.

A description of the risk-of-bias or quality assessment of the included studies is provided in Tables S5–S7 (see Supplementary Data). The quality of the included studies varied from poor or inadequate (*n* = 73 studies) to very good or excellent (*n* = 33 studies). Thirty studies reported measurement properties. Table 1 and 2 describe values obtained in the included studies reporting the validity, reliability, and interpretability of the FP instruments included. All studies with metric or psychometric properties were evaluated by the COSMIN risk of bias, most with very good methodological quality (*n* = 29 studies). Validity and reliability were the most reported metric or psychometric properties, and ILD and asthma were the CRDs with the largest number of studies investigating these properties.

Few studies in asthma, bronchiectasis, CF, ILD, and PAH reported their instruments’ association with events related to the course of disease. The associations found were demonstrated with disease severity, frequency of exacerbations, hospitalisation, and mortality (Table 1 and Table 2). A summary of the measurement properties, MCIDs, and associations with events related to the course of CRD found in the included studies is provided in Table 3. A further detailed description of the performance-based and patient-report tools, as well as their properties and clinical implications, can be found in Supplementary Data and Table S8.

## 4. Discussion

### Implications for Practice: Selection of Instruments by Non-COPD CRD

The results of this systematic review demonstrate the large number of available tools to assess FP in non-COPD CRD patients. Unfortunately, few tools have had their metric or psychometric properties thoroughly investigated. Evidence for the association of FP with events related to the course of CRD or prognosis is scarce, with mortality reported only in people with ILD and PAH. For instance, there is no tool with all “boxes ticked” for each non-COPD CRD patient that assesses FP, has had a comprehensive set of metric or psychometric properties analysed, and has had its association with events related to the course of CRD or prognosis investigated.

The selection of the most appropriate tool to assess FP, thus, should be based on one’s needs. In this context, since there is no single test that is valid, reliable, has MCID reported, and is related to the course of disease, physical therapists treating CRD patients should consider all valid and reliable tools for the specific CRD and select one of them to assess their patients according to the most important functional component of the test. Among the valid and reliable tools, if the intention is to investigate responsiveness to an intervention, then FP tools with reported MCIDs are those of preference. Instead, if one’s need is to identify prognosis or risk of hospitalisations, those with such information should be used. A summary for selecting the most appropriated tool for each disease, according to the current literature, is detailed below and provided in Table 3.

In asthma patients, three performance-based tests achieved validity and reliability: 5rep-STS, short physical performance battery, and timed up-and-go (TUG), with all of them classified as very good methodological quality [13] in one study with 52 middle-aged patients [47 (38—58) years], mostly women with severe or very severe asthma (i.e., 76% with GINA 4 or 5) [13]. Furthermore, the results of these three tests were significantly worse in asthmatic patients than in healthy controls (*p* < 0.05 for all). All three tests can be used for asthma patients; however, the MCID and the association with events related to the course of CRD or prognosis remain to be elucidated for all of them.

The activity domain of the Asthma Quality of Life Questionnaire (AQLQ) was the only patient-reported instrument in the literature that was valid and reliable and had an MCID demonstrated in very-good-methodological-quality studies [211,212] including 39 patients, mostly middle-aged women (42 ± 13 years) with mild reductions in spirometry (85 ± 14% of predicted of forced expiratory volume in one second, FEV_1_). Although the score on the AQLQ was worse in patients with nonstable compared with stable asthma (*p* < 0.001) [212], the association with events related to the course of CRD or prognosis was not described for the activity domain in AQLQ. It is worth mentioning that one study, which was excluded from this review, demonstrated the validity and reliability of the SGRQ in asthma patients, including children [234].

In bronchiectasis, there was no performance-based test with investigated metric properties, MCID, or association with events related to the course of CRD or prognosis in the included studies. Studies in bronchiectasis used 5rep-STS, 4MGS, TUG, and Glittre ADL. It is, therefore, not possible to provide clear guidance on the selection of a performance-based tool in bronchiectasis, and studies investigating these properties in patients with bronchiectasis are needed, especially for these instruments. The physical functional score of the Seattle Obstructive Lung Questionnaire (SOLQ) was the only patient-reported instrument that was validated, reliable, and associated with exacerbation frequency. This information was derived from one study (78 patients, 58% women, 48 ± 13 years, FEV_1_% 78 ± 18) with doubtful methodological quality [90].

In CF patients, the 1min-STS was the only test that was valid and reliable and had an MCID reported in two studies with very good methodological quality [14]. Notably, these two studies were conducted with small sample sizes (i.e., ≤15 patients), 52% men, with a very limited age range (29–31 years old) and FEV_1_% varying from 34 to 56%. Therefore, is possible to recommend the 1min-STS to assess FP in CF in young adults, although its association with events related to the course of CRD or prognosis remains to be explored. The Cystic Fibrosis Quality of Life Questionnaire was the unique patient-reported instrument that was valid and reliable, as derived from a very-good-methodological-quality study [32 patients, 53% men, median age of 27 (16–53) years] [94], but without MCID or association with events related to the curse of CRD or prognosis.

In ILD patients, many performance-based tests were valid and reliable (i.e., 1 min-STS, 5rep-STS, 3 min sit-to-stand (3 min-STS), 4MGS, TUG, Glittre ADL, and continuous scale physical function performance) in nine studies [15,29,30,43,58,60,67,68,69], most of which had very good methodological quality. There was no MCID reported for any of the above tests, and future studies investigating the MCID and responsiveness are needed. With the exception of the 3 min-STS, all the aforementioned instruments had validity against other functional capacity tests (i.e., 6MWT). However, strong correlations were found only with the 1min-STS [30], 4MGS [60], and Glittre ADL [68]. Furthermore, excellent reliability in inter- and intrarater analysis was found only for the 4MGS [43]. In this context, except for the lack of MCID, the 4MGS was the most adequate performance-based test for ILD patients (valid, reliable, and with associations with hospitalisation and mortality) [17,43,57,60], as mostly derived from very-good-methodological-quality studies and including only idiopathic pulmonary fibrosis (IPF) patients. Many studies (i.e., 25 of 70 studies) in the ILD group were conducted in IPF-only cohorts. In studies specifically focusing on IPF, details regarding validation, reliability, and correlation with adverse outcomes were provided for the 8-foot up-and-go test (8-FUGT) [50], 3min-STS [29], SF-36 [102], and SGRQ [98]. A summary of instruments’ findings in IPF patients is provided in the Supplementary Data (Tables S9 and S10).

Although SGRQ-I [98] and the Patient-Reported Outcomes Measurement Information System (PROMIS-29) [106,109] appear to have acceptable psychometric properties, the PFd of SF-36 was the only patient-reported tool with psychometric properties and MCID demonstrated in a study (258 patients, 73% men, 67 ± 10 years, forced vital capacity of 62 ± 19% of predicted) with doubtful to very good methodological quality [102]. In this context, if the MCID is one of main aspects for selecting a tool, the PFd of SF-36 may be more useful than SGRQ-I and PROMIS-29. There were no associations between patient-reported tools and events related to the course of CRD or prognosis. Recommendations for patient-centred outcome selection including different outcomes in ILD exist [235], including questionnaires and scales assessing different outcomes (e.g., respiratory symptoms). Nevertheless, the review did not offer explicit recommendations for the selection of FP instruments, nor did it furnish information concerning the instruments’ measurement properties.

In PAH patients, the 30 s sit-to-stand test (30 s-STS) and TUG were valid and reliable in intrarater analysis [54] but lacked information on MCID or association with events related to the course of CRD or prognosis. Both tests were assessed in one study (39 patients, 81% women, 50 ± 18 years) with doubtful to very good methodology quality [54]. Given the patient’s activity goal, one of these two instruments may be more suitable. The PSS of the MLHFQ was valid and reliable and showed association with worse prognosis (i.e., mortality, lung transplantation, and pulmonary endarterectomy) [193] in one study [48 patients, 65% women, 50 (46–54) years] with inadequate to very good methodological quality. Notably, in patients with PAH, the WHOfc was the instrument most used (i.e., in 34 studies) and demonstrated an association with mortality [171]. The search results, however, did not yield psychometric properties for the WHOfc, indicating a need for assessment in future studies. Finally, a brief description of the included studies investigating pre-LTx and post-LTx is depicted in the Supplemental Material, contrasted with relevant information in the current literature [236].

A strength of this study is the ease for clinicians to verify whether a specific tool suffices one’s needs. This is, however, the first review that provided a compilation of both performance-based and patient-reported tools to assess FP in non-COPD CRD patients. The provision of details regarding construct and measurement properties and clinical implications of the tools (i.e., negative impact) offers a guide to tool selection. Though there is a vast array of instruments used to assess FP in non-COPD CRD, the large heterogeneity of the studies and the limited information on their measurement properties are of concern. In addition, because of the large number of included studies, the risk-of-bias assessment was performed by a single author. Valid tools described in the literature were selected for this purpose, and the guidelines of each tool were carefully followed to assess the risk of bias in the included studies.

In COPD, there are at least four systematic reviews [8,9,11,12] for both performance-based and patient-reported instruments. Therefore, upcoming studies should ideally emulate the trajectory already established in the COPD literature. For instance, at least five performance-based tests included in this systematic review (TUG, 5rep-STS, 1min-STS, 3min-STS, and 4MGS) have validity, reliability, responsiveness, MCID, and associations with different adverse clinical consequences in COPD patients. Finally, the abundance of available FP instruments does not inherently guarantee a broader selection of reliable tests. A recent study in COPD [237] highlighted the diversity in outcomes and measurements documented, making comparability among trials in pulmonary rehabilitation difficult. Future studies should focus on indicating specific domains or instruments of functional performance that researchers should consider for improving the consistency and comparability of studies.

## 5. Conclusions

The findings of the present systematic review showed that FP has been vastly studied in non-COPD CRD patients. Compared with performance-based tests, patient-reported tools (i.e., questionnaires and scales) were more frequently used. Although FP instruments are widely used in non-COPD CRD patients, currently only a few studies have reported measurement properties and associations with clinical implications. Future studies focusing on measurement properties and investigating associations with events related to the course of CRD or prognosis are necessary.

## Figures and Tables

**Table 1:** Metric properties and associations with negative outcomes of performance-based tests in non-COPD chronic respiratory diseases.

Performance-Based Tests	Validity	Reliability	Interpretability	Associations with Events Related to the Course of Crd or Prognosis
1 min-STS	**Asthma**: 6MWD (r = 0.48; *p* < 0.0001); HGS (r = 0.41; *p* = 0.003); QS MIVC (r = 0.32; *p* = 0.028) [13] *(Very Good)* **CF**: Watts_máx_ CPET (r = 0.93; *p* < 0.05); VO_2_ peak (0.62 < r < 0.91; *p* < 0.05) [14] *(Very Good)* **ILD**: 6MWD (0.33 < r < 0.82; *p* < 0.05); HGS (r = 0.35; *p* < 0.05); QS MIVC (r = 0.34; *p* < 0.05); FVC%pred (r = 0.48; *p* < 0.05); TCLO%pred (r = 0.47; *p* < 0.001) [15] *(Very Good)* **PAH**: 6MWD (r = 0.71; *p* < 0.001) [34] *(Very Good)*	**Asthma**: Intrarater [ICC:0.87 (95% CI: 0.73–0.93)] and inter-rater [ICC: 0.80 (95% CI: 0.62–0.89)] [13] *(Very Good)* **CF**: Intrarater [ICC:0.98 (95% CI: 0.96–0.99)] [14] *(Adequate)* **ILD**: Intrarater [0.88 < ICC < 0.92 (95% CI: 0.82–0.95)] [15,29] and inter-rater [ICC: 0.91 (95% CI: 0.85–0.95)] [15] *(Very Good)*	**CF** MCID of 5.4 rep [14] *(Adequate)*	
5rep-STS	**Asthma**: 6MWD (r = −0.56; *p* < 0.0001); HGS (r = 0.49; *p* < 0.0001); QS MIVC (r = −0.34; *p* = 0.018) [13] *(Very Good)* **ILD**: 6MWD (−0.26 > r > −0.41; *p* > 0.05); HGS (r = −0.38; *p* < 0.05); QS MIVC (r = −0.50; *p* < 0.05); mMRC (r = 0.18; *p* > 0.05) [15,43] *(Very Good)*	**Asthma**: Intrarater [ICC:0.84 (95% CI: 0.72–0.90)] and inter-rater [ICC: 0.86 (95% CI: 0.75–0.92)] [13] *(Very Good)* **ILD**: Intrarater (0.85 < ICC < 0.87; 95% CI 0.66–0.94) and inter-rater [ICC: 0.90 (95% CI: 0.82–0.94)] [15,43] *(Doubtful to Very Good)*		
30 s-STS	**Asthma**: 6MWD (r = 0.45; *p* < 0.0001); QS MIVC (r = 0.43; *p* = 0.002); QS MIVC (r = 0.34; *p* = 0.02) [13] *(Very Good)* **CF**: QS peak torque (r = 0.55; *p* = 0.034) [48] *(Very Good)* **ILD**: 6MWD (r = 0.28; *p* > 0.05); HGS (r = 0.41; *p* < 0.05); QS MIVC (r = −0.41; *p* < 0.05) [15] *(Very Good)* **PAH**: QS MIVC (r = 0.54; *p* < 0.001); 6MWD (r = 0.66; *p* < 0.001) [54] *(Very Good)*	**Asthma**: Intrarater [ICC:0.91 (95% CI: 0.81–0.95)] and inter-rater [ICC: 0.86 (95% CI: 0.73–0.93)] [13] *(Very Good)* **ILD:** Intrarater [ICC: 0.90; (95% CI: 0.89–0.95)] and inter-rater [ICC: 0.85 (95% CI: 0.73–0.91)] [15] *(Very Good)* **PAH**: Intrarater [ICC:0.95 (95% CI: 0.90–0.97)] [54] *(Doubtful)*		
3 min-STS	**ILD:** FVC%pred (r = 0.43; *p* < 0.05); TCLO%pred (r = 0.55; *p* < 0.001) [29] *(Very Good)*	**ILD:** Test–retest [ICC:0.96 (95% CI: 0.92–0.98)] [29] *(Very Good)*		
4MGS	**Asthma**: 6MWD (r = −0.64; *p* < 0.0001); HGS (r = −0.52; *p* < 0.0001); QS (r = −0.30; *p* = 0.040) [13] *(Very Good)* **ILD:** 6MWD (0.55 < r<0.77; *p* > 0.05); KBILD total (r = 0.44; *p* < 0.001); mMRC (−0.56 < r>0.40; *p* > 0.05); GAP index (r = −0.41; *p* = 0.002); HGS (0.37 < r > 0.57; *p* > 0.05) [15,43,58,60] *(Very Good)*	**Asthma**: Intrarater [ICC: 0.86 (95% CI: 0.73–0.92)] and inter-rater [ICC: 0.58 (95% CI: 0.26–0.76)] [13] *(Very Good)* **ILD:** Intrarater (0.92 > ICC > 0.95; 95% CI: 0.73–0.99) and inter-rater (0.56 > ICC > 0.98; 95% CI: 0.32–0.99) [15,43,60] *(Doubtful to Very Good)*		**ILD:** 4MGS < 0.08 m/s was an independent predictor of hospitalisation [HR: 2.63 (1.0–6.8); *p* = 0.04] and all-cause mortality [HR: 2.76 (1.1–6.5); *p* = 0.02] [17] *(Fair)* 4MGS independent predict disease severity (FVC% and DLCO%) (0.07 < r^2^ < 0.16; *p* < 0.05) [59] *(Poor)* Decline in 4MGS ≥ 0.07 m/s is associated with death in 6 months (Kaplan–Meier curves comparing decline ≥ 0.07 m/s versus decline ≤ 0.07 m/s; *p* = 0.007) [57] *(Good)*
SPPB	**Asthma**: 6MWD (r = 0.61; *p* < 0.0001); HGS (r = 0.50; *p* < 0.0001); QS MIVC (r = 0.39; *p* = 0.006) [13] *(Very Good)* **ILD:** 6MWD (r = 0.35; *p* < 0.05) [15] *(Very Good)*	**Asthma**: Intrarater [ICC:0.75 (95% CI: 0.56–0.86)] and inter-rater [ICC: 0.75 (95% CI: 0.56–0.86)] [13] *(Very Good)* **ILD:** Intrarater [ICC:0.83 (95% CI: 0.71–0.91)] and inter-rater [ICC: 0.75 (95% CI: 0.59–0.86)] [15] *(Very Good)*		
TUG	**Asthma**: 6MWD (r = −0.62; *p* < 0.0001); HGS (r = −0.49; *p* < 0.0001); QS MIVC (r = −0.43; *p* = 0.002) [13] *(Very Good)* **ILD:** 6MWD (r = −0.69; *p* < 0.05); QS MIVC (r = −0.48; *p* < 0.05); HGS (r = 0.56; *p* < 0.05) [15] *(Very Good)* **PAH**: QS MIVC (r = −0.38; *p* = 0.017); 6MWD (r = −0.77; *p* < 0.001) [54] *(Very Good)*	**Asthma**: Intrarater [ICC: 0.90 (95% CI: 0.82–0.94)] and inter-rater [ICC: 0.76 (95% CI: 0.56–0.87)] [13] *(Very Good)* **ILD:** Intrarater [ICC:0.88 (95% CI: 0.79–0.93)] and inter-rater [ICC: 0.89 (95% CI: 0.81–0.94)] [15] *(Very Good)* **PAH**: Intrarater [ICC:0.96 (95% CI: 0.93–0.98)] [54] *(Doubtful)*		
8-FUGT				Performance in 8-FUGT > 6.9 s was associated with hospitalisation [HR: 14.1 (3.5–56); *p* < 0.001] and mortality [HR: 55.4 (5–592); *p* = 0.001] [50] *(Fair)*
Glittre ADL	**ILD:** 6MWD (−0.90 < r <−0.70; *p* < 0.005); total EE (r = −0.52; *p* = 0.02) [67] *(Very Good)*	**ILD:** Test–retest (0.90 < ICC < 0.98; 95% CI: 0.74–0.99) [67] *(Very Good)*		
CS-PFP	**ILD:** SGRQ–activities (r = −0.80; *p* = 0.0002); 6MWD (r = 0.66; *p* = 0.008); SF-36–PFd (r = 0.64; *p* = 0.007); D_LCO_ %predicted (r = 0.67; *p* = 0.006); FVC %predicted (r = 0.63; *p* = 0.009) [69] *(Very Good)*	**ILD:** Intrarater (ICC:0.83) [69] *(Adequate)* *^A^*		
15-steps C				
PPT				

Interpretability refers to minimal clinical important difference (MCID), and minimal detectable change was not considered; risk of bias for studies investigating validity, reliability, and interpretability was classified by the COSMIN checklist *(Inadequate, Doubtful, Adequate, Very Good)*; studies investigating association with negative outcomes were assessed and classified by the Downs and Black checklist and PEDro *(Excellent, Good, Fair, Poor),* according to study design. Multivariable linear regression models were fitted to examine the independent association by the coefficient of determination (r^2^). Abbreviations: CF—cystic fibrosis; ILD—interstitial lung disease; PAH—pulmonary arterial hypertension; MCID—minimal clinical important difference; 1min-STS—1 min sit-to-stand; 4MGS—four-metre gait speed; 30 s-STS—30 s sit-to-stand; 5rep-STS—5-repetition sit-to-stand; SPPB—short physical performance battery; TUG—timed up-and-go; 8-FUGT—8-foot up-and-go; Glittre ADL—Glittre Activities of Daily Living; CS-PFP—continuous scale physical function performance test; 3min-STS—3 min sit-to-stand; 15-step C—15-step climbing; PPT—physical performance test; Vo_2_—maximum volume of oxygen utilisation; PFd—physical function domains; 6MWD—distance in six-minute walk test; QS—quadriceps strength; HGS—handgrip strength; rep—repetitions; total EE—total energy expenditure in daily physical activities in kilocalories; MIVC—maximal isometric voluntary contraction; D_LCO_—diffusion capacity of carbon monoxide; T_LCO_—transfer factor for carbon monoxide; FVC—forced vital capacity; mMRC—modified medical respiratory council dyspnoea score; TLC—total lung capacity; HR—hazard ratio; ICC—intraclass correlation coefficient; CI—confidence interval; ^A^—95% CI not reported in the study.

**Table 2:** Psychometric properties and associations with negative outcomes of patient-reported tools in non-COPD chronic respiratory diseases.

Patient-Report Tool	Validity	Reliability	Interpretability	Associations with Events Related to the Course of CRD or Prognosis
SF-36 (PFd or PCS)	**Asthma**: Symptoms score (r = 0.50; *p* < 0.001) (94) *(Very Good)* **ILD:** BDI (r = 0.25; *p* < 0.05); 6MWD (r = 0.44; *p* < 0.0001); mMRC (r = −0.48; *p* < 0.0001); D_LCO_ %predicted (r = 0.36; *p* < 0.001); FVC %predicted (r = 0.35; *p* < 0.05); NYHA (r = −0.33; *p* < 0.0001) [102] *(Very Good)*	**ILD:** No difference in test–retest (*p* > 0.05) [102] *(Doubtful)*	**ILD:** MCID of 4 points in PCS [102] *(Very Good)* **PAH**: MCID of 13 points in PFd and 5 points in PCS [129] *(Good)*	**Asthma**: Comorbidity (r^2^ = 0.52; *p* = 0.14); Depression (r^2^ = 0.41; *p* = 0.01) [85] *(Fair)*
SGRQ (activities)	**Bronchiectasis:** SF36–PCS (r = −0.70; *p* > 0.0001); Shuttle distance (r = −0.65; *p* < 0.0001) [143] *(Very Good)* **ILD:** (SGRQ-I)–MRC (r = 0.71; *p* < 0.0001); SF36–PFd (r = −0.71; *p* < 0.05); SF36–PCS (r = −0.32; *p* > 0.05) [98] *(Very Good)*	**ILD:** Intrarater [ICC:0.93 (95% CI: 0.85–0.97)] and inter-rater [ICC: 0.88 (95% CI: 0.77–0.94)] [98] *(Very Good)*		
WHOfc				**PAH**: WHOfc ≥ 3 points increase the risk of death [HR: 10.0 (2.9–34.1); *p* < 0.001] and clinical failure [HR: 0.04 (0.004–0.34); *p* = 0.004] [171] *(Poor)*
NYHA				**PAH**: 26% of patients in NYHA ≥ 3 died, but no statistical comparison was performed [187] *(Poor)*
AQLQ (activities)	**Asthma**: Asthma Control Questionnaire (r = 0.57; *p* < 0.05); Health Survey (Physical; r = 0.51; *p* < 0.05) [212] *(Very Good)*	**Asthma**: Test–retest (ICC:0.93) [212] *(Very Good)**^A^*	**Asthma**: MCID: 0.51 points [211] *(Poor)*	
CFQoL	**CF**: SF36–PFd (r = 0.73; *p* < 0.001) [94] *(Very Good)*	**CF**: Intrarater (0.90 < α < 0.93; r = 0.93; *p* < 0.05) [94] *(Very Good)* *^A^*		
SF-12 (PFd or PCS)				
PROMIS-29 (PFd)	**ILD:** SF-36–PCS (r = 0.52; *p* < 0.001); SF-36–PFd (r = 0.89; *p* < 0.05); SGRQ–activity (r = −0.84; *p* < 0.05); mMRC (r = −070; *p* < 0.05); HAQ-DI (r = 0.52; *p* < 0.001) [106,109] *(Very Good)*	**ILD:** Intrarater [ICC: 0.65; α = 0.92) [106,222] *(Doubtful)* *^A^*		
LWAQ (PHC)	**Asthma**: SF−36–PCS (r = 0.41; Ɨ) Symptoms score (r = 0.50; Ɨ) [88] *(Very Good)*			
QoL-EPM (PL)	**Asthma**: SF-36 domains (−0.34 < r < 0.46; *p* < 0.05) [82] *(Doubtful)*	**Asthma**: Test–retest (ICC:0.87) [82] *(Doubtful)**^A^*		
CAMPHOR (AL)			**PAH**: MCID of 4 points [180] *(Good)*	
London ADL (PAS)				
QoL-B (PFd)	**Bronchiectasis:** SGRQ–activities (r = −0.70; *p* < 0.01); mMRC (r = −0.57; *p* > 0.05); ISWT (r = 0.59; *p* > 0.05) [140] *(Very Good)*	**Bronchiectasis:** Intrarater [ICC:0.91 (95% CI: 0.86–0.93) [140] *(Very Good)*		
M-AQLQ (AL)				
MLHFQ (PSS)	**PAH**: 6MWD (r = 0.42; *p* = 0.003); NYHA (r = 0.57; *p* < 0.001) [193] *(Very Good)*	**PAH**: Test–retest (r = 0.93; *p* < 0.001; α = 0.88) [193] *(Inadequate)*		**PAH**: MLHFQ ≥ 40 points had worse prognosis (Kaplan–Meier curves comparing MLHFQ ≥ 40 points versus MLHFQ ≤ 40 points; *p* = 0.001) [193] *(Very Good)*
FPI				
SOLQ (PFS)	**Bronchiectasis:** SF-36–PCS (r = 0.53; *p* < 0.001) [90] *(Doubtful)*	**Bronchiectasis:** Test–retest (ICC:0.83; α = 0.72) [90] *(Doubtful)* *^A^*		**Bronchiectasis:** PFS of SOLQ is associated with exacerbation frequency (r^2^ = −0.20; *p* = 0.01) [90] *(Doubtful)*
ECOPS				
PROMIS-PF				
MDHAQ				
HAQ-DI				

Interpretability refers to minimal clinical important difference (MCID), and minimal detectable change was not considered; risk of bias for studies investigating validity, reliability, and interpretability was classified by the COSMIN checklist *(Inadequate, Doubtful, Adequate, Very Good)*; studies investigating association with negative outcomes were assessed and classified by the Downs and Black checklist and PEDro *(Excellent, Good, Fair and Poor)*, according to study design. Multivariable linear regression models were fitted to examine the independent association by the coefficient of determination (r^2^). Abbreviations: ILD—interstitial lung disease; PAH—pulmonary arterial hypertension; SF-36—Medical Outcomes Study 36-Item Short Form of Health Survey; SGRQ—Saint George’s Respiratory Questionnaire; WHOfc—World Health Organisation functional class; NYHA—New York Heart Association; AQLQ—Asthma Quality of Life Questionnaire; CFQoL—Cystic Fibrosis Quality of Life; SF-12—Medical Outcomes Study 12-Item Short Form of Health Survey; PROMIS-29—Patient-Reported Outcomes Measurement Information System; CAMPHOR—Cambridge Pulmonary Hypertension Outcome Review; M-AQLQ—Mini Asthma Quality of Life Questionnaire; QoL-EPM—Asthma Quality of Life from Escola Paulista de Medicina; MLHFQ—Minnesota Living with Heart Failure Questionnaire; QoL-B—Quality of Life in Bronchiectasis; SOLQ—Seattle Obstructive Lung Questionnaire; ECOPs—Eastern Cooperative Oncology Performance Status; PROMIS-PF—Patient-Reported Outcomes Measurement Information System Physical Function Short Form 8a; MDHAQ—Multidimensional Health Assessment Questionnaire; HAQ-DI—Health Assessment Questionnaire Disability Index; 6MWD—6-min walk distance; mMRC—modified Medical Respiratory Council dyspnoea score; D_LCO_—diffusion capacity of carbon monoxide; FVC—forced vital capacity; BDI—baseline dyspnoea index; FPI—functional performance inventory; PF—physical functioning; PFd—physical functioning domain; PCS—physical component score; AL—activity limitation; PHC—physical health construct; PL—physical limitation; PFS—physical functional score; PSS—physical subscore; ISWT—incremental shuttle walk test; ICC—intraclass correlation coefficient; CI—confidence interval; Ɨ—*p* value not described; HR—hazard ratio; *^A^*—95% CI not reported in the study.

**Table 3:** Summary of performance-based tests and patient-reported tool validity and reliability, with MCID reported and associated events related to the course of disease for each CRD.

CRD.	Type	Tools Investigated	Validity	Reliability	MCID	Association with Events Related to the Course of CRD
Asthma	PB test	1 min-STS	D	D	NR	NR
	PB test	5rep-STS	Y	Y	NR	NR
	PB test	30 sSTS	D	D	NR	NRmin
	PB test	4MGS	Y	D	NR	NR
	PB test	TUG	Y	Y	NR	NR
	PB test	SPPB	Y	D	NR	NR
	PR tool	PFd of SF-36	Y	NR	NR	Y, depression and number of comorbidities
	PR tool	activities of AQLQ	Y	Y	Y	NR
	PR tool	PL of QoL-EPM	Y	Y	NR	NR
	PR tool	PHC of LWAQ	Y	NR	NR	NR
Bronchiectasis	PR tool	activities of SGRQ	Y	NR	NR	NR
	PR tool	PFS of SOLQ	Y	Y	NR	Y, exacerbation frequency
	PR tool	PFd of QoL-B	Y	Y	NR	NR
Cystic Fibrosis	PB test	1 min-STS	Y	Y	Y	NR
	PB test	30 s-STS	Y	NR	NR	NR
	PR tool	CFQoL	Y	Y	NR	NR
ILD	PB test	5 rep-STS	Y	Y	NR	NR
	PB test	1 min-STS	Y	Y	NR	NR
	PB test	30 s-STS	Y	Y	NR	NR
	PB test	4MGS	Y	Y	NR	Y, hospitalisation, mortality, and disease severity
	PB test	SPPB	D	D	NR	NR
	PB test	TUG	Y	Y	NR	NR
	PB test	Glittre ADL	Y	Y	NR	NR
	PB test	CS-PFP	Y	Y	NR	NR
	PR tool	PFd of SF-36	Y	Y	Y	NR
	PR tool	activities of SGRQ-I	Y	Y	NR	NR
	PR tool	PFd of PROMIS-29	Y	Y	NR	NR
PAH	PB test	1 min-STS	Y	NR	NR	NR
	PB test	30 s-STS	Y	Y	NR	NR
	PB test	TUG	Y	Y	NR	NR
	PR tool	PFd of SF-36	NR	NR	Y	NR
	PR tool	PSS of MLHFQ	Y	Y	NR	Y, mortality, LTx, and pulmonary endarterectomy
	PR tool	WHOfc	NR	NR	NR	Y, mortality and clinical failure
	PR tool	NYHA	NR	NR	NR	Y, mortality
	PR tool	AL of CAMPHOR	NR	NR	Y	NR

Abbreviations: CRD—chronic respiratory disease; ILD—interstitial lung disease; PAH—pulmonary arterial hypertension; LTx—lung transplantation; MCID—minimal clinical important difference; Pb—performance-based tests; PR—patient-report tools; NR—not reported; Y—yes; *n*—no; D—doubtful (refers to low values reported that raise doubt); 1min-STS—1-min sit-to-stand; 4MGS—four-metre gait speed; 30 s-STS—30 s sit-to-stand; 5rep-STS—5-repetition sit-to-stand; SPPB—short physical performance battery; TUG—timed up-and-go; 8-FUGT—8-foot up-and-go; Glittre ADL—Glittre Activities of Daily Living; CS-PFP—continuous scale physical function performance test; 3 min-STS—3 min sit-to-stand; SF-36—Medical Outcomes Study 36-Item Short Form of Health Survey; SGRQ—Saint George’s Respiratory Questionnaire; WHOfc—World Health Organisation functional class; NYHA—New York Heart Association; AQLQ—Asthma Quality of Life Questionnaire; CFQoL—Cystic Fibrosis Quality of Life; SF-12—Medical Outcomes Study 12-Item Short Form of Health Survey; PROMIS-29—Patient-Reported Outcomes Measurement Information System; CAMPHOR—Cambridge Pulmonary Hypertension Outcome Review; M-AQLQ—Mini Asthma Quality of Life Questionnaire; QoL-EPM—Asthma Quality of Life from Escola Paulista de Medicina; MLHFQ—Minnesota Living with Heart Failure Questionnaire; QoL-B—Quality of Life in Bronchiectasis; SOLQ—Seattle Obstructive Lung Questionnaire; PFd—physical functioning domain; AL—activity limitation; PHC—physical health construct; PL—physical limitation; PFS—physical functional score; PSS—physical subscore.

## Data Availability

The data presented in this study are available on request from the corresponding author. All relevant data for this article can be found in the Supplementary Materials or in PROSPERO registration in this link (https://www.crd.york.ac.uk/prospero/display_record.php?RecordID=102771).

## Supplementary Materials

The following supporting information can be downloaded at: https://www.mdpi.com/article/10.3390/jcm13226887/s1, Figure S1: Number of studies per chronic respiratory disease; Table S1: Search strategy in the MEDLINE (through PubMed), EMBASE, PEDro, and Cochrane Library databases; Table S2: Description of studies included in the final screening; Table S3: Reasons for exclusion after full-text assessment and data extraction process; Table S4: Characteristics of the participants, stratified by disease, in all studies and in the studies investigating measurement properties; Table S5: Methodological quality of included studies assessed by COSMIN checklist; Table S6: Methodological quality of included randomised clinical trials assessed by PEDro; Table S7: Methodological quality of included studies assessed by Downs and Black checklist; Table S8: Functional performance protocols as reported in the included studies; Table S9: Metric properties and associations with negative outcomes of performance-based tests for only IPF patients; Table S10: Psychometric properties and associations with negative outcomes of patient-reported tools for only IPF patients.
