# Supplementary material for: Functional Measures in Non-COPD Chronic Respiratory Diseases: A Systematic Review
Source: J Clin Med. 2024 Nov 15;13(22):6887. doi: 10.3390/jcm13226887 (PMC11595047; doi:10.3390/jcm13226887)
Supplement: Supplementary file 1 [file jcm-13-06887-s001.zip › jcm-3197967-supplementary.pdf]

Supplementary material

Functional measures in non-COPD chronic respiratory diseases: a systematic review

Camile Ludovico Zamboti, PT MSc[1,2], Heloise Angélico Pimpão[1,2], Thatielle Garcia, PT MSc[3], Gabriela Garcia Krinski, PT MSc[3], Larissa Dragonetti Bertin, PT MSc[3], Sandro Laerth Souza dos Santos Filho[3], Vinicius Cavalheri, PT PhD[4,5,6]; Fabio Pitta, PT PhD[1], Carlos Augusto Camillo, PT PhD[1,2,3].

Affiliations

[1]Department of Physiotherapy, Laboratory of research in respiratory physiotherapy (LFIP), Londrina State University, Londrina, Brazil.

[2]Department of Physiotherapy in School of Science and Technology, Sao Paulo State University (UNESP), Presidente Prudente, Sao Paulo, Brazil.

[3]Research Center in Health Sciences, University Pitágoras UNOPAR, Londrina, Brazil.

[4]Curtin School of Allied Health and enAble Institute, Faculty of Health Sciences, Curtin University; Bentley, Perth, Western Australia.

[5]Allied Health, South Metropolitan Health Service, Perth, Western Australia.

[6]Exercise Medicine Research Institute, Edith Cowan University, Perth, Australia.

E-mail for correspondence: carlos.a.camillo@outlook.com

Summary

Table S1. Search strategy in MEDLINE (through PubMed), EMBASE, PEDro and the Cochrane Library databases. 3

Table S2. Description of studies included in the final screening. 5

Table S3. Reason for exclusion after full text assessment and data extraction process. 27

Table S4. Characteristics of the participants stratified by disease in all studies and in the studies investigating measurement properties. 33

Table S5. Methodological quality of included studies assessed by Cosmin checklist. 34

Table S6. Methodological quality of randomized clinical trials included assessed by Pedro. 36

Table S7. Methodological quality of included studies assessed by Downs and Black checklist. 41

Table S8. Functional performance protocols as per reported in the included studies. 44

Table S9. Metric properties and associations with negative outcomes of performance-based tests for only IPF patients. 46

Table S10. Psychometric properties and associations with negative outcomes of patient-reported tools for only IPF patients. 48

Figure S1. Number of studies per chronic respiratory disease. 49

Information regarding patient-reported and performance-based instruments in patients pre and post lung transplantation (LTx). 50

Description of the performance-based and patient-report tools, as well as their properties and clinical implications 51

References 58

Table S1. Search strategy in MEDLINE (through PubMed), EMBASE, PEDro and the Cochrane Library databases.

PUBMED		
**Search**	**Concept**	**Query**
#1	**Participants**	Lung (Title/Abstract) OR Pulmonary (Title/Abstract) OR Respiratory (Title/Abstract) OR Cystic Fibrosis (Title/Abstract)
#2	**Outcome**	Walk speed (Title/Abstract) OR Gait speed (Title/Abstract) OR Sit-to-stand (Title/Abstract OR 4MGS (Title/Abstract) OR Muscle dysfunction (Title/Abstract) OR Physical function (Title/Abstract) OR Physical performance (Title/Abstract) OR Functional capacity (Title/Abstract) OR Physical fitness (Title/Abstract)
#3		#1 AND #2
**EMBASE**		
#1	**Participants**	Lung (Title/Abstract) OR Pulmonary (Title/Abstract) OR Respiratory (Title/Abstract) OR Cystic Fibrosis (Title/Abstract)
#2	**Outcomes**	Walk speed (Title/Abstract) OR Gait speed (Title/Abstract) OR Sit-to-stand (Title/Abstract OR 4MGS (Title/Abstract) OR Muscle dysfunction (Title/Abstract) OR Physical function (Title/Abstract) OR Physical performance (Title/Abstract) OR Functional capacity (Title/Abstract) OR Physical fitness (Title/Abstract)
#3		#1 AND #2
**PEDro**		
**1**	Abstract & Title	Lung* *Walk Speed
**2**	Abstract & Title	Pulmonary* *Walk Speed
**3**	Abstract & Title	*Respiratory *Walk Speed
**4**	Abstract & Title	Lung* *Gait Speed
**5**	Abstract & Title	Pulmonary* * Gait Speed
**6**	Abstract & Title	*Respiratory *Gait Speed
**7**	Abstract & Title	Lung* *Sit-to-stand
**8**	Abstract & Title	Pulmonary* *Sit-to-stand
**9**	Abstract & Title	*Respiratory *Sit-to-stand
**10**	Abstract & Title	Lung* 4MGS
**11**	Abstract & Title	Pulmonary* 4MGS
**12**	Abstract & Title	*Respiratory 4MGS
**13**	Abstract & Title	Lung* Muscle dysfunction
**14**	Abstract & Title	Pulmonary* Muscle dysfunction
**15**	Abstract & Title	*Respiratory Muscle dysfunction
**16**	Abstract & Title	Lung* Physical function

**17**	Abstract & Title	Pulmonary* Physical function
**18**	Abstract & Title	*Respiratory Physical function
**19**	Abstract & Title	Lung* Physical performance
**20**	Abstract & Title	Pulmonary* Physical performance
**21**	Abstract & Title	*Respiratory Physical performance
**22**	Abstract & Title	Lung* Functional capacity
**23**	Abstract & Title	Pulmonary* Functional capacity
**24**	Abstract & Title	*Respiratory Functional capacity
**25**	Abstract & Title	Lung* Physical fitness
**26**	Abstract & Title	Pulmonary* Physical fitness
**27**	Abstract & Title	*Respiratory Physical fitness
Note: Keyword groups were linked by AND.		
**Cochrane Library databases**		
#1	**Participants**	Lung (Title, Abstract, Keyword) OR Pulmonary (Title, Abstract, Keyword) OR Respiratory (Title, Abstract, Keyword) OR Cystic Fibrosis (Title, Abstract, Keyword)
#2	**Outcomes**	Walk speed (Title, Abstract, Keyword) OR Gait speed (Title, Abstract, Keyword) OR Sit-to-stand (Title, Abstract, Keyword) OR 4MGS (Title, Abstract, Keyword) OR Muscle dysfunction (Title, Abstract, Keyword) OR Physical function (Title, Abstract, Keyword) OR Physical performance (Title, Abstract, Keyword) OR Functional capacity (Title, Abstract, Keyword) OR Physical fitness (Title, Abstract, Keyword)
#3		#1 AND #2
	**Publication year and dates**	All years and dates
	**Content type**	No restriction

Table S2. Description of studies included in the final screening.

First Author (ref)	Design of study	Disease	Age	Sex (F/M)	Complete n (% of initial)	Protocol
One-minute Sit-to-Stand						
JM Oliveira et al., 2020 [1]	Cross-sectional	Asthma	47 [38-58]	32/20	52 (100%)	"…the participants should stand up and sit down as many times as possible for 1min with results in number of repetitions."
FC Freitas et al., 2012 [2]	Cross-sectional	Asthma	49.8 (8.1)	9/3	12 (100%)	"Sit-to-stand as fast as possible in one minute"
T Radtke et al., 2017 [3]	Cross-sectional	Cystic Fibrosis	31 [25-33]	7/8	15 (100%)	"Sit-to-stand as fast as possible in one minute with 3-min rest"
T Radtke et al., 2016 [4]	Interventional	Cystic Fibrosis	29 [25-36]	8/6	14 (100%)	ND
M Gruet et al., 2016 [5]	Cross-sectional	Cystic Fibrosis	30 (9)	8/17	25	"Subjects were instructed to complete as many Sit-to-Stand cycles as possible within 1 min at a self-paced speed"
K Oishi et al., 2023 [6]	Cross-sectional	ILD	72 [64-78]	52/62	116 (100%)	ND
C Paixão et al., 2023 [7]	Interventional	ILD	77 (3)	5/5	9 (90%)	"…participants were asked to sit and stand as many times as possible during one minute."
K Oishi et al., 2022 [8]	Cross-sectional	ILD	72 [64-78]	52/62	116 (100%)	"Patients were asked to perform repetitions of standing upright and then sitting down in the same position at a self-paced motion (safe and comfortable) for as many repetitions as possible in 1 min."
PF Tremblay Labrecque et al., 2022 [9]	Cross-sectional	ILD	70 (7)	8/28	36 (100%)	"The participant was instructed to stand-up completely and to sit back down as many times possible within one minute…"

First Author (ref)	Design of study	Disease	Age	Sex (F/M)	Complete n (% of initial)	Protocol
CL Zamboti et al., 2021 [10]	Cross-sectional	ILD	60.8 (11)	25/16	46 (86%)	"…participants were requested to perform the highest number of sit-to-stand movements during 1minute…"
A Fedi et al., 2021 [11]	Cross-sectional	ILD	60 (6)	5/28	33 (89%)	"…patients were asked to perform as many repetitions as possible in 1 minute."
PF Tremblay Labrecque et al., 2020 [12]	Cross-sectional	ILD	69 (7)	5/10	15 (88%)	"The participant was instructed to stand-up completely and to sit back down as many times possible within one minute…"
B Wallaert et al., 2020 [13]	Cross-sectional	ILD	57 (14)	45/62	107 (100%)	"…as many times as possible at a self-paced speed (safe and comfortable) for 1 minute, …"
J Briand et al., 2018 [14]	Cross-sectional	ILD	57 (14)	45/62	107 (100%)	"…standing upright and then sitting down at a self-paced speed as many times as possible for 1 min"
C Keen et al., 2023 [15]	Longitudinal, Prospective	PAH	53.9 (14.9)	47/13	60 (100%)	"Participants were instructed to stand up and sit down as many times as they could within 1 min, without using their arms."
C Kronberger et al., 2023 [16]	Cross-sectional	PAH	66 (15)	66/40	106 (100%)	"Participants were requested... to perform stand-and-sit transitions on the chair as many times as possible within one minute."
M Pereira et al., 2022 [17]	Cross-sectional	PAH	44.3 (13.2)	4/16	20 (100%)	"Over the course of one minute, the patient must sit and stand up from the chair repeatedly, as quickly as possible."
L Nakazato et al., 2020 [18]	Cross-sectional	PAH	44.3 (13.2)	16/4	20 (100%)	"…involves the performance of as many sit-to-stand actions as possible in one minute without using the upper limbs"
5 repetitions Sit-to-Stand						
E Zampogna et al., 2021 [19]	Longitudinal, Retrospective	Asthma	70.3 (8.6)	62/41	103 (100%)	"Stand up all the way and sit down until the back rests against the seatback without

First Author (ref)	Design of study	Disease	Age	Sex (F/M)	Complete n (% of initial)	Protocol
						use of the upper limbs; repeat 5 times, as fast as possible, starting when I say, Go.”
A Yilmaz et al., 2021 [20]	Cross-sectional	Asthma	35.5 (10.3)	39/10	49 (100%)	“The participant is raised to a complete stand and returns to the initial position. The duration was recorded.”
JM Oliveira et al., 2020 [1]	Cross-sectional	Asthma	47 [38-58]	32/20	52 (100%)	“…stopwatch started on the command “go” and stopped at the end of the completed fifth stand, with results in seconds.”
O Atalay et al., 2022 [21]	Interventional	Bronchiectasis	55.6 (17.1)	ND	20 (100%)	“were asked to stand up and sit down quickly five times from a standard chair with a height of 43 cm.”
Z McKeough et al., 2020 [22]	Cross-sectional	Bronchiectasis	74 (8)	16/17	33 (100%)	“…measures the time taken to stand five times as quickly as possible...”
PF Tremblay Labrecque et al., 2022 [9]	Cross-sectional	ILD	70 (7)	8/28	36 (100%)	“fastest time to complete 5 times sit-to-stand.”
CL Zamboti et al., 2021 [10]	Cross-sectional	ILD	60.8 (11)	25/16	46 (86%)	“…the time necessary to perform 5 movements was recorded...”
AR Koczulla et al., 2020 [23]	Interventional	ILD	62 (49-74)	5/6	11 (78%)	“Subjects were asked to stand up and sit down 5 times as quickly as possible with their arms folded across their chest.”
JN Justice et al., 2019 [24]	Interventional	ILD	70.8 [55-84]	2/12	14 (100%)	“To evaluate time to complete 5-repition chair-stands, participants were asked to stand up and sit down on a straight-backed chair five times, as quickly as possible without using their arms.”
AEM Bloem et al., 2018[c] [25]	Cross-sectional	ILD	68 [63-74]	14/37	42 (82%)	“…to stand up and sit down 5 times, as quickly as they could without any form of assistance”
P Mendes et al., 2015 [26]	Cross-sectional	ILD	61 (8)	7/19	26 (100%)	ND

First Author (ref)	Design of study	Disease	Age	Sex (F/M)	Complete n (% of initial)	Protocol
M Okamura et al., 2022 [27]	Cross-sectional	PAH	50.7 (16.2)	15/7	22 (100%)	"…five rotations of sit-to-stand as quickly as possible without the use of the arms".
L González-Saiz et al., 2017 [28]	Interventional	PAH	46 (11)	12/8	20 (100%)	ND
30 second Sit-to-Stand						
JM Oliveira et al., 2020 [1]	Cross-sectional	Asthma	47 [38-58]	32/20	52 (100%)	"…the participants should stand up and sit down as many times as possible for 30s with results in number of repetitions."
M Majewski et al., 2015 [29]	Interventional	Asthma	70.8	10/0	10 (91%)	ND
E Sheppard et al., 2019 [30]	Cross-sectional	Cystic Fibrosis	32 (13)	6/9	15 (100%)	"The participant was instructed to stand from a seated position as many times as possible in 30 s."
SY Chikina et al., 2022 [31]	Cross-sectional	ILD	62.5 (16.2)	14/11	25 (100%)	"… the patient hands-free (with arms crossed) for 30s get up from a 46cm chair and dit back with a maximum possible frequency."
CL Zamboti et al., 2021 [10]	Cross-sectional	ILD	60.8 (11)	25/16	46 (86%)	"…participants were requested to perform the highest number of sit-to-stand movements during 30seconds…"
B Vainshelboim et al., 2019 [32]	Longitudinal, Prospective	ILD	68 (8)	12/22	34 (100%)	"Patients were encouraged to complete as many full stands as possible from a sitting position on a chair during 30s."
B Vainshelboim et al., 2015 [33]	Interventional	ILD	68.8 (6)	5/10	15 (100%)	"…as many full stands as possible from the sitting position on the chair within the 30 s"
B Vainshelboim et al., 2014 [34]	Interventional	ILD	68.8 (6)	5/10	15 (100%)	"…as many full stands as possible from the sitting position on the chair within the 30 s"
B Kahraman et al., 2020 [35]	Interventional	PAH	50.2 (18.0)	7/31	38 (100%)	"…were asked to rise from a seated position and sit as quickly and safely as possible in 30seconds."

First Author (ref)	Design of study	Disease	Age	Sex (F/M)	Complete n (% of initial)	Protocol
B Kahraman et al., 2020 [36]	Cross-sectional	PAH	52.5 [25-62]	3/9	11 (73%)	ND
3-minute Sit-to-Stand						
A Fedi et al., 2021 [11]	Cross-sectional	ILD	60 (6)	5/28	33 (89%)	"...the number of chair rises was imposed during the first minute (12 or 20, depending on the investigator's appreciation of the patient's fitness), and patients were asked to repeat the movement as many times as possible in the next 2 minutes."
Four-metre Gait Speed						
I Ozsoy et al., 2022 [37]	Cross-sectional	Asthma	52.9 (15.2)	52/5	57 (100%)	"... to walk at their normal speed along an 8-meter-long hallway, divided into three zones: acceleration zone (2 m), central "testing" zone (4 m), and deceleration zone (2 m)."
JM Oliveira et al., 2020 [1]	Cross-sectional	Asthma	47 [38-58]	32/20	52 (100%)	"…the participants walk in a 4 m corridor… and were instructed to walk in a usual pace, …and also in a fast-pace walking mode, … fast as possible without run."
Z McKeough et al., 2020 [22]	Cross-sectional	Bronchiectasis	74 (8)	16/17	33 (100%)	"…over a 4m flat straight walking track with participants instructed to walk at their usual speed."
MDM Martinez-Garcia et al., 2020 [38]	Cross-sectional	Cystic Fibrosis	32.4 (12.4)	17/21	38 (92%)	"Subjects were instructed to walk at their normal comfortable pace. Two meters were provided prior to and following the timed portion to allow for acceleration and deceleration phases."

First Author (ref)	Design of study	Disease	Age	Sex (F/M)	Complete n (% of initial)	Protocol
CM Nolan et al., 2023 [39]	Longitudinal, Prospective	ILD	72 (6)	82/3	85 (100%)	"to walk… your usual speed, just as if you were walking down the street to go to the shops."
CL Zamboti et al., 2021 [10]	Cross-sectional	ILD	60.8 (11)	25/16	46 (86%)	"…participants were required to walk in a usual gait speed measured over 4 m."
R Hirabayashi et al., 2020 [40]	Cross-sectional	ILD	74.2 (7.5)	35/16	51 (100%)	"Patients walked 4m…"
JN Justice et al., 2019 [24]	Interventional	ILD	70.8 [55-84]	2/12	14 (100%)	"…asking the participants to walk at their usual pace over a 4 m course, with the faster of two walks used to compute walking speed."
SA Guler et al., 2019[a] [41]	Cross-sectional	ILD	65.5 (9.5)	44/71	115 (70%)	"…to walk at their usual pace along a 4-m course…"
CM Nolan et al., 2018 [42]	Cross-sectional	ILD	75 (7.6)	13/33	46 (100%)	"…4-m course … with participants walking at their usual speed…"
AEM Bloem et al., 2018[c] [25]	Cross-sectional	ILD	68 [63-74]	14/37	42 (82%)	"...to walk as quickly as possible 4 meters (moving start) …"
CM Nolan et al., 2018 [43]	Longitudinal, Prospective	ILD	72 (7)	22/108	130 (98%)	"to walk… your usual speed, just as if you were walking down the street to go to the shops."
CJ Ryerson et al., 2014 [44]	Longitudinal, Prospective	ILD	69.4 (10.8)	28/26	54 (100%)	"… to walk 4m at their normal pace."
M Okamura et al., 2022 [27]	Cross-sectional	PAH	50.7 (16.2)	15/7	22 (100%)	"…4 m at the subjects'normal pace."
Short Physical Performance Battery						
HA Babar et al., 2022 [45]	Cross-sectional	Asthma	ND	17/13	30 (100%)	"The subjects have been requested to perform balance test, gait speed test and 5 repetitions of sit to stand test..."
JM Oliveira et al., 2020 [1]	Cross-sectional	Asthma	47 [38-58]	32/20	52 (100%)	"…balance assessment, walking speed at usual pace, and sitting and rising in the chair 5 times."

First Author (ref)	Design of study	Disease	Age	Sex (F/M)	Complete n (% of initial)	Protocol
M Hanada et al., 2022 [46]	Cross-sectional	ILD	71 [67-77]	51/27	78 (100%)	"…consists of three measures: walking speed, chair stands, and standing balance."
PF Tremblay Labrecque et al., 2022 [9]	Cross-sectional	ILD	70 (7)	8/28	36 (100%)	"... consists of the sum of three separate functional components: time to complete 5 times sit-to-stand, 10-s static standing balance test and 4-m walk test."
CL Zamboti et al., 2021 [10]	Cross-sectional	ILD	60.8 (11)	25/16	46 (86%)	"…composite of 3 balance tests, a gait speed test and sit-to-stand test."
JN Justice et al., 2019 [24]	Interventional	ILD	70.8 (55-84)	2/12	14 (100%)	"…performance on 4 m gait speed and chair-stands tests and a balance test were scored and combined to derive the summary SPPB Score."
P Mendes et al., 2015 [26]	Cross-sectional	ILD	61 (8)	7/19	26 (100%)	Includes sit-to-stand, gait speed and tandem stance for balance.
M Okamura et al., 2022 [27]	Cross-sectional	PAH	50.7 (16.2)	15/7	22 (100%)	"... consists of a gait speed test (4 m at the subjects' normal pace), a chair stand test (five rotations of sit-to-stand as quickly as possible without the use of the arms), and a balance test (10 s in side-by-side, semi-tandem, and tandem positions)."
AA Perez et al., 2020 [47]	Longitudinal, Prospective	post-LTx	31 (7.9)	12/15	27 (90%)	"The SPPB has three timed components: chair stands, balance, and gait speed. Each component of the SPPB has a score range of 0–4 with an aggregate range of 0–12."
Timed Up and Go						
A Yilmaz et al., 2021 [20]	Cross-sectional	Asthma	35.5 (10.3)	39/10	49 (100%)	ND

First Author (ref)	Design of study	Disease	Age	Sex (F/M)	Complete n (% of initial)	Protocol
JM Oliveira et al., 2020 [1]	Cross-sectional	Asthma	47 [38-58]	32/20	52 (100%)	"…two protocols of this test were performed, with participants walking at their usual pace and at the fastest possible pace"
N Vardar-Yagli et al., 2022 [48]	Cross-sectional	Bronchiectasis	46.2 (5.4)	8/14	22 (100%)	"… stand from a standardized armchair, walking 3 m at their usual speed, turning, walking back to the chair, and returning to the seated position."
CL Zamboti et al., 2021 [10]	Cross-sectional	ILD	60.8 (11)	25/16	46 (86%)	"…participants performed it at usual walking pace, and as fast possible without running."
P Mendes et al., 2015 [26]	Cross-sectional	ILD	61 (8)	7/19	26 (100%)	ND
B Kahraman et al., 2020 [35]	Interventional	PAH	50.2 (18.0)	7/31	38 (100%)	"Participants were stand up from the chair, walked 3 m at a comfortable pace, turn and walk back to the starting point, and sit down again."
B Kahraman et al., 2020 [36]	Cross-sectional	PAH	52.5 (25-62)	3/9	11 (73%)	ND
8-Foot Up and Go Test						
M Majewski et al., 2015 [29]	Interventional	Asthma	70.8	10/0	10 (91%)	ND
B Vainshelboim et al., 2019 [32]	Longitudinal, Prospective	ILD	68 (8)	12/22	34 (100%)	"…the patient got up from the chair, walked around a cone that was placed 8feet (2.40m) from the chair, and returned to a seated position on the chair as fast as possible."
P Mendes et al., 2015 [26]	Cross-sectional	ILD	61 (8)	7/19	26 (100%)	ND
Glittre ADL						
R Hena et al., 2018 [49]	Cross-sectional	Bronchiectasis	50.8 (11.5)	12/3	15 (100%)	"…consists of a standardized 10-meter circuit, where the individual was

First Author (ref)	Design of study	Disease	Age	Sex (F/M)	Complete n (% of initial)	Protocol
						instructed to go through the following sequence of activities in the shortest time."
GP Reinaldo et al., 2022 [50]	Cross-sectional	ILD	60.7 (10.7)	19/8	27 (100%)	"consists in performing five laps of a standardized circuit... The participants were instructed to complete the test as quickly as possible."
HF Alexandre et al., 2021 [51]	Cross-sectional	ILD	63.2 (11.4)	ND	21 (75%)	"…consists of a 10-meter circuit in which the individual starts from a sitting position, walks, goes up and down two interposed steps and walks again until reaching a shelf, individually adjusted according to the height of the shoulder and waist."
Continuous Scale – Physical Function Performance						
AL Olson et al., 2015 [52]	Cross-sectional	ILD	69.3 (9.2)	7/9	16 (100%)	"…is a series of 10 tasks covering everyday life activities required to maintain independence"
15-steps Climbing						
V Rusanov et al., 2008 [53]	Cross-sectional	ILD	58 (11)	22/29	51 (100%)	"…patients were asked to climb up and down the step 15 times as fast as they could, without any fixed pacing."
Physical Performance Test						
C Paixão et al., 2023 [7]	Interventional	ILD	77 (3)	5/5	9 (90%)	"The PPT assesses multiple domains of individuals' functional capacity using tasks that simulate ADLs, and has two versions: 7- and 9-items."
**Subjective measures**						
SF-36 – Physical Functioning or Physical Component Summary						
CM Olivera et al., 2016 [54]	Interventional	Asthma	52 (10)	15/37	52 (88%)	ND

First Author (ref)	Design of study	Disease	Age	Sex (F/M)	Complete n (% of initial)	Protocol
N Newhouse et al., 2016 [55]	Interventional	Asthma	58.2 (11.7)	27/46	62 (84%)	ND
A Meyer et al., 2015 [56]	Interventional	Asthma	54.0 (11.0)	5/8	13 (92%)	Self-administered
HJ Pai et al.2015 [57]	Interventional	Asthma	32.5	ND	31 (100%)	Self-administered
U Ochmann et al., 2012 [58]	Interventional	Asthma	64	ND	121 (100%)	ND
AM Smith et al., 2012 [59]	Longitudinal[d]	Asthma	68.7 (7.2)	59/18	77 (100%)	ND
S Turner et al., 2011 [60]	Interventional	Asthma	65.8 (10.8)	11/8	19 (95%)	ND
B Kligler et al., 2011 [61]	Interventional	Asthma	43.4 (11.8)	61/16	77 (100%)	ND
V Siroux et al., 2008[c] [62]	Cross-sectional	Asthma	42.4 (7.3)	524/376	864 (96%)	ND
Y Tohda et al., 2006 [63]	Interventional	Asthma	57.9 (15.3)	16/28	44 (100%)	ND
DK McClish et al., 2005 [64]	Cross-sectional	Asthma	38	168/133	301 (100%)	ND
MA De Oliveira et al., 2005 [65]	Longitudinal, Prospective	Asthma	28.2 (11.3)	19/6	35 (100%)	Self-administered
M Matheson et al., 2012 [66]	Cross-sectional	Asthma	39.7 (6.4)	213/213	426	Self-administered
K Stavem et al., 2000 [67]	Cross-sectional	Asthma	47 [16-88]	82/35	117 (100%)	Self-administered
CA Dyer et al., 1999 [68]	Cross-sectional	Asthma	77 [70-91]	33/27	60 (100%)	Self-administered
J Ware et al., 1998 [69]	Interventional	Asthma	39.5	89/57	146 (82%)	Self-administered
P Blanc et al., 1997[c] [70]	Longitudinal, Prospective	Asthma	40 (8)	199/84	283 (90%)	Assessor applied
T Van der Molen et al., 1997 [71]	Cross-sectional	Asthma	44 (14)	56/54	110 (100%)	Self-administered
LI Okamoto et al., 1996 [72]	Interventional	Asthma	50	10/22	32 (100%)	Self-administered
E Bulcun et al., 2015 [73]	Cross-sectional	Bronchiectasis	48.1 (13.5)	46/32	78 (100%)	ND
PS Jacques et al., 2012 [74]	Cross-sectional	Bronchiectasis	40.9	18/5	23 (100%)	ND
AL Lee et al., 2009 [75]	Cross-sectional	Bronchiectasis	54.4 (13.8)	16/11	27 (100%)	ND
JM Guilemany et al., 2006 [76]	Cross-sectional	Bronchiectasis	52 (16)	39/21	60 (100%)	Self-administered
DK Mcclish et al., 2005[a] [64]	Cross-sectional	Cystic Fibrosis	25.0	120/103	223 (100%)	ND

First Author (ref)	Design of study	Disease	Age	Sex (F/M)	Complete n (% of initial)	Protocol
L Gee et al., 2000 [77]	Cross-sectional	Cystic Fibrosis	27 [16-53]	15/17	32 (100%)	ND
M Sikora et al., 2023 [78]	Cross-sectional	ILD	60.9 (10.9)	18/34	52 (100%)	Assessor applied
F Aboelmagd et al., 2022 [79]	Interventional	ILD	46.5 [45-53]	22/8	30 (93%)	Assessor applied
F Machado et al., 2021 [80]	Cross-sectional	ILD	68 (8)	15/84	98 (100%)	ND
WF Aguiar et al., 2021 [81]	Cross-sectional	ILD	59 (10)	15/15	30 (100%)	Assessor applied
R Zhao et al., 2020 [82]	Cross-sectional	ILD	47.3 (15.1)	26/2	28 (100%)	ND
S Dalichau et al., 2020 [83]	Interventional	ILD	73.7 (5.3)	ND	44 (72%)	ND
AR Koczulla et al., 2020 [23]	Interventional	ILD	62 [49-74]	5/6	11 (78%)	ND
R Vis et al., 2020 [84]	Interventional	ILD	40.6 (7.6)	4/3	7 (100%)	Self-administered
S Witt et al., 2019 [85]	Cross-sectional	ILD	67.3(10.7)	69/189	258 (100%)	Assessor applied
E Yalnız et al., 2019 [86]	Cross-sectional	ILD	67.4(7.1)	38/12	50(100%)	ND
K Pilzak et al., 2018 [87]	Cross-sectional	ILD	46.8(8.8)	7/10	17 (100%)	Self-administered
I Naz et al., 2018 [88]	Interventional	ILD	59 [52-64]	6/3	9 (100%)	ND
AEM Bloem et al., 2018[c] [25]	Cross-sectional	ILD	68 [63-74]	14/37	42 (82%)	Self-administered
CJ Fisher et al., 2019 [89]	Cross-sectional	ILD	51.9 (11.8)	59/14	73 (100%)	Assessor applied
H Tomioka et al., 2016 [90]	Interventional	ILD	76.5(7.1)	2/15	17 (100%)	ND
S Dalichau et al., et al., 2010 [91]	Interventional	ILD	65.7(5.5)	0/104	104 (100%)	ND
ME Hinchcliff et al., et al., 2015 [92]	Cross-sectional	ILD	52 [27-71]	90/10	100 (100%)	Self-administered
EH Alhamad et al., et al., 2015 [93]	Interventional	ILD	63.3(13.3)	11/22	33 (82%)	Self-administered
AL Olson et al., et al., 2015 [52]	Cross-sectional	ILD	69.3(9.2)	7/9	16 (100%)	Self-administered
RM Du bois et al., 2011 [94]	Interventional	ILD	65.3 (8.1)	344/812	1165 (100%)	ND
F Lumetti et al., 2015 [95]	Cross-sectional	ILD	63.3 (11.7)	47/1	48 (100%)	Self-administered
U Ochmann et al., 2012[b] [58]	Interventional	ILD	64	ND	108 (100%)	ND

First Author (ref)	Design of study	Disease	Age	Sex (F/M)	Complete n (% of initial)	Protocol
AC Theodore et al., 2012 [96]	Interventional	ILD	ND	ND	114 (100%)	Self-administered
JJ Swigris et al., 2012 [97]	Interventional	ILD	69 (9)	31/149	180 (100%)	ND
JJ Swigris et al., 2011 [98]	Interventional	ILD	71.5 (7.4)	3/18	21 (100%)	Self-administered
V Krishnan et al., 2008 [99]	Cross-sectional	ILD	67.7 (8.7)	19/22	41 (85%)	ND
C Zimmermann et al., 2007 [100]	Cross-sectional	ILD	61.4 (10.5)	8/12	20 (100%)	ND
S Ohno et al., 2005 [101]	Cross-sectional	ILD	66.6 (6.7)	12/28	40 (100%)	Self-administered
JA Chang et al., 1999 [102]	Cross-sectional	ILD	60.5 [29-81]	22/28	50 (100%)	ND
JF Masa et al., 2022 [103]	Cross-sectional	PAH	66 [56-72]	79/43	122 (100%)	ND
L Nakazato et al., 2020 [18]	Cross-sectional	PAH	44.3 (13.2)	16/4	20 (100%)	ND
H Karapolat et al., 2019 [104]	Interventional	PAH	34 (26-62)	3/12	12 (80%)	ND
AS Babu et al., 2019 [105]	Interventional	PAH	51.4 (13.7)	20/22	34 (80%)	ND
M Kukkonen et al., 2016 [106]	Cross-sectional	PAH	53 (16.2)	46/32	78 (100%)	ND
SC Mathai et al., 2015[c] [107]	Interventional	PAH	55 (15)	317/88	405 (100%)	ND
ID Laoutaris et al., 2015 [108]	Interventional	PAH	48.6 (12.7)	4/1	5 (100%)	ND
LA Matura et al., 2014 [109]	Cross-sectional	PAH	53.5 (15.1)	127/22	149 (100%)	Self-administered
LA Matura et al., 2012 [110]	Cross-sectional	PAH	52.2 (16.0)	76/17	93	ND
E Grunig et al., 2012 [111]	Interventional	PAH	53 (15)	126/57	183(94%)	Self-administered
C Gilbert et al., 2009 [112]	Interventional	PAH	ND	ND	278	ND
J Pepke-Zaba et al., 2009 [113]	Interventional	PAH	53 (15)	59/20	71 (89%)	Self-administered
N Galie et al., 2008 [114]	Interventional	PAH	52 (15)	48/16	64 (100%)	ND
R Souza et al., 2007 [115]	Cross-sectional	PAH	41 (11)	17/6	23 (100%)	Assessor applied
D Mereles et al., 2006 [116]	Interventional	PAH	53 (14)	10/5	15 (100%)	Self-administered
J White et al., 2006 [117]	Cross-sectional	PAH	48.6 (11.8)	23/4	27 (100%)	ND

First Author (ref)	Design of study	Disease	Age	Sex (F/M)	Complete n (% of initial)	Protocol
R Souza et al., 2005 [118]	Cross-sectional	PAH	40 (11)	11/4	15 (100%)	Assessor applied
A Miozzo et al., 2023 [119]	Longitudinal, Retrospective	pre-LTx	45	26/19	45 (100%)	ND
D Langer et al., 2012 [120]	Cross-sectional	post-LTx	51 (10)	15/12	27 (100%)	ND
MI Feltrim et al., 2008 [121]	Cross-sectional	pre-LTx	54 (11)	3/4	7 (100%)	Assessor applied
SGRQ – Activities						
N Duruturk et al., 2018 [122]	Interventional	Asthma	46.5 (13.3)	14/6	16 (80%)	ND
M Majewski et al., 2015 [29]	Interventional	Asthma	70.8	10/0	10 (91%)	ND
MAM Zadeh et al., 2013 [123]	Interventional	Asthma	22	0/33	33 (91%)	Self-administered
CA Dyer et al., 1999 [68]	Cross-sectional	Asthma	77 [70-91]	33/27	60 (100%)	Self-administered
O Atalay et al., 2022 [21]	Interventional	Bronchiectasis	55.6 (17.1)	ND	20 (100%)	ND
Z McKeough et al., 2020 [22]	Cross-sectional	Bronchiectasis	74 (8)	16/17	33 (100%)	Self-administered
CO De Camargo et al., 2020 [124]	Cross-sectional	Bronchiectasis	48 (14.1)	61/57	108 (125%)	Self-administered
KA Lavery et al., 2011[c] [125]	Interventional	Bronchiectasis	60 (9)	17/15	30 (93%)	ND
AL Lee et al., 2009 [75]	Cross-sectional	Bronchiectasis	54.4 (13.8)	16/11	27 (100%)	ND
MA Martinez-Garcia et al., 2006 [126]	Interventional	Bronchiectasis	70.9 (6)	8/21	29 (93%)	ND
C Wilson et al., 1997 [127]	Longitudinal[d]	Bronchiectasis	52.4 (12.8)	67/44	111 (100%)	Self-administered
S Zaki et al., 2023 [128]	Interventional	ILD	53.3 (13.6)	14/12	22 (84%)	ND
GP Reinaldo et al., 2022 [50]	Cross-sectional	ILD	60.7 (10.7)	19/8	27 (100%)	ND
H Essam et al., 2022 [129]	Interventional	ILD	44.4 (12.2)	4/6	9 (90%)	ND
K Fujita et al., 2022 [130]	Cross-sectional	ILD	73.1 (7.7)	7/49	56 (100%)	ND
PF Tremblay Labrecque et al., 2022 [9]	Cross-sectional	ILD	70 (7)	8/28	36 (100%)	ND
WF Aguiar et al., 2021 [81]	Cross-sectional	ILD	59 (10)	15/15	30 (100%)	Assessor applied

First Author (ref)	Design of study	Disease	Age	Sex (F/M)	Complete n (% of initial)	Protocol
HF Alexandre et al., 2021 [51]	Cross-sectional	ILD	63.2 (11.4)	ND	21 (75%)	ND
K Ebihrara et al., 2021 [131]	Cross-sectional	ILD	76.1 (5.9)	6/21	27 (56%)	ND
K Janssen et al., 2020 [132]	Interventional	ILD	72.7 (8)	0/11	11 (100%)	ND
R Hirabayashi et al., 2020 [40]	Cross-sectional	ILD	74.2 (7.5)	35/16	51 (100%)	Self-administered
AR Koczulla et al., 2020 [23]	Interventional	ILD	62 [49-74]	5/6	11 (78%)	ND
JN Justice et al., 2019 [24]	Interventional	ILD	70.8 [55-84]	2/12	14 (100%)	ND
AW Creamer et al., 2019 [133]	Interventional	ILD	ND	ND	41 (100%)	Self-administered
PV Santana et al., 2019 [134]	Cross-sectional	ILD	54 (14)	4/5	9 (100%)	ND
I Naz et al., 2018 [88]	Interventional	ILD	59 [52-64]	6/3	9 (100%)	ND
CJ Fisher et al., 2019 [89]	Cross-sectional	ILD	51.9 (11.8)	59/14	73 (100%)	Assessor applied
LM Dowman et al., 2017 [135]	Interventional	ILD	70 (9.6)	18/34	48 (92%)	Assessor applied
NF Braz et al., 2016 [136]	Cross-sectional	ILD	43.4 (9.7)	0/23	23 (88%)	Assessor applied
MN Karadalli et al., 2016 [137]	Interventional	ILD	45.1 (8.1)	10/5	15 (88%)	ND
AL Olson et al., 2015 [52]	Cross-sectional	ILD	69.3 (9.2)	7/9	16 (100%)	Self-administered
B Vainshelboim et al., 2015 [33]	Interventional	ILD	68.8 (6)	5/10	15 (100%)	Self-administered
B Vainshelboim et al., 2014 [34]	Interventional	ILD	68.8 (6)	5/10	15 (100%)	Self-administered
WP Drake et al., 2013 [138]	Interventional	ILD	54 [43-65]	11/4	8 (53%)	ND
JJ Swigris et al., 2012 [97]	Interventional	ILD	69 (9)	31/149	180 (100%)	ND
ZÇ Sozener et al., 2010 [139]	Cross-sectional	ILD	52.7 (9.8)	28/5	33 (100%)	Self-administered
C Zimmermann et al., 2007 [100]	Cross-sectional	ILD	61.4 (10.5)	8/12	20 (100%)	ND
JA Chang et al., 1999 [102]	Cross-sectional	ILD	60.5 [29-81]	22/28	50 (100%)	ND
MI Feltrim et al., 2008 [121]	Cross-sectional	pre-LTx	54 (11)	3/4	7 (100%)	Assessor applied
**WHO functional class**						
C Kronberger et al., 2023 [16]	Cross-sectional	PAH	66 (15)	66/40	106 (100%)	ND

First Author (ref)	Design of study	Disease	Age	Sex (F/M)	Complete n (% of initial)	Protocol
M Alotaibi et al., 2023 [140]	Longitudinal Retrospective	PAH	51.9 (18.4)	663/201	864 (100%)	ND
C Keen et al., 2023 [15]	Longitudinal, Prospective	PAH	53.9 (14.9)	47/13	60 (100%)	ND
M Okamura et al., 2022 [27]	Cross-sectional	PAH	50.7 (16.2)	15/7	22 (100%)	ND
L Matura et al., 2022 [141]	Cross-sectional	PAH	50.6 (17.8)	60/0	60 (100%)	
A Arvanitaki et al., 2020 [142]	Cross-sectional	PAH	57.9 (16.3)	22/12	34 (100%)	ND
AS Babu et al., 2019[c] [105]	Interventional	PAH	51.4 (13.7)	20/22	34 (80%)	ND
K Karauzum et al., 2019 [143]	Cross-sectional	PAH	54.9 (10.4)	14/4	18 (100%)	Obtained from clinical history
M Aldemir et al., et al., 2018 [144]	Cross-sectional	PAH	61 (10)	23/15	38 (100%)	ND
C Mihai et al., et al., 2017 [145]	Cross-sectional	PAH	61 (54-66)	14/11	25 (100%)	ND
M Waligora et al., 2017 [146]	Longitudinal, Prospective	PAH	50.0 (15.7)	47/19	66 (100%)	ND
SA Segrera et al., 2017 [147]	Interventional	PAH	58.6 (9.9)	14/8	22 (73%)	ND
N Tanabe et al., 2017[c] [148]	Interventional	PAH	44.5 (13.3)	26/11	33 (89%)	ND
SK Saha et al., 2016 [149]	Cross-sectional	PAH	64 (10)	12/13	25 (100%)	Obtained from clinical history
M Kukkonen et al., 2016 [106]	Cross-sectional	PAH	53 (16.2)	46/32	78 (100%)	"…estimated based on a question on the SF-36 form (how much a moderately strenuous activity e.g. brisk walking on level ground is restricted by health issues)."
L Godinas et al., 2016 [150]	Cross-sectional	PAH	48 (15)	101/52	153 (100%)	ND
SC Mathai et al., 2015[c] [107]	Interventional	PAH	55 (15)	317/88	405 (100%)	ND
LJ Rubin et al., 2015[c] [151]	Interventional	PAH	50 (16)	317/79	339 (85%)	ND
AE Frost et al., 2015 [152]	Interventional	PAH	50 [18-27]	83/20	103 (100%)	ND

First Author (ref)	Design of study	Disease	Age	Sex (F/M)	Complete n (% of initial)	Protocol
DJ Webb et al., 2015 [153]	Interventional	PAH	48 (15)	56/15	71 (100%)	ND
ID Laoutaris et al., 2015 [108]	Interventional	PAH	48.6 (12.7)	4/1	5 (100%)	ND
Y Zhuang et al., 2014 [154]	Interventional	PAH	52 (12)	46/14	54 (90%)	ND
SA Mouratoglou et al., 2014 [155]	Longitudinal, Prospective	PAH	49 (15)	16/6	22 (100%)	ND
LA Matura et al., 2014 [109]	Cross-sectional	PAH	53.5 (15.1)	127/22	149 (100%)	"…was defined from assessment of self-reported symptom severity and activity limitations by the principal investigator."
HA Ghofrani et al., 2013 [156]	Interventional	PAH	51 (17)	203/34	237 (93%)	ND
LA Matura et al., 2012 [110]	Cross-sectional	PAH	52.2 (16.0)	76/17	93	"…was defined from assessment of self-reported symptom severity and activity limitations by the principal investigator."
RJ Oudiz et al., 2012 [157]	Interventional	PAH	53 (16)	48/15	52 (82)	ND
R Condliffe et al., 2009 [158]	Longitudinal[d]	PAH	63.9 (10.5)	212/46	259 (100%)	ND
N Galie et al., 2009 [159]	Interventional	PAH	53 (15)	59/20	79 (100%)	ND
C Gilbert et al., 2009 [112]	Interventional	PAH	ND	ND	278	ND
N Galie et al., 2008 [114]	Interventional	PAH	52 (15)	48/16	64 (100%)	ND
J White et al., 2006 [117]	Cross-sectional	PAH	48.6 (11.8)	23/4	27	ND
N Galie et al., 2005 [160]	Interventional	PAH	51 (16)	54/10	64 (100%)	ND
N Galie et al., 2005 [161]	Interventional	PAH	48 (15)	56/15	71 (100%)	ND
NYHA functional class						
DK Ozen et al., 2020 [162]	Cross-sectional	ILD	52.5 (10.7)	46/10	56 (93%)	ND
S Witt et al., 2019 [85]	Cross-sectional	ILD	67.3 (10.7)	69/189	258 (100%)	Assessor applied
ZÇ Sozener et al., 2010 [139]	Cross-sectional	ILD	52.7 (9.8)	28/5	33 (100%)	ND

First Author (ref)	Design of study	Disease	Age	Sex (F/M)	Complete n (% of initial)	Protocol
Rusanov et al., 2008 [53]	Cross-sectional	ILD	58 (11)	22/29	51 (100%)	ND
M Pereira et al., 2022 [17]	Cross-sectional	PAH	44.3 (13.2)	4/16	20 (100%)	ND
S Unlu et al., 2022 [163]	Longitudinal, Retrospective	PAH	58.8 (14.2)	20/5	25 (100%)	ND
K Bunclark et al., 2021 [164]	Cross-sectional	PAH	54.4 (16.4)	64/65	129 (70%)	ND
EV Karelkina et al., 2020[c] [165]	Interventional	PAH	48 (15)	16/3	14 (73%)	ND
B Kahraman et al., 2020 [36]	Cross-sectional	PAH	50.2 (18.0)	7/31	38 (100%)	ND
T Naal et al., 2018 [166]	Longitudinal, Retrospective	PAH	51.3 (18.1)	203/74	277 (95%)	ND
E Ozpelit et al., 2015 [167]	Longitudinal, Prospective	PAH	56 [18-77]	26/7	33 (100%)	ND
N Chueamuangphan et al., 2014 [168]	Interventional	PAH	36.1 (14.6)	12/4	16 (100%)	ND
N Malik et al., 2012 [169]	Cross-sectional	PAH	49 (11)	27/5	32 (100%)	ND
RL Benza et al., 2011[c] [170]	Longitudinal[d]	PAH	54 [18-75]	167/39	122 (59%)	ND
L Tokgozoglu et al., 2009 [171]	Longitudinal, Prospective	PAH	45.4 (9.7)	32/19	51 (100%)	ND
OA Minai et al., 2007 [172]	Cross-sectional	PAH	51 (12)	23/7	30 (100%)	Obtained from clinical history
EM Chau et al., 2007 [173]	Interventional	PAH	41 (7)	5/1	6 (100%)	ND
N Zafrir et al., 2007 [174]	Cross-sectional	PAH	50.8 (14.7)	22/7	29 (100%)	ND
R Souza et al., 2007 [175]	Cross-sectional	PAH	37 (2)	32/10	42 (100%)	ND
VV McLaughlin et al., 2006[c] [176]	Interventional	PAH	51 (14)	27/7	30 (88%)	ND
E Cenedese et al., 2006 [177]	Interventional	PAH	50 [46-54]	31/17	48 (100%)	Applied using a standardized questionnaire
J Shen et al., 2005 [178]	Interventional	PAH	53 (18)	20/7	27 (100%)	ND
DB Badesch et al., 2000 [179]	Interventional	PAH	53 (13.1)	51/5	56 (100%)	ND

First Author (ref)	Design of study	Disease	Age	Sex (F/M)	Complete n (% of initial)	Protocol
M Yigla et al., 1997 [180]	Longitudinal, Retrospective	PAH	70.5 (6.7)	10/4	14 (100%)	ND
**AQLQ – Activity limitation**						
E Calik-Kutukcu et al., 2022 [181]	Cross-sectional	Asthma	43.4 (14.7)	ND	34 (100%)	Self-administered
A Freeman et al., 2021 [182]	Interventional	Asthma	31.3 (10.0)	6/0	6 (86%)	Self-administered
SM Lage et al., 2021 [183]	Interventional	Asthma	40.2 (13.4)	14/6	20 (58%)	Assessor applied
KB Evaristo et al., 2021 [184]	Interventional	Asthma	50.6 (9.2)	17/8	25 (100%)	ND
S Majd et al., 2020 [185]	Interventional	Asthma	58 (11.0)	18/12	30 (49%)	ND
PD Freitas et al., 2017 [186]	Interventional	Asthma	48.5 (9.6)	25/0	25 (92%)	ND
A Meyer et al., 2015 [56]	Interventional	Asthma	54.0 (11.0)	5/8	13 (92%)	Self-administered
A Refaat et al., 2015 [187]	Interventional	Asthma	35.8 (1.7)	21/17	38 (100%)	Self-administered
S Pakhale et al., 2015 [188]	Interventional	Asthma	43.3 (10.3)	15/1	15 (93%)	ND
TZ Rondinel et al., 2015 [189]	Interventional	Asthma	57 [37-51]	6/2	8 (73%)	ND
S Turner et al., 2011 [60]	Interventional	Asthma	65.8 (10.8)	11/8	19 (95%)	Self-administered
B Kligler et al., 2011 [61]	Interventional	Asthma	43.4 (11.8)	61/16	77 (100%)	ND
R Vempati et al., 2009 [190]	Interventional	Asthma	33.5 (11.4)	16/13	29 (100%)	Self-administered
J-S Choi et al., 2005 [191]	Interventional	Asthma	46.2 (14.7)	17/23	40 (100%)	Doubtful
G Riccioni et al., 2002 [192]	Interventional	Asthma	26.9 (12.3)	7/8	15 (100%)	Assessor applied
W Busse et al., 1998 [193]	Interventional	Asthma	37.2 [12-80]	129/134	263 (100%)	Self-administered
T Van der Molen et al., 1998 [194]	Interventional	Asthma	40.8 (13.2)	28/28	56 (100%)	Assessor applied
J Ware et al., 1998 [69]	Interventional	Asthma	39.5	89/57	146 (82%)	Self-administered
T Van der Molen et al., 1997 [71]	Cross-sectional	Asthma	44 (14)	56/54	110 (100%)	Self-administered
E Juniper et al., 1994 [195]	Longitudinal[d]	Asthma	42 (13.7)	24/15	39 (100%)	Assessor applied
E Juniper et al., 1993 [196]	Longitudinal[d]	Asthma	42 (13.7)	24/15	37 (95%)	Assessor applied

First Author (ref)	Design of study	Disease	Age	Sex (F/M)	Complete n (% of initial)	Protocol
**CFQoL – Physical Functioning**						
KB Knudsen et al., 2017 [197]	Interventional	Cystic Fibrosis	23.6 [18-30]	12/6	18 (90%)	Self-administered
KCA Aguiar et al., 2017 [198]	Cross-sectional	Cystic Fibrosis	ND	ND	52 (100%)	Self-administered
VJ Ribeiro-Moço et al., 2017 [199]	Cross-sectional	Cystic Fibrosis	25 (6)	9/12	21 (70%)	ND
T Radtke et al., 2016 [4]	Interventional	Cystic Fibrosis	29 [25-36]	8/6	14 (100%)	ND
JT Penafortes et al., 2014 [200]	Cross-sectional	Cystic Fibrosis	24.5 [22-34]	6/8	14 (47%)	Self-administered
EJ Dill et al., 2013 [201]	Longitudinal, Prospective	Cystic Fibrosis	32.5 (10.6)	153/125	278 (92%)	ND
L Kelemen et al., 2012 [202]	Cross-sectional	Cystic Fibrosis	29.4 (8.5)	42/35	73 (95%)	Self-administered
CA Sandsund et al., 2011 [203]	Interventional	Cystic Fibrosis	27 [25-32]	5/5	10 (100%)	Self-administered
AC Young et al., 2008 [204]	Cross-sectional	Cystic Fibrosis	37 (8)	2/6	8 (89%)	ND
L Gee et al., 2000 [77]	Interventional	Cystic Fibrosis	27 [16-53]	15/17	32 (100%)	Self-administered
SF-12 – Physical Functioning or Physical Component Summary						
WT Liu et al., 2011 [205]	Interventional	Asthma	50.4 (1.9)	22/22	43 (72%)	ND
B Kligler et al., 2011 [61]	Interventional	Asthma	45.7 (9.5)	64/13	77 (100%)	ND
AA Perez et al., 2020 [47]	Long. Prospective	post-LTx	31 (7.9)	12/15	27 (90%)	ND
PROMIS-29 – Physical Functioning						
CJ Fisher et al., 2019 [89]	Cross-sectional	ILD	51.9 (11.8)	59/14	73 (100%)	Assessor applied
SE Yount et al., 2016 [206]	Cross-sectional	ILD	61 (5.6)	155/65	220 (100%)	ND
ME Hinchcliff et al., 2015 [92]	Cross-sectional	ILD	52 [27-71]	90/10	100 (100%)	Self-administered
Living with Asthma Questionnaire (LWAQ) – Physical Health Construct						
Y Tohda et al., 2006 [63]	Interventional	Asthma	57.9 (15.3)	16/28	44 (100%)	ND
T Van der Molen et al., 1998 [194]	Interventional	Asthma	40.8 (13.2)	28/28	56 (100%)	Assessor applied
T Van der Molen et al., 1997 [71]	Cross-sectional	Asthma	44 (14)	56/54	110 (100%)	Self-administered

First Author (ref)	Design of study	Disease	Age	Sex (F/M)	Complete n (% of initial)	Protocol
Asthma Quality of Life from EPM- Physical Limitation						
HJ Pai et al., 2015 [57]	Interventional	Asthma	32.5	ND	31 (100%)	Self-administered
FA Mendes et al., 2013 [207]	Cross-sectional	Asthma	34.9 (8.2)	17/4	21 (100%)	ND
MA De Oliveira et al., 2005 [65]	Longitudinal, Prospective	Asthma	28.2 (11.3)	19/6	35 (100%)	Self-administered
Cambridge Pulmonary Hypertension Outcome Review (CAMPHOR) – ACTIVITY						
K Bunclark et al., 2021 [164]	Cross-sectional	PAH	54.4 (16.4)	64/65	129 (70%)	ND
T Koudstaal et al., 2019 [208]	Longitudinal, Prospective	PAH	45.1 (15.5)	13/8	21 (100%)	Self-administered
LA Matura et al., 2012 [110]	Cross-sectional	PAH	52.2 (16.0)	76/17	93	ND
London – ADL – Physical Activity Score						
E Calik-Kutukcu et al., 2022 [181]	Cross-sectional	Asthma	43.4 (14.7)	ND	34 (100%)	ND
N Duruturk et al., 2018 [122]	Interventional	Asthma	46.5 (13.3)	14/6	16 (80%)	ND
GP Reinaldo et al., 2022 [50]	Cross-sectional	ILD	60.7 (10.7)	19/8	27 (100%)	Self-administered
QoL – Bronchiectasis – Physical Functioning						
A Lee et al., 2022 [209]	Cross-sectional	Bronchiectasis	58 (18)	39/21	60 (100%)	ND
A José et al., 2021[c] [210]	Interventional	Bronchiectasis	44.4 (16.1)	16/17	27 (82%)	ND
CO De Camargo et al., 2020 [124]	Cross-sectional	Bronchiectasis	48 (14.1)	61/57	108 (125%)	Assessor applied
M-AQLQ – Activity Limitation						
G Georga et al., 2019 [211]	Interventional	Asthma	49.4 (13)	16/7	21 (91%)	ND
J Ma et al., 2017 [212]	Interventional	Asthma	52.2 (11.9)	34/12	44 (95%)	Self-administered
Minessota living with heart failure questionnaire – Physical Sub score						
GK Aslan et al., 2020 [213]	Interventional	PAH	47.2 (13.2)	13/12	20 (100%)	ND
E Cenedese et al., 2006 [177]	Interventional	PAH	50 [46-54]	31/17	48 (100%)	Self-administered

First Author (ref)	Design of study	Disease	Age	Sex (F/M)	Complete n (% of initial)	Protocol
Functional performance inventory						
FX McCormack et al., 2011 [214]	Interventional	ILD	45 (10.9)	ND	46 (100%)	ND
Seattle Obstructive Lung Disease Questionnaire SOLQ – Physical function score						
E Bulcun et al., 2015 [73]	Cross-sectional	Bronchiectasis	48.1 (13.5)	46/32	78 (100%)	ND
Eastern Cooperative Oncology Performance Status						
K Oishi et al., 2022 [8]	Cross-sectional	ILD	72 [64-78]	52/62	116 (100%)	Assessor applied
PROMIS Physical Function – Short Form 8a						
L Matura et al., 2022 [141]	Cross-sectional	PAH	50.6 (17.8)	60/0	60 (100%)	Self-administered
Multi-Dimensional Health Assessment Questionnaire – Physical function						
JJ Swigris et al., 2010 [215]	Interventional	ILD	52.8 (19.7)	35/13	48 (100%)	Self-administered
Health Assessment Questionnaire – Disability Index						
N Mena-Vàzquez et al., 2023 [216]	Longitudinal, Prospective	ILD	63.9 (8.3)	88/22	110 (100%)	ND

Interventional studies refer to randomized and non-randomized clinical trials. **Abbreviations:** ILD – Interstitial lung disease; PAH – Pulmonary arterial hypertension; pre-LTx – patients with chronic respiratory disease waiting to lung transplantation; post-LTx – patients with chronic respiratory disease who underwent to lung transplantation; ND – no description; 1min-STS – 1minute sit-to-stand; 4MGS – four-metre gait speed; 30sec-STS – 30 seconds sit-to-stand; 5rep-STS – 5 repetitions sit-to-stand; SPPB – short physical performance battery; TUG – timed-up-and-go; 8-FUGT – 8-foot-up-and-go; Glitre ADL – Glittre Activities of daily living; CS-PFP – continuous scale physical function performance test; 3min-STS – 3-minute sit-to-stand; 15-stepsC – 15-steps climbing; PPT – Physical performance test; SF-36 – Medical Outcomes Study 36-item Short Form of Health Survey; SGRQ – Saint´s George Respiratory Questionnaire; WHOfc – World Health Organization functional class; NYHA – New York heart association; AQLQ – Asthma Quality of Life Questionnaire; CFQoL – Cystic fibrosis quality of life; SF-12 – Medical Outcomes Study 12-item Short Form of Health Survey; Promis-29 – Patient-Reported Outcomes Measurement Information System; LWAQ – Living with Asthma Questionnaire; M-AQLQ – Mini Asthma Quality of Life Questionnaire; CAMPHOR – Cambridge pulmonary hypertension outcome review; London ADL – London Activities of Daily Living; QoL-EPM – Asthma Quality of Life from Escola Paulista de Medicina; FPI – Functional performance inventory; MLHFQ – Minessota living with heart failure questionnaire; QoL-B – Quality of Life in Bronchiectasis; SOLQ – Seattle Obstructive Lung Questionnaire; ECOPs – Eastern Cooperative Oncology Performance Status; PROMIS – PF – Patient-Reported Outcomes Measurement Information System Physical Function Short Form 8a; MDHAQ – Multi dimensional health assessment questionnaire; HAQ-DI – Health Assessment Questionnaire Disability Index.

[a]Average results from men and women combined

[b]Results from pooled data of both asbestosis and silicosis.

[c]Number of men and women from the included participants, without attrition.

[d]Did not report the type of cohort (retrospective or prospective).

Table S3. Reason for exclusion after full text assessment and data extraction process.

References	Reason for exclusion
Akdoğan, A., et al. Eur J Rheumatol, 2015	Instrument of interest used to separate groups
Alison, J. Australian journal of physiotherapy, 2000	Without access
Almeida, V. P., et al. Clinics (Sao Paulo), 2013	Domain or result of functional performance was not described
Amin, A., et al. Research in Cardiovascular Medicine, 2015	Inclusion/exclusion criteria was based on score of instruments
Andreassen, A. K., et al. Tidsskrift for den Norske Laegeforening, 2003	Inclusion/exclusion criteria was based on score of instruments
Andreassen, A. K., et al. Tidsskr Nor Laegeforen 2003	Inclusion/exclusion criteria was based on score of instruments
Andreasson, K. H., et al. Annals of the American Thoracic Society, 2022	Domain or result of functional performance was not described
Ar, U., et al. Exp Clin Transplant, 2022	Results with mixed disease or age groups
Araújo, A. S., et al. Lung, 2022	Domain or result of functional performance was not described
Areias, V., et al. Rev Port Pneumol, 2012	Results with mixed disease or age groups
Arikan, H., et al. Res Dev Disabil, 2015	Results with mixed disease or age groups
Artem Ovchinnikov, A. G., et al. European Journal of Heart Failure, 2018	Inclusion/exclusion criteria was based on score of instruments
Badesch, D. B., et al. Current therapeutic research - clinical and experimental, 2002	Inclusion/exclusion criteria was based on score of instruments
Barst, R. J., et al. Journal of the American College of Cardiology, 2006	Inclusion/exclusion criteria was based on score of instruments
Bauer, R., et al. Respir Med, 2007	Inclusion/exclusion criteria was based on score of instruments
Berman-Rosenzweig, E., et al. Pulmonary circulation, 2014	Results with mixed disease or age groups
Bhougal, B. N., et al. K Practitioner, 2017	Inclusion/exclusion criteria was based on score of instruments
Bruce, R. A., et al. Proc Soc Exp Biol Med, 1950	Without access
Bussotti, M., et al. Cardiovascular and Hematological Disorders - Drug Targets, 2017	Inclusion/exclusion criteria was based on score of instruments
Byrd, R., et al. Cardiopulmonary Physical Therapy Journal, 2022	Results with mixed disease or age groups
Carlsen, K. H. Tidsskr Nor Laegeforen, 2000	Without access
Cascino, T., et al. Journal of the American College of Cardiology, 2019	Domain or result of functional performance was not described
Chan, L., et al. Chest 2013	Inclusion/exclusion criteria was based on score of instruments

Chang, W. Y., et al. The european respiratory journal, 2014	Domain or result of functional performance was not described
Channick, R. N., et al. Journal of the american college of cardiology, 2006	Inclusion/exclusion criteria was based on score of instruments
Cho, P. S. P., et al. Lung., 2019	Domain or result of functional performance was not described
Choi, J., et al. Int J Telerehabil, 2016	Results with mixed disease or age groups
Clavé, M. M., et al. Congenital heart disease 2019	Inclusion/exclusion criteria was based on score of instruments
Codina, P., et al. ESC Heart Fail, 2022	Inclusion/exclusion criteria was based on score of instruments
Courtwright, A. M., et al. Transplantation, 2018	Results with mixed disease or age groups
Courtwright, A. M., et al. Clin Transplant	Results with mixed disease or age groups
da Costa Junior, A. A., et al. International Journal of Cardiovascular Imaging, 2017	Inclusion/exclusion criteria was based on score of instruments
Dalton, T., et al. Exp Gerontol, 2022	Without access
de Jong, W., et al. Chest, 1994	Results with mixed disease or age groups
Del Pozo, R., et al. Int J Cardiol, 2019	Inclusion/exclusion criteria was based on score of instruments
Denton, C. P., et al. Annals of the rheumatic diseases, 2006	Inclusion/exclusion criteria was based on score of instruments
Deucher, R. A. O., et al.	Without access
Develi, E., et al. Physiother Res Int, 2021	Without access
Diamond, J. M., et al. Clin Transplant	Without access
Dziedziczko, A., et al. Pneumonol Pol	Without access
Ebenbichler, G. R., et al. Wien Klin Wochenschr	Results with mixed disease or age groups
Eber, E., et al. Journal of cystic fibrosis	Results with mixed disease or age groups
Ehlken, N., et al. European heart journal	Results with mixed disease or age groups
Elganady, A., et al. Egyptian Journal of Chest Diseases and Tuberculosis	Domain or result of functional performance was not described
Erkılınç, A., et al. Turk Gogus Kalp Damar Cerrahisi Derg	Results with mixed disease or age groups
Estenne, M. and Higenbottam, T. W. European Respiratory Monograph, 1997	Without access
Farha, S., et al. JCI insight, 2017	Inclusion/exclusion criteria was based on score of instruments
Feltrim, M. I., et al. Transplant Proc, 2014	Results with mixed disease or age groups
Filusch, A., et al. Int J Cardiol, 2011	Results with mixed disease or age groups
Filusch, A., et al. Clin Res Cardiol, 2011	Instrument of interest used to separate groups
Florian, J., et al. J Bras Pneumol, 2013	Instrument of interest used to separate groups
Flume, P. A., et al. Journal of cystic fibrosis, 2019	Results with mixed disease or age groups
Franchi, S. M., et al. Arq Bras Cardiol, 2010	Without access

Fuller, L. M., et al. Archives of physical medicine and rehabilitation, 2017	Results with mixed disease or age groups
Gaddie, J., et al. British Journal of Diseases of the Chest, 1972	Without access
Galiè, N., et al. Journal of the American College of Cardiology, 2002	Inclusion/exclusion criteria was based on score of instruments
Garg, N., et al. International Journal of Cardiology, 2007	Inclusion/exclusion criteria was based on score of instruments
Genao, L., et al. Am J Transplant, 2013	Results with mixed disease or age groups
Gerhardt, F., et al. Heart, 2017	Inclusion/exclusion criteria was based on score of instruments
Gruet, M., et al. Science and Sports, 2013	Without access
Guan, W. J., et al. Chest, 2023	Domain or result of functional performance was not described
Haber, P. Praxis und Klinik der Pneumologie, 1988	Without access
Halank, M., et al. Respir Med, 2017	Inclusion/exclusion criteria was based on score of instruments
Han, B., et al. Aging & Mental Health, 2013	Results with mixed disease or age groups
Hardy, S., et al. Respir Res, 2022	Results with mixed disease or age groups
Hebestreit, H., et al. BMC Pulm Med, 2014	Results with mixed disease or age groups
Hemnes, A. R., et al. Chest, 2021	Inclusion/exclusion criteria was based on score of instruments
Hiremath, J., et al. Journal of heart and lung transplantation, 2010	Inclusion/exclusion criteria was based on score of instruments
Hoffman, M., et al. Physiother Theory Pract, 2021	Results with mixed disease or age groups
Hoy, H. M., et al. Prog Transplant, 2012	Results with mixed disease or age groups
Hu, L., et al. Clinical transplantation, 2017	Results with mixed disease or age groups
Huo, Y., et al. BMC cardiovascular disorders, 2016	Inclusion/exclusion criteria was based on score of instruments
Ihle, F., et al. Journal of Heart and Lung Transplantation, 2011	Results with mixed disease or age groups
Irisawa, H., et al. Circulation Journal, 2014	Inclusion/exclusion criteria was based on score of instruments
Jansa, P., et al. Vnitrni Lekarstvi, 2007	Without access
Jeremie, U., et al. Arbeitsmedizin Sozialmedizin Umweltmedizin, 2006	Without access
Jiménez López-Guarch, C., et al. Rev Esp Cardiol, 2004	Inclusion/exclusion criteria was based on score of instruments
José, A. and Dal Corso, S. Braz J Phys Ther, 2015.	Results with mixed disease or age groups
Kanorskiĭ, S. G., et al. Kardiologiia, 2012	Inclusion/exclusion criteria was based on score of instruments
Kato, D., et al. J Phys Ther Sci, 2017	Results with mixed disease or age groups
Kerschan-Schindl, K., et al. Clin Exp Med, 2019	Results with mixed disease or age groups
Kohlbrenner, D., et al. Repir Carem, 2020.	Results with mixed disease or age groups

Krivoruk, V. I. Vopr Kurortol Fizioter Lech Fiz Kult, 1975	Without access
Langer, D., et al. Am J Transplant, 2012	Results with mixed disease or age groups
Lanuza, D. M., et al. Chest, 2000	Results with mixed disease or age groups
Lindelof, B., et al. Tijdschrift van het Instituut voor Mijnhygiene, 1974	Without access
Lingner, H., et al. J Asthma, 2015	Domain or result of functional performance was not described
Loh, B., et al. Padiatrische Praxis, 2017	Without access
Lopes, A.J., et al. J. Phys. Ther. Sci., 2014	Functional tests were used to assess balance
Marsico, A., et al. European journal of physical and rehabilitation medicine, 2021	Inclusion/exclusion criteria was based on score of instruments
Mathur, S., et al. Cardiopulmonary Physical Therapy Journal, 2017	Results with mixed disease or age groups
Mayer, K. P., et al. Transplant Proc, 2021	Results with mixed disease or age groups
McLaughlin, V. V., et al. Journal of the American College of Cardiology, 2010	Inclusion/exclusion criteria was based on score of instruments
Mensink-Bout, S. M., et al. Pediatr Allergy Immunol, 2022	Results with mixed disease or age groups
Meyer, F. J., et al. Eur Respir J, 2005	Inclusion/exclusion criteria was based on score of instruments
Minhas, J., et al. Annals of the American Thoracic Society, 2022	Inclusion/exclusion criteria was based on score of instruments
Miozzo, A. P., et al. JMIR Rehabil Assist Technol, 2022	Results with mixed disease or age groups
Mue, S., et al. Journal of pharmacotherapy, 1979	Without access
Na, J. O., et al. Monaldi Archives for Chest Disease - Pulmonary Series, 2005	Results with mixed disease or age groups
Naderi, N., et al. Res Cardiovasc Med, 2014	Inclusion/exclusion criteria was based on score of instruments
Nathan, S. D., et al. The Lancet. Respiratory medicine, 2019	Inclusion/exclusion criteria was based on score of instruments
Nathan, S. D., et al. Respiratory medicine, 2015	Domain or result of functional performance was not described
Olschewski, H., et al. New England journal of medicine, 2002	Inclusion/exclusion criteria was based on score of instruments
Paula-Ribeiro, M., et al. J Appl Physiol (1985), 2021	Inclusion/exclusion criteria was based on score of instruments
Pehlivan, E., et al. Exp Clin Transplant, 2018	Results with mixed disease or age groups
Pehlivan, E., et al. Turk Thorac J, 2020	Results with mixed disease or age groups
Penafortes J.T.S. et al. Braz J Phys Ther., 2013	Functional tests were used to assess balance
Perna, E. R., et al. Revista de la Federacion Argentina de Cardiologia, 2021	Inclusion/exclusion criteria was based on score of instruments
Purokivi, M., et al. Duodecim, 2014	Without access
Quittner, A., et al. Health and quality of life outcomes, 2015	Results with mixed disease or age groups

Rakhmawati, A., et al. Ann Rehabil Med, 2020	Inclusion/exclusion criteria was based on score of instruments
Retsch-Bogart, G. Z., et al. Chest, 2009	Results with mixed disease or age groups
Ricotti, S., et al. Monaldi Arch Chest Dis, 2017	Results with mixed disease or age groups
Rose, J. A., et al. Chest, 2016	Inclusion/exclusion criteria was based on score of instruments
Rozenberg, D., et al. J Heart Lung Transplant, 2018	Results with mixed disease or age groups
Rozenberg, D., et al. Transplantation, 2017	Results with mixed disease or age groups
Rubenfire, M., et al. Chest, 2007	Inclusion/exclusion criteria was based on score of instruments
Rubin, L. J., et al. New England journal of medicine, 2002	Inclusion/exclusion criteria was based on score of instruments
Rutherford, R. M., et al. Am J Transplant, 2005	Results with mixed disease or age groups
Saglam, M., et al. Journal of Cardiopulmonary Rehabilitation and Prevention, 2015	Inclusion/exclusion criteria was based on score of instruments
Saglam, M., et al. J Phys Ther Sci, 2015	Inclusion/exclusion criteria was based on score of instruments
Sahlberg, M. E., et al. Chest, 2005	Results with mixed disease or age groups
Sartori, R., et al. Journal of Cystic Fibrosis, 2008	Results with mixed disease or age groups
Sasayama, S., et al. Circulation Journal, 2005	Inclusion/exclusion criteria was based on score of instruments
Satlam, M., et al. Fizyoterapi Rehabilitasyon, 2014	Inclusion/exclusion criteria was based on score of instruments
Sawyer, A., et al. J Clin Med, 2020	Domain or result of functional performance was not described
Schaeffer, M. R., et al. Respir Med, 2022	Without access
Scott, H. A., et al. Clinical and experimental allergy, 2013	Domain or result of functional performance was not described
Shang, X., et al. Oncotarget, 2017	Inclusion/exclusion criteria was based on score of instruments
Shiner, C. T., et al. Pm r, 2019	Results with mixed disease or age groups
Singer, J. P., et al. Thorax, 2014	Results with mixed disease or age groups
Singer, J. P., et al. Am J Transplant, 2023	Without access
Singer, J. P., et al. J Heart Lung Transplant, 2023	Results with mixed disease or age groups
Singer, J. P., et al. Am J Transplant, 2018	Instrument of interest used to separate groups
Singer, J. P., et al. Am J Respir Crit Care Med, 2015	Instrument of interest used to separate groups
Sitbon, O., et al. Eur Respir J, 2020	Inclusion/exclusion criteria was based on score of instruments
Snipelisky, D., et al. Clinical Cardiology, 2018	Inclusion/exclusion criteria was based on score of instruments

Sohn, D. W., et al. Korean Circulation Journal, 2009	Inclusion/exclusion criteria was based on score of instruments
Song, J. H., et al. Acute Crit Care, 2018	Results with mixed disease or age groups
Speich, R., et al. Schweiz Med Wochenschr, 2000	Without access
Swaminathan, A. C., et al. Chest, 2023	Results with mixed disease or age groups
Taçoy, G., et al. West Indian Med J, 2015	Without access
Tahara, N., et al. Circulation Journal, 2016	Results with mixed disease or age groups
Tarrant, B. J., et al. Phys Ther, 2020	Results with mixed disease or age groups
Trevor, J. L., et al. J Asthma, 2015	Domain or result of functional performance was not described
Utsunomiya, A., et al. Modern Rheumatology, 2021	Results with mixed disease or age groups
Vainshelboim, B., et al. Eur J Phys Rehabil Med, 2017	Domain or result of functional performance was not described
Venado, A., et al. Thorax, 2020	Results with mixed disease or age groups
Venado, A., et al. J Heart Lung Transplant, 2019	Results with mixed disease or age groups
Vetter, C., Deutsches Arzteblatt International, 2021	Without access
Watson, K., et al. J Physiother, 2023	Results with mixed disease or age groups
Weinreich, U. M., et al. Chronic respiratory disease, 2022	Domain or result of functional performance was not described
Wichman, T. O., et al. Advances in Redox Research, 2022	Inclusion/exclusion criteria was based on score of instruments
Wickerson, L., et al. Clin Transplant, 2020	Results with mixed disease or age groups
Wickerson, L., et al. Journal of Heart and Lung Transplantation, 2020	Results with mixed disease or age groups
Wickerson, L., et al. J Cardiopulm Rehabil Prev, 2023	Without access
Wilkins, M. R., et al. American journal of respiratory and critical care medicine, 2005	Inclusion/exclusion criteria was based on score of instruments
Xu, X. Q., et al. Zhonghua xin xue guan bing za zhi, 2010	Without access
Yuen, H. K., et al. Journal of Cardiopulmonary Rehabilitation and Prevention, 2019	Domain or result of functional performance was not described

Table S4. Characteristics of the participants stratified by disease in all studies and in the studies investigating measurement properties.

Disease	Studies	n	Age Mean / [CI95]	Sex (F/M) (%)	VEF1 (%pred)	FVC (%pred)	FEV1/FVC
**All studies (n=184)**							
Asthma	49	4076	46.3 [39-52]	58/42	73	78	68
Bronchiectasis	14	641	53.9 [48-58]	51/49	63	77	69
Cystic Fibrosis	16	836	28.6 [25-32]	51/49	64	80	61
ILD	70	4790	62.8 [57-70]	41/59	74	73	76
PAH	67	5362	51.5 [48-53]	72/28	Ɵ	Ɵ	Ɵ
pre-LTx	2	52	57.0 [54-60]	22/30	55	50	Ɵ
post-LTx	2	54	41.0 [31-51]	27/27	54	61	Ɵ
**Studies investigating measurement properties (n=27)**							
Asthma	6	268	40.9 [42-44]	60/40	83	Ɵ	78
Bronchiectasis	3	297	49.5 [48-52]	55/45	66	88	76
Cystic Fibrosis	4	76	29.7 [28-31]	48/52	58	74	Ɵ
ILD	12	1020	62.9 [59-69]	48/52	76	70	85
PAH	3	192	55.4 [50-66]	45/55	Ɵ	Ɵ	Ɵ
pre-LTx	0	Ɵ	Ɵ	Ɵ	Ɵ	Ɵ	Ɵ
post-LTx	0	Ɵ	Ɵ	Ɵ	Ɵ	Ɵ	Ɵ

Abbreviations: ILD – Interstitial lung disease; PAH – Pulmonary arterial hypertension; pre-LTx – patients with chronic respiratory disease waiting to lung transplantation; post-LTx – patients with chronic respiratory disease who underwent to lung transplantation; SD – Standard deviation; CI – confidence interval; FEV1 – forced expiratory volume in first second; % of predicted; FVC – forced vital capacity; % of predicted; Ɵ – Missing information.

Table S5. Methodological quality of included studies assessed by Cosmin checklist.

Author, year (ref)	PROM Development	Internal Consistency	Cross-cultural validity	Reliability	Measurement Error	Criterion Validity	Construct Validity	Responsiveness
T Van der Molen,1997 [71]	N/A	Very Good	N/A	N/A	N/A	Very Good	N/A	N/A
T Radtke, 2016 [4]	N/A	N/A	N/A	Adequate	N/A	Very Good	N/A	Very Good
T Radtke, 2017 [3]	N/A	N/A	N/A	N/A	N/A	Very Good	N/A	N/A
CM Nolan,2018 [42]	N/A	N/A	N/A	Adequate	Adequate	Very Good	N/A	Very Good
ME Hinchcliff, 2015 [92]	N/A	N/A	N/A	N/A	N/A	Very Good	N/A	N/A
AL Olson, 2015 [52]	N/A	Very Good	N/A	Adequate	N/A	Very Good	N/A	N/A
SE Yount, 2016 [206]	N/A	N/A	N/A	Doubtful	N/A	N/A	N/A	N/A
S Witt, 2019 [85]	N/A	Very Good	N/A	Doubtful	N/A	Very Good	N/A	Very Good
AEM Bloem,2018 [25]	N/A	N/A	N/A	Doubtful	N/A	Very Good	N/A	N/A
JM Oliveira, 2020 [1]	N/A	N/A	N/A	Very Good	Very Good	Very Good	N/A	N/A
R Hirabayashi, 2020 [40]	N/A	N/A	N/A	N/A	N/A	Very Good	N/A	N/A
PF Tremblay Labrecque, 2020 [12]	N/A	N/A	N/A	Doubtful	Doubtful	Very Good	N/A	N/A
B Kahraman, 2020 [36]	N/A	N/A	N/A	Doubtful	Doubtful	Very Good	N/A	N/A
CL Zamboti, 2021 [10]	N/A	N/A	N/A	Very Good	Very Good	Very Good	N/A	N/A
A Fedi, 2021 [11]	N/A	N/A	N/A	Very Good	N/A	Very Good	N/A	N/A
E Juniper 1993 [196]	N/A	N/A	N/A	Adequate	Adequate	Very Good	N/A	Very Good
E Sheppard, 2019 [30]	N/A	N/A	N/A	N/A	N/A	Very Good	N/A	N/A
L Gee, 2020 [77]	Doubtful	Very Good	N/A	Very Good	Inadequate	Very Good	Very Good	Very Good
C Fisher,2019 [89]	N/A	Very Good	N/A	Doubtful	N/A	Very Good	Very Good	Very Good
HF Alexandre, 2021 [51]	N/A	N/A	N/A	Very Good	Very Good	Very Good	N/A	N/A
JJ Swigris, 2010 [215]	N/A	N/A	N/A	N/A	N/A	Very Good	N/A	N/A
WF Aguiar,2021 [81]	N/A	Very Good	Doubtful	Very Good	Very Good	Very Good	Very Good	Very Good
E Bulcun et al.2015 [73]	N/A	N/A	Doubtful	Very Good	N/A	Very Good	N/A	N/A

MA Oliveira et al., 2005 [65]	N/A	N/A	N/A	Very Good	N/A	Very Good	N/A	Very Good
CO De Camargo et al., 2020 [124]	N/A	Very Good	N/A	Very Good	N/A	Very Good	Very Good	N/A
E Cenedese et al., 2006 [177]	N/A	N/A	N/A	Inadequate	N/A	Very Good	N/A	Very Good
C Wilson et al., 1997 [127]	N/A	Very Good	N/A	Doubtful	N/A	Very Good	N/A	N/A
C Kronberger et al., 2023 [16]	N/A	N/A	N/A	N/A	N/A	Very Good	N/A	N/A
E Calik-Kutukcu et al., 2022 [181]	N/A	N/A	N/A	Very Good	N/A	Very good	N/A	N/A
GP Reinaldo et al., 2022 [50]	N/A	N/A	N/A	Very Good	N/A	Very good	N/A	N/A

Abbreviations: ref – reference; N/A - Not applicable.

Table S6. Methodological quality of randomized clinical trials included assessed by Pedro.

Author and year	Ran	C-A	Bas.	BS	BT	BA	Follow-up	ITA	BGC	Point	Total score	Class
MAM Zadeh et al., 2013 [123]	1	1	1	0	0	0	0	1	1	1	6	Good
Y Liu et al., 2011 [205]	1	1	1	0	0	0	0	1	1	1	7	Good
LJ Okamoto et al., 1996 [72]	1	1	1	1	1	1	1	1	1	0	8	Good
CM Oliveira et al., 2016 [54]	1	1	1	0	0	0	0	1	1	1	6	Good
HJ Pai et al., 2015 [57]	1	1	0	1	1	1	0	1	1	1	8	Good
AM Smith et al., 2012 [59]	1	0	1	0	0	0	0	1	1	1	4	Fair
J Ware et al., 1998 [69]	1	0	1	1	1	0	0	1	1	1	7	Good
B Kligler et al., 2011 [61]	1	1	1	0	0	0	0	1	1	1	6	Good
N Newhouse et al., 2016 [55]	1	1	1	0	0	1	1	1	1	1	8	Good
MDM Martínez-García et al., 2006 [126]	1	0	1	1	1	0	0	1	1	1	7	Good
LM Dowman et al., 2017 [135]	1	1	1	0	0	1	1	1	1	1	8	Good
RM du Bois et al., 2011 [94]	1	0	1	0	0	0	0	1	0	1	4	Fair
B Vainshelboim et al., 2014 [34]	1	1	1	0	0	0	0	1	1	1	6	Good
B Vainshelboim et al., 2015 [33]	1	1	1	0	0	0	1	1	1	1	7	Good
WP Drake et al., 2013 [138]	1	0	1	0	0	0	0	1	1	1	5	Fair

MN Karadallı et al., 2016 [137]	1	1	1	0	0	0	0	1	1	1	6	Good
I Naz et al., 2018 [88]	1	1	1	0	0	0	0	1	1	1	6	Good
R Benza et al., 2011 [170]	1	1	1	1	1	1	0	1	1	1	9	Excellent
N Galiè et al., 2008 [114]	1	1	1	1	1	0	0	1	1	1	8	Good
N Galiè et al., 2005 [161]	1	1	1	1	1	0	0	1	1	1	8	Good
N Galiè et al., 2005 [160]	1	1	1	1	1	0	1	1	1	1	9	Excellent
N Galiè et al., 2009 [159]	1	0	1	1	1	0	0	1	1	1	7	Good
C Gp et al., 2009 [112]	1	0	1	1	1	0	0	1	1	1	7	Good
L González-Saiz et al., 2017 [28]	1	1	1	1	0	1	0	1	1	1	8	Good
VV McLaughlin et al., 2006 [176]	1	1	1	1	1	0	0	1	1	1	8	Good
D Mereles et al., 2006 [116]	1	1	1	0	0	1	0	1	1	1	7	Good
RJ Oudiz et al., 2012 [157]	1	0	1	1	0	1	0	1	0	0	5	Fair
LJ Rubin et al., 2015 [151]	1	1	1	1	1	0	0	1	1	1	8	Good
Y Zhuang et al., 2014 [154]	1	0	1	1	1	0	0	1	1	1	7	Good
DB Badesch et al., 2000 [179]	1	1	1	0	0	0	0	1	1	1	6	Good
SM Lage et al., 2011 [183]	1	1	1	0	0	1	1	0	1	1	7	Good
KB Evaristo et al., 2020 [184]	1	0	1	0	0	1	1	0	1	1	6	Good
S Majd et al., 2020 [185]	1	0	1	0	0		1	0	1	1	5	Fair

G Georga et al., 2019 [211]	1	1	1	0	0	0	0	1	1	1	6	Good
N Duruturk et al., 2018 [122]	1	1	1	0	0	0	0	0	1	1	5	Fair
PD Freitas et al., 2017 [186]	1	0	1	0	0	1	0	0	1	1	5	Fair
J Ma et al., 2017 [212]	1	1	1	0	0	1	1	1	1	1	8	Good
A Refaat et al., 2015 [187]	1	0	1	0	0	0	1	0	1	1	5	Fair
TZ Rondinel et al., 2015 [189]	1	1	1	0	0	0	0	0	1	1	5	Fair
S Turner et al., 2011 [60]	1	1	1	0	0	1	1	1	1	1	8	Good
R Vempati et al., 2009 [190]	1	0	1	0	0	0	0	0	1	1	4	Fair
W Busse et al., 2009 [193]	1	0	1	1	1	0	0	0	1	1	6	Good
G Riccioni et al., 2002 [192]	1	0	1	0	0	0	0	0	1	1	4	Fair
A José et al., 2021 [210]	1	1	1	0	0	0	1	1	1	1	7	Good
KA Lavery et al., 2011 [125]	1	1	1	0	0	1	1	1	1	1	8	Good
KB Knudsen et al., 2017 [197]	1	0	1	0	0	0	1	0	1	1	5	Fair
CA Sandsund et al., 2011 [203]	1	1	1	0	0	1	0	1	1	1	7	Good
AC Young et al., 2007 [204]	1	0	1	1	0	0	0	0	1	1	5	Fair
AR Koczulla et al., 2020 [23]	1	1	1	0	0	1	0	0	1	1	6	Good
K Janssen et al., 2020 [132]	1	0	1	0	0	0	1	1	1	1	6	Good
R Vis et al., 2020 [84]	1	1	1	1	1	1	1	1	1	1	10	Excellent

AC Theodore et al., 2012 [96]	1	0	1	0	0	1	1	1	1	1	7	Good
FX McCormack et al., 2011 [214]	1	1	1	1	0	1	1	1	1	1	9	Excellent
BO Kahraman et al., 2020 [35]	1	1	1	0	0	1	0	0	1	1	6	Good
GK Aslan et al., 2020 [213]	1	1	1	0	0	1	0	0	1	1	6	Good
H Karapolat et al., 2019 [104]	1	0	1	0	0	1	0	0	1	1	5	Fair
Babu et al., 2019 [105]	1	1	1	0	0	0	0	1	1	1	6	Good
I Laoutaris et al., 2015 [108]	1	0	1	0	0	1	0	0	1	1	5	Fair
AE Frost et al., 2015 [152]	1	0	1	0	0	0	1	0	1	1	5	Fair
J Pepke-Zaba et al., 2009 [113]	1	0	1	1	0	1	0	0	1	0	5	Fair
J Shen et al., 2005 [178]	1	0	1	0	0	1	0	0	1	0	4	Fair
DJ Webb et al., 2015 [153]	1	0	1	1	0	1	1	0	1	1	7	Good
Ghofrani et al., 2013 [156]	1	1	1	1	0	1	0	1	1	1	8	Good
T Van der Molen et al., 1998 [194]	1	0	1	1	0	1	1	1	1	1	8	Good
H Essam et al., 2022 [129]	1	0	1	0	0	0	1	0	1	1	5	Fair
O Atalay et al., 2022 [21]	1	0	1	0	0	1	1	0	1	1	6	Good
S Zaki et al., 2023 [128]	1	0	1	0	0	1	1	1	1	1	7	Good
AW Creamer et al., 2019 [133]	1	0	1	0	0	0	0	0	1	0	7	Good

PEDro scores of 0-3 are considered 'poor', 4-5 'fair', 6-8 'good', and 9-10 'excellent'. **Abbreviations:** Ran – randomization; C-A – Concealed allocation; Bas – Baseline group comparation; BS – Blind subjects; BA – Blind assessors; BT – Blind therapists; ITA – Intention-to-treat analysis; BGC – Between group comparisons; Point – Point estimates and variability.; Class – Classification.

Table S7. Methodological quality of included studies assessed by Downs and Black checklist.

Author and year	Score total/28	Classification
JJ Swigris et al., 2012 [97]	14	Poor
M Majewski et al., 2015 [29]	14	Poor
H Tomioka et al., 2016 [90]	14	Poor
A Meyer et al., 2015 [56]	19	Fair
T Radtke et al., 2016 [4]	11	Poor
CM Nolan et al., 2018 [42]	15	Fair
S Dalichau et al., 2010 [91]	15	Fair
S Dalichau et al., 2020 [83]	19	Fair
EH Alhamad et al., 2015 [93]	17	Fair
JJ Swigriss et al., 2011 [98]	15	Fair
E Ozpelit et al., 2015 [167]	14	Poor
SA Segrera et al., 2017 [147]	14	Poor
R Souza et al., 2007 [115]	12	Poor
R Souza et al., 2005 [118]	13	Poor
N Tanabe et al., 2017 [148]	15	Fair
L Tokgozoglu et al., 2009 [171]	11	Poor
U Ochmann et al., 2012 [58]	15	Fair
CA Dyer et al., 1999 [68]	16	Fair
FF Canuto et al., 2012 [2]	12	Poor
M Matheson et al., 2012 [66]	11	Poor
K Stavem et al., 2000 [67]	13	Poor
S Pakhale et al., 2015 [188]	20	Good
P Mendes et al., 2013 [207]	12	Poor
P Blanc et al., 1997 [70]	16	Fair
JS Choi et al., 2005 [191]	17	Fair
V Siroux et al., 2008 [62]	15	Fair
Y Tohda et al., 2006 [63]	16	Fair
JM Guilemany et al., 2006 [76]	15	Fair
PS Jacques et al., 2012 [74]	11	Poor
Z McKeough et al., 2020 [22]	12	Poor
DK McClish et al., 2005 [64]	12	Poor
M Gruet et al., 2016 [5]	12	Poor
MDM Martinez-Garcia et al., 2020 [38]	12	Poor
KCA Aguiar et al., 2017 [198]	12	Poor
VJ Ribeiro Moço et al., 2015 [199]	11	Poor
JT Penafortes et al., 2014 [200]	11	Poor
EJ Dill et al., 2013 [201]	14	Poor
L Kelemen et al., 2012 [202]	11	Poor
P Mendes et al., 2015 [26]	12	Poor
NF Braz et al., 2016 [136]	11	Poor
Z Sözener et al., 2010 [139]	12	Poor
S Ohno et al., 2005 [101]	11	Poor

K Pilzak et al., 2018 [87]	10	Poor
SA Guler et al., 2019 [41]	12	Poor
J Briand et al., 2018 [14]	12	Poor
F Lumetti et al., 2015 [95]	12	Poor
JÁ Chang et al., 1999 [102]	11	Poor
C Ryerson et al., 2014 [44]	21	Good
CM Nolan et al., 2018 [43]	18	Fair
K Ebihara et al., 2021 [131]	15	Fair
DK Ozen et al., 2020 [162]	13	Poor
E Yalnız et al., 2019 [86]	12	Poor
PV Santana et al., 2019 [134]	13	Poor
V Krishnan et al., 2008 [99]	13	Poor
R Zhao et al., 2020 [82]	13	Poor
M Kukkonen et al., 2016 [106]	12	Poor
M Aldemir et al., 2018 [144]	12	Poor
LA Matura et al., 2012 [110]	11	Poor
LA Matura et al., 2014 [109]	11	Poor
SK Saha et al., 2016 [149]	12	Poor
R Souza et al., 2007 [175]	11	Poor
J White et al., 2006 [117]	12	Poor
N Zafrir et al., 2007 [174]	13	Poor
L Nakazato et al., 2021 [18]	11	Poor
A Arvanitaki et al., 2020 [142]	10	Poor
K Karauzum et al., 2018 [143]	12	Poor
R Naal et al., 2018 [166]	15	Fair
R Condliffe et al., 2009 [158]	13	Poor
C Mihai et al., 2017 [145]	14	Poor
M Waligora et al., 2017 [146]	14	Poor
SA Mouratoglou et al., 2014 [155]	13	Poor
N Malik et al., 2012 [169]	12	Poor
M Yigla et al., 1997 [180]	13	Poor
OA Minai et al., 2007 [172]	12	Poor
L Godinas et al., 2016 [150]	13	Poor
M Feltrim et al., 2008 [121]	10	Poor
D Langer et al., 2012 [120]	13	Poor
JN Justice et al., 2019 [24]	18	Fair
N Chueamuangphan et al., 2014 [168]	17	Fair
EMC Chau et al., 2007 [173]	17	Fair
C Zimmermann et al., 2007 [100]	13	Poor
B Wallaert et al., 2020 [13]	13	Poor
R Hena et al., 2018 [49]	13	Poor
V Rusanov et a., 2008 [53]	12	Poor
K Bunclark et al., 2021 [164]	21	Good
AL Lee et al., 2009 [75]	13	Poor
B Vainshelboin et al., 2019 [32]	18	Fair
E Juniper et al., 1994 [195]	13	Poor

A Miozzo et al., 2023 [119]	17	Fair
CM Nolan et al., 2023 [39]	21	Good
C Paixão et al., 2023 [7]	17	Fair
M Alotaibi et al., 2023 [140]	17	Fair
C Keen et al., 2023 [15]	17	Fair
M Hanada et al., 2022 [46]	13	Poor
K Oishi et al., 2022 [8]	14	Poor
K Fujita et al., 2022 [130]	15	Fair
PF Tremblay Labrecque et al., 2022 [9]	14	Poor
N Vardar-Yagli et al., 2022 [48]	13	Poor
I Ozsoy et al., 2022 [37]	14	Poor
M Okamura et al., 2022 [27]	15	Fair
M Pereira et al., 2022 [17]	12	Poor
JF Masa et al., 2022 [103]	15	Fair
L Matura et al., 2022 [141]	18	Fair
A Lee et al., 2022 [209]	14	Poor
A Freeman et al., 2021 [182]	18	Fair
S Unlu et al., 2022 [163]	17	Fair
K Oishi et al., 2023 [6]	12	Poor
T Koudstaal et al., 2019 [208]	19	Fair
F Aboelmagd et al., 2022 [79]	20	Good
SY Chikina et al., 2022 [31]	16	Fair
HA Babar et al., 2022 [45]	4	Poor
M Sikora et al., 2023 [78]	16	Fair
A Yilmaz et al., 2021 [20]	15	Fair
E Zampogna et al., 2021 [19]	18	Fair
F Machado et al., 2021 [80]	17	Fair
E Grunig et al., 2012 [111]	16	Fair
AA Perez et al., 2020 [47]	20	Good
EV Karelkina et al., 2020[165]	19	Fair

Downs & Black punctuation was categorize by quality in excellent (26–28), good (20–25), fair (15–19) and poor (⩽14).

Table S8. Functional performance protocols as per reported in the included studies.

Measure	Outcome	Better result of FP	Equipment	Description
1min-STS	Number of repetitions.	Higher number of repetitions	Standard chair (i.e. with no armrest, 40-48cm height, ideally with 90º hip and knee flexion) against a wall.	Patients seated with crossed arms is instructed to get up from the chair and sit down many times as possible in one-minute.
30sec-STS				Patients seated with crossed arms is instructed to get up from the chair and sit down many times as possible in 30 seconds.
3min-STS				Patients seated with crossed arms is instructed to get up from the chair and sit down many times as possible in 3 minutes.
5rep-STS	Time (seconds)	Shorter time spent		Patients seated with crossed arms is instructed to get up from the chair and sit down five times as quickly as possible and the duration taken to complete the 5 repetitions is timed.
4MGS	Velocity (m/s) of time (seconds).	Higher velocity or shorter time spent	Corridor (i.e. 4m or 8m)	The patient is instructed to walk, and the time spent to walk the four-metre is timed. The test can be performed in usual pace or as fast as possible.
SPPB	Score	Higher score	Chair and 4m corridor	Three aspects are evaluated and summarized in the total score: balance, gait-speed and strength. To assess balance, the patient is instructed to stay at least 10s in three positions (romberg, semi-tandem and tandem). Gait-speed is assessed by the time to walk four-metre and strength is assessed by the time to sit to stand five times as fast as possible.
TUG	Time (seconds)	Shorter time spent	Chair and 3m corridor	The patient is instructed to rise from a chair, walks 3 meters, turns, walks back, and sits down again. The protocol can be performed, in usual pace and as fast as possible without run.

8-FUGT	Time (seconds)	Shorter time spent	Chair and 2.40m corridor	The patient got up from the chair, walked around a cone that was placed 8 feet (2.40 m) from the chair, and returned to a seated position on the chair as fast as possible.
Glittre ADL	Time (minutes)	Shorter time reflects better functional performance	Three objects of 1 kg and backpack with weight (2.5Kg for women and 5Kg for men)	Consists of a 10-meter circuit in which the individual starts from a sitting position, walks, goes up and down two interposed steps and walks again until reaching a shelf, individually adjusted according to the height of the shoulder and waist. The patients were instructed to complete five laps in the shortest time, using a backpack.
CS-PFP	Score	Higher score	Furniture developed to simulate ADL (e.g. Laundry, washer, sandbag...)	The CS-PFP test is a series of 10 tasks covering everyday life activities required to maintain independence. Subjects are asked to complete the tasks at maximal effort.
15-stepsC	Time (seconds)	Shorter time spent	Step (25 cm length; 50 cm width; 20 cm)	Patients were asked to climb up and down the step 15 times as fast as they could, without any fixed pacing.
PPT	Score	Higher score	7lb book; lab coat; penny; 4 flights of stairs and a chair.	PPT assesses multiple domains of individuals' functional capacity using tasks that simulate ADLs, and has two versions: 7- and 9-items.

Abbreviations: FP – Functional performance; 1min-STS – 1minute sit-to-stand; 4MGS – four-metre gait speed; 30sec-STS – 30 seconds sit-to-stand; 5rep-STS – 5 repetitions sit-to-stand; SPPB – short physical performance battery; TUG – timed-up-and-go; 8-FUGT – 8-foot-up-and-go; Glittre ADL – Glittre Activities of daily living; CS-PFP – continuous scale physical function performance test; 3min-STS – 3-minute sit-to-stand; 15-stepsC – 15 steps climbing; PPT – Physical performance test.

Table S9. Metric properties and associations with negative outcomes of performance-based tests for only IPF patients.

Performance-based tests	Validity	Reliability	Interpretability	Associations with events related to the curse of CRD or prognosis
1min-STS	FVC%pred (r=0.48; p<0.05); TCLO%pred (r=0.47; p<0.001)[11] *(Very Good)*	Test-retest [ICC:0.92 (95% CI: 0.86-0.95)][11] *(Very Good)*		
3min-STS	FVC%pred (r=0.43; p<0.05); TCLO%pred (r=0.55; p<0.001)[11] *(Very Good)*	Test-retest [ICC:0.96 (95% CI: 0.92-0.98)][11] *(Very Good)*		
4MGS	6MWD (r=0.76; p>0.01); KBILD total (r=0.44; p<0.001); MRC (r=-0.56; p>0.05); GAP index (r=-0.41; p=0.002)[42] *(Very Good)*	Intra-rater (ICC=0.98; 95% CI: 0.97-0.99) and Inter-rater (0ICC=0.99; 95% CI: 0.99-0.99)[42] *(Doubtful to Very Good)*		4MGS<0.08m/s is independent predictor of hospitalisation [HR: 2.63 (1.0-6.8); p=0.04] and all-cause mortality [HR: 2.76 (1.1-6.5); p=0.02)[43] *(Fair)* Decline in 4MGS≥0.07m/s is associated with death in 6 months (Kaplan-Meier curves comparing decline≥0.07m/s versus decline≤0.07 m/s; p=0.007)[39] *(Good)*
8-FUGT				Performance in 8-FUGT>6.9s is associated with hospitalisation [HR: 14.1 (3.5-56); p<0.001] and mortality [HR: 55.4 (5-592); p=0.001][32] *(Fair)*
Glittre ADL	6MWD (r=-0.90; p<0.001)[50] *(Very Good)*	Test-retest (ICC=0.96; 95% CI: 0.74-0.99)[50] *(Very Good)*		

CS-PFP	SGRQ – activities (r=-0.80; p=0.0002); 6MWD (r=0.66; p=0.008); SF-36 – PFd (r=0.64; p=0.007); D_{LCO} %predicted (r=0.67; p=0.006); FVC %predicted (r=0.63; p=0.009)[52] *(Very Good)*	Intra-rater (ICC:0.83)[52] *(Adequate)*[A]		

Interpretability refers to minimal clinical important difference (MCID), minimal detectable change was not considered; Risk of bias for studies investigating validity, reliability and interpretability was classified by COSMIN checklist *(Inadequate, Doubtful, Adequate, Very Good)*; Studies investigating association with negative outcomes was assessed and classified by Downs & Black checklist and PEDro *(Excellent, Good, Fair and Poor)*, according to study design. Multivariable linear regression models were fitted to examine the independent association by the coefficient of determination (r^2). Of note, instruments included in the systematic review not described in this table do not have psychometric properties or association with mortality, hospitalization reported. **Abbreviations:** MCID – Minimal clinical important difference; 1min-STS – 1minute sit-to-stand; 4MGS – four-metre gait speed; 5rep-STS – 5 repetitions sit-to-stand; 8-FUGT – 8-foot-up-and-go; Glittre ADL – Glittre Activities of daily living; CS-PFP – continuous scale physical function performance test; 3min-STS – 3-minute sit-to-stand; PFd – Physical function domains; 6MWD – distance in six-minute walk test; QS – Quadriceps strength; HGS – Handgrip strength; D_{LCO} – Diffusion capacity of carbon monoxide; T_{LCO} – transfer factor for carbon monoxide; mMRC – Modified medical respiratory council dyspnoea score; GAP index – Gender, age and lung physiology index; ICC – Intraclass correlation coefficient; FVC – Forced vital capacity; CI – confidence interval; rep – repetitions. [A] 95% CI was not reported in the study included.

Table S10. Psychometric properties and associations with negative outcomes of patient-reported tools for only IPF patients.

Performance-based tests	Validity	Reliability	Interpretability	Associations with events related to the curse of CRD or prognosis
SF-36 (PFd or PCS)	BDI (r=0.25; p<0.05); 6MWD (r=0.44; p<0.0001); mMRC (r=-0.48; p<0.0001); D_{LCO} %predicted (r=0.36; p<0.001); FVC %predicted (r=0.35; p<0.05); NYHA (r=-0.33; p<0.0001) [85] *(Very Good)*	No difference in test-retest (p>0.05) [85] *(Doubtful)*	MCID of 4 points in PCS [85] *(Very Good)*	
SGRQ-I (activities)	MRC (r=0.71; p<0.0001); SF36 – PFd (r=-0.71; p<0.05); SF36 – PCS (r=-0.32; p>0.05) [81] *(Very Good)*	Intra-rater [ICC:0.93 (95% CI: 0.85-0.97)] and Inter-rater [ICC: 0.88 (95% CI: 0.77-0.94)] [81] *(Very Good)*		

Interpretability refers to minimal clinical important difference (MCID), minimal detectable change was not considered; Risk of bias for studies investigating validity, reliability and interpretability was classified by COSMIN checklist *(Inadequate, Doubtful, Adequate, Very Good)*; Studies investigating association with negative outcomes was assessed and classified by Downs & Black checklist and PEDro *(Excellent, Good, Fair and Poor)*, according to study design. Multivariable linear regression models were fitted to examine the independent association by the coefficient of determination (r^2). Of note, instruments included in the systematic review not described in this table do not have psychometric properties or association with mortality, hospitalization reported. **Abbreviations:** MCID – Minimal clinical important difference; SF-36 – Medical Outcomes Study 36-item Short Form of Health Survey; SGRQ – Saint George's Respiratory Questionnaire; PFd – Physical function domains; PCS – Physical component subscale; D_{LCO} – Diffusion capacity of carbon monoxide; MRC – Modified medical respiratory council dyspnoea score; 6MWD – distance in six-minute walk test; BDI – Body dyspnea index; NYHA – New York health assessment; FVC – Forced vital capacity; ICC – Intraclass correlation coefficient; CI – confidence interval.

Figure S1. Number of studies per chronic respiratory disease.

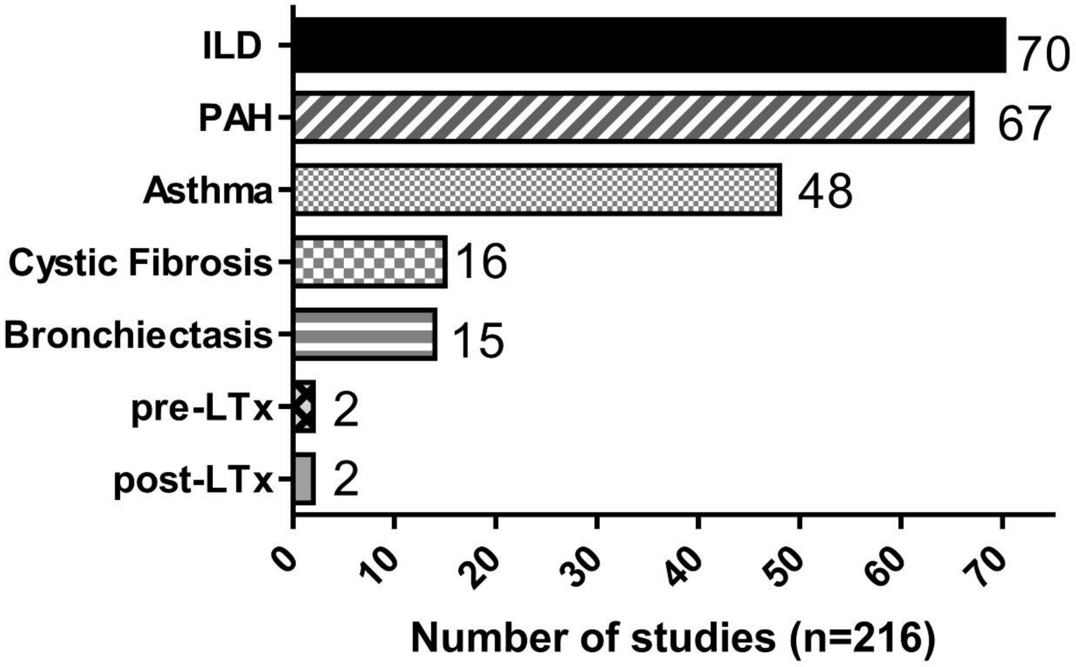

Abbreviations: PAH – Pulmonary Arterial Hypertension; ILD – Interstitial Lung Disease; pre-LTx – patients with chronic respiratory disease waiting to lung transplantation; post-LTx – patients with chronic respiratory disease who underwent to lung transplantation.

Information regarding patient-reported and performance-based instruments in patients pre and post lung transplantation (LTx).

This review included only two studies with patients waiting for (pre-LTx) [119,121] and two studies with patients who underwent to lung transplantation (post-LTx) [47,120], as studies investigating FP instruments with results considering mixed chronic respiratory diseases were excluded. This review included few studies pre-LTx and post-LTx (i.e. 2 studies for each), therefore it was unable to support the use of FP instruments for patients for these subgroups of disease. The SPPB, the PFd of SF-36 and activities domain of SGRQ were the only instruments used in the included studies with pre-LTx and post-LTx patients, but no metric or psychometric properties were investigated. Minimal clinical important difference was reported for the SPPB (i.e. one point in the total score) [47] in patients with CF post-LTx. Additionally, one study [217] investigated the impact of changes in the PCS of SF-36 on mortality, an increase of at least 50% in PCS over 6 months was associated with higher survival rates. Of note, this result belongs to a study excluded from the present review as it was not possible to guarantee the absence of COPD patients in the cohort.

Description of the performance-based and patient-report tools, as well as their properties and clinical implications

Performance-based tests

Sit-to-stand Test: Four different protocols of the sit-to-stand test in non-COPD CRD patients were reported: one-minute sit-to-stand (1min-STS), 5 repetitions sit-to-stand (5rep-STS), 30 seconds sit-to-stand (30sec-STS), and 3 minutes sit-to-stand (3min-STS). The most used protocol of the sit-to-stand in non-COPD CRD patients was the 1min-STS. Eighteen studies used this protocol: two in asthma [1, 2], three in CF [3-5], nine in ILD [6-14] and four in PAH [15-18]. The correlation between the performance on the 1min-STS and exercise capacity [i.e. peak work rate (Wpeak) and peak oxygen uptake, (VO2 peak) from the CPET, and 6MWD] and peripheral muscle strength (i.e. quadriceps strength and handgrip force) was investigated in asthma, CF, ILD and PAH. However, validation was reported only in studies including people with CF, ILD and PAH [4, 10-13, 16].

Construct validity was demonstrated with exercise capacity [i.e. peak work rate (W_{peak}) and peak oxygen uptake, (VO_2 peak) in people with CF [3, 4] and with distance in 6MWT (6MWD)] in people with ILD [12, 14] and PAH [16]. Good to excellent reliability in intra-rater analysis and good to excellent reliability in inter-rater analysis was observed in asthma, CF and ILD [1, 4, 10, 13]. The minimal clinical important difference (MCID) of the test was only described in people with CF, and the MCID reported was 5 repetitions [4].

The 5rep-STS were used in thirteen studies: three in asthma [1, 19, 20], two in bronchiectasis [21, 22], six in ILD [9, 10, 23-26] and two in PAH [27, 28]. Validity was demonstrated when the 5rep-STS was correlated with the 6MWD in people with asthma [1] and with quadriceps muscle strength in people with ILD [10]. Good intra-rater and good to excellent in inter-rater reliability were reported in studies of people with asthma and ILD [1, 10, 25]. The 30sec-STS was used in nine studies: two in asthma [1, 29], one in CF [30], four in ILD [10, 31, 33, 34] and two in PAH [35, 36]. Although metric properties were investigated in asthma, CF, ILD and PAH, validity was demonstrated only in CF [30] and PAH patients [36] (i.e. correlations with quadriceps strength). Good to excellent intra-rater and inter-rater reliability were achieved in asthma [1], ILD [10] and PAH patients [36].

The 3min-STS was assessed in only one study in people with ILD [11]. Validity was demonstrated when the 3min-STS was correlated with pulmonary function (i.e. Transfer factor of the lung carbon monoxide – T_LCO) and excellent test-retest reliability was found [11]. There were no studies reporting

associations with hospitalisation, mortality, or other negative clinical outcomes in any protocol sit-to-stand test. Further details of protocols of sit-to-stand are described in **Table 1** and in **e-Table 7**.

Four-metre gait speed (4MGS): Fourteen studies used 4MGS, two in asthma [1, 37], one in bronchiectasis [22] and CF [38], nine in ILD [10, 24, 25, 39-44] and one in PAH [27]. The usual gait speed protocol was commonly utilized, and additional information regarding the protocols used for the 4MGS in the included studies can be found in **e-Table 8**. The 4MGS was the FP test with the largest number of studies investigating its metric properties, mainly in ILD [10, 25, 40, 42]. The 4MGS demonstrates validity as evidenced by its correlations with handgrip strength and 6MWD in asthma [1] and ILD [25, 42]. Furthermore, it has shown associations with dyspnea and disease severity in ILD [40, 42]. It also presented good to excellent intra-rater and moderate to excellent inter-rater reliability in asthma [1] and ILD [10, 25, 42]. Performance in the 4MGS associates with disease severity (i.e. Forced vital capacity - FVC% and Diffusion capacity of the lung for carbon monoxide - D_{LCO}%) [41] and prognosis in ILD [42]. Slow gait speed (4MGS<0.8 m/s) is an independent predictor of all-cause mortality and non-elective hospitalisation and appears to be responsive to pulmonary rehabilitation in idiopathic pulmonary fibrosis [43]. Finally, a decline of 0.07m/s was associated with death in six months [39].

Short physical performance battery (SPPB): Ten studies applied SPPB to assess FP: two in asthma [1, 45], six in patients with ILD [9, 10, 24, 26, 32, 46], one in PAH patients [27] and one in patients with CF post-LTx [47]. Metric properties were investigated in asthma and ILD [1, 10], weak to moderate correlations with exercise capacity and handgrip force in asthma and ILD, although, validity was reached only in asthma patients [1, 10]. Moderate to good reliability in the intra-rater and inter-rater analysis was observed in asthma and ILD [1, 10]. There were no studies reporting associations with hospitalisation, mortality or other negative clinical outcomes.

Timed up and go (TUG): The TUG was mentioned in seven studies, including two studies in asthma [1, 20], one in bronchiectasis [48], two in ILD [10, 26], and two in PAH [35, 36]. Among these studies, two different protocols for the TUG were reported: one with a usual pace and another with a fast pace. However, the protocol most reported was the TUG performed at a usual pace. Metric properties were investigated in asthma, ILD and PAH. Validity was achieved in asthma with 6MWD [1] and in ILD with 6MWD and handgrip force [10]. Good to excellent intra-rater and inter-rater reliability was observed in asthma and ILD patients [1, 10], and also excellent intra-rater reliability was observed in PAH [36]. There were no studies reporting associations with hospitalisation, mortality, or other negative clinical outcomes.

8 foot up and Go (8-FUGT): The 8-FUGT was reported in three studies: one study in asthma [29] and two studies in ILD [26, 32]. Although, there were no studies reporting metric properties, performance above 6.9 seconds is associated with hospitalisation and mortality in ILD patients [32].

Glittre Activities of Daily Living test (Glittre ADL): Three studies used Glittre ADL test to assess FP in bronchiectasis [49] and ILD [50, 51]. Metric properties were assessed, validity was reached via correlations with 6MWD and total energy expenditure in daily physical activities in ILD patients [50, 51]. Also, excellent reliability was observed in test-retest analysis for ILD patients [50, 51]. There were no studies reporting metric properties in other CRDs and associations with hospitalisation, mortality or other negative clinical outcomes.

Continuous scale physical function performance (CS-PFP): One study using CS-PFP was found in ILD [52]. CS-PFP showed validity with the domain activities of SGRQ, and with the 6MWD, pulmonary function (FVC% and D_{LCO}%) and with PFd of SF-36. Additionally, it has excellent intra-rater agreement [52]. There were no studies reporting associations with hospitalisation, mortality or other negative clinical outcomes.

15 steps climbing (15-stepsC): Only one study in ILD used the 15 steps climbing test [53]. There were no studies reporting metric properties or associations with hospitalisation, mortality or other negative clinical outcomes.

Physical Performance Test (PPT): Only one study in ILD used the PPT [8]. There were no studies reporting metric properties or associations with hospitalisation, mortality or other negative clinical outcomes.

Patient-reported tools

Medical Outcomes Study 36-item Short-Form Health Survey (SF-36): The Physical Function domain (PFd) of the SF-36 questionnaire evaluates the effect of FP on daily activities, while the Physical Composite Score (PCS) takes into account other factors that affect overall physical well-being. Both outcomes were deemed measures of FP in this review. The SF-36 was the most frequently used questionnaire with 72 studies using PFd or PCS in all subgroups of diseases. Three studies [71, 85, 112] reported on the psychometric properties of SF-36, including PFd or PCS domains. The validity of PFd was investigated in asthma by correlating PFd with symptom scores and in ILD by correlating PFd with 6MWT, mMRC, pulmonary function (i.e. FVC% and D_{LCO}%), and baseline dyspnoea index (BDI).

Reliability was analysed only for ILD and the MCID of the tool was stablished for ILD (i.e. 4 points in PCS) [85] and PAH (i.e. 5 points in the PFd and 13 points for the PCS) [112]. No other measures of reliability were reported in any non-COPD CRDs. In one study, a significant association was found between the PCS and both depression and comorbidities in individuals with asthma [68].

Medical Outcomes Study 12-item Short Form Health Survey (SF-12): Three studies used SF-12: two in asthma [61, 205] and one in patients post-LTx [47]. There were no studies reporting metric properties or associations with hospitalisation, mortality or other negative clinical outcomes.

Saint George's Respiratory Questionnaire (SGRQ): The activities domain of the SGRQ can be used to describe FP. Thirty-nine studies used the activities domain of the SGRQ as FP measure in asthma [29, 68, 122, 123], bronchiectasis [21, 22, 75, 124-126], ILD [9, 23, 24, 33, 34, 40, 50-52, 81, 88, 89, 97, 100, 102, 128-139] and patients pre-LTx [121]. The psychometric properties of the SGRQ were established in patients with bronchiectasis and ILD [81, 127]. It is worth mentioning that one study, which was excluded from this review, demonstrated the validity and reliability of the SGRQ in asthma patients, including children [218].

In ILD, the activities domain of the SGRQ showed validity with PFd of SF-36 and with MRC [81], as well as with PCS and Shuttle distance in bronchiectasis [127]. Good to excellent reliability was achieved in intra-rater and inter-rater analysis in ILD patients [81]. Reliability was not investigated in other diseases and no associations with mortality, hospitalisation or other negative clinical outcomes were reported.

World Health Organization functional class (WHOfc): The WHOfc was reported only in PAH patients, in thirty-four studies [15, 16, 27, 105-110, 112, 114, 117, 140-161]. None of the included studies reported psychometric properties of the instrument. Although, WHOfc≥3 was associated with clinical failure in PAH [155] and PAH patients in classes III and IV appear to have higher risk of mortality than the other two classes [158].

New York Heart Association functional class (NYHA): NYHA was reported in twenty-four studies, four in ILD [53, 85, 139, 162] and twenty in PAH [17, 36, 163-180], but none reported psychometric properties. Mortality rates were more prevalent in PAH patients classified as NYHA III or IV [171].

Asthma Quality of Life Questionnaires (AQLQ) and Mini-AQLQ: The AQLQ was used in twenty-one studies (all in asthma) [56, 60, 61, 69, 71, 181-196]. A shorter version of AQLQ (the Mini-AQLQ, M-AQLQ) was reported in two studies [211, 212]. The AQLQ was validated using the

questionnaire of asthma control questionnaire, and questionnaires of general health or quality of life [196]. The AQLQ has excellent intra-rater reliability [196] and the MCID of the tool has been described as 0.51 points in activities domain [195]. There were no studies reporting association between the AQLQ and mortality or hospitalisation. No psychometric properties or association with mortality of hospitalisation were observed in Mini-AQLQ.

Cystic Fibrosis Quality of Life (CFQoL): The PFd of the CFQoL was utilized to assess FP in ten studies [4, 77, 197-204], all conducted in cystic fibrosis (CF) patients. The validity of the CFQoL was established by demonstrating a correlation with the PFd of the SF-36. Additionally, strong intra-rater reliability was demonstrated [77]. There were no studies reporting associations with hospitalisation, mortality or other negative clinical outcomes.

Patient-Reported Outcomes Measurement Information System (PROMIS-29): The PFd of the PROMIS-29 was considered as FP domain. Three studies used PROMIS-29 [89, 92, 206], all investigated psychometric properties in ILD, two studies in connective tissue disease associated with pulmonary fibrosis patients [89, 92] and one with IPF patients [206]. The PFd from the Promis-29 correlated with the PFd from the SF-36 and the activity domain from the SGRQ. Additionally, it correlated with the PCS domain from the SF-36 and the Health Assessment Questionnaire-Disability Index (HAQ-DI) [89, 92]. Moderate reliability in intra-rater analysis was found [89, 206] and internal consistency of the PFd of the PROMIS-29 was 41% [89]. Also, no domain of PROMIS-29 appears to be responsive to changes in lung function [89]. There were no studies reporting associations with hospitalisation, mortality or other negative clinical outcomes.

Living with Asthma Questionnaire (LWAQ): The physical health construct of the LWAQ was used to assess FP. LWAQ was used in three studies in patients with asthma [63, 71, 194]. Moderate correlations were observed through physical health construct with PCS of SF-36 and validity was reached with total symptoms score [71]. Internal consistency was investigated only for total score of LWAQ. There were no studies reporting associations with hospitalisation, mortality or other negative clinical outcomes.

Asthma Quality of Life from the "Escola Paulista de Medicina" (QOL- EPM): The physical limitation domain of the QOL-EPM was used as FP measure in three studies in asthmatic patients [57, 65, 207]. Validity was not reached, weak to moderate correlations were reported with SF-36 domains [65] and good test-retest reliability was reported [65]. There were no studies reporting associations with hospitalisation, mortality or other negative clinical outcomes.

Cambridge Pulmonary Hypertension Outcome Review (CAMPHOR): The activity domain of the CAMPHOR was used to assess FP. Three studies used the activity domain of CAMPHOR in patients with PAH [110, 164, 208]. The MCID of the activity domain is 4 points [164]. There were no studies reporting validity, reliability or associations with hospitalisation, mortality or other negative clinical outcomes.

London Chest Activities Of Daily Living Scale (LCADL): Physical activity score was used as FP measure in three studies in patients with asthma and ILD [50, 122, 181]. There were no studies reporting psychometric properties or associations with hospitalisation, mortality or other negative clinical outcomes.

Quality of Life Bronchiectasis questionnaire (QoL-B): Physical functioning domain from the QoL-B was used to assess FP. Three studies used QoL-B in bronchiectasis [124, 209, 210]. The PFd of QoL-B demonstrated validity with activities domain of SGRQ. Also, the PFd of QoL-B demonstrated convergent validity with mMRC and the incremental shuttle walk test and discriminative validity with mMRC [124]. Excellent test-retest reliability was reported [124]. There were no studies reporting associations with hospitalisation, mortality, or other negative clinical outcomes.

Minessota Living with Heart Failure Questionnaire (MLHFQ): The physical subscore was used to assess FP in two studies with PAH patients [177, 213]. Moderate correlations were found between the MLHFQ and NYHA functional class, and between MLHFQ and 6MWD [177]. It was demonstrated that the physical subscore of the MLHFQ has good test-retest reliability and internal consistency [177]. Patients with MLHFQ score≥40 (overall outcome) had a significantly worse prognosis than those with a score<40 [177].

Functional performance inventory (FPI): Only one study investigated functional performance through FPI in ILD [214]. There were no studies reporting psychometric properties or associations with hospitalisation, mortality or other negative clinical outcomes.

Seattle Obstructive Lung Disease Questionnaire (SOLQ): The PFd from the questionnaire was used as FP measure. One study used the PFd from the SOLQ in patients with bronchiectasis [73]. The PFd of SOLQ was validated using the PCS domain of SF-36 [73]. The SOLQ demonstrated good reliability in intra-rater analysis and good internal consistency [73]. The PFd of SOLQ was associated with exacerbation frequency [73], but no associations with hospitalisation or mortality is reported.

Eastern Cooperative Oncology Performance Status (ECOPs): The ECOPs was used in one study including patients with ILD [8]. There were no studies reporting psychometric properties or associations with hospitalisation, mortality or other negative clinical outcomes.

PROMIS Physical Function Short Form 8a (PROMIS-PF): The physical function domains of PROMIS-PF was used in one study with PAH patients [141]. No psychometric properties or associations with hospitalisation, mortality or other negative clinical outcomes were reported in included study.

Multi-Dimensional Health Assessment Questionnaire (MDHAQ): The PFd was used as a measure of FP in one study in ILD associated with connective tissue disease [215]. Although this study investigated internal consistency and correlations, this analysis was not performed to the specific domain of FP. There were no studies reporting psychometric properties or associations with hospitalisation, mortality or other negative clinical outcomes.

Health Assessment Questionnaire – Disability Index (HAQ-DI): The total score of HAQ-DI was used as PFd in one study included with ILD patients associated with rheumatoid arthritis [216]. Although psychometric properties were described in patients with rheumatoid arthritis, in included study no psychometric properties or associations with hospitalisation, mortality or other negative clinical outcomes were reported in patients with ILD.

References

1. Oliveira, J.M., et al., *Functional tests for adults with asthma: validity, reliability, minimal detectable change, and feasibility.* J Asthma, 2020: p. 1-9.
2. Freitas Canuto, F., et al., *[Neurophysiological and functional assessment of patients with difficult-to-control asthma].* Rev Port Pneumol, 2012. **18**(4): p. 160-5.
3. Radtke, T., et al., *The 1-min sit-to-stand test in cystic fibrosis - Insights into cardiorespiratory responses.* J Cyst Fibros, 2017. **16**(6): p. 744-751.
4. Radtke, T., et al., *The 1-min sit-to-stand test--A simple functional capacity test in cystic fibrosis?* J Cyst Fibros, 2016. **15**(2): p. 223-6.
5. Gruet, M., et al., *The 1-Minute Sit-to-Stand Test in Adults With Cystic Fibrosis: Correlations With Cardiopulmonary Exercise Test, 6-Minute Walk Test, and Quadriceps Strength.* Respir Care, 2016. **61**(12): p. 1620-1628.
6. Oishi, K., et al., *Detection of impaired gas exchange using the 1-minute sit-to-stand test in patients with interstitial lung disease.* Respir Investig, 2023. **61**(2): p. 186-189.
7. Paixao, C., et al., *Lifestyle integrated functional exercise for people with interstitial lung disease (iLiFE): A mixed-methods feasibility study.* Heart Lung, 2023. **60**: p. 20-27.
8. Oishi, K., et al., *Author Correction: The 1-minute sit-to-stand test to detect desaturation during 6-minute walk test in interstitial lung disease.* NPJ Prim Care Respir Med, 2022. **32**(1): p. 9.
9. Tremblay Labrecque, P.F., G. Dion, and D. Saey, *Functional clinical impairments and frailty in interstitial lung disease patients.* ERJ Open Res, 2022. **8**(4).
10. Zamboti, C., et al., *Functional performance tests in interstitial lung disease: Impairment and measurement properties.* Respiratory Medicine, 2021. **184**: p. 106413.
11. Fedi, A., et al., *Concurrence of 1- and 3-Min Sit-to-Stand Tests with the 6-Min Walk Test in Idiopathic Pulmonary Fibrosis.* Respiration, 2021. **100**(7): p. 571-579.
12. Tremblay Labrecque, P.F., et al., *Validation and Cardiorespiratory Response of the 1-Min Sit-to-Stand Test in Interstitial Lung Disease.* Med Sci Sports Exerc, 2020. **52**(12): p. 2508-2514.
13. Wallaert, B., et al., *The 1-minute sit-to-stand test to evaluate quadriceps muscle strength in patients with interstitial lung disease.* Respir Med Res, 2020. **78**: p. 100773.

14. Briand, J., et al., *The 1-minute sit-to-stand test to detect exercise-induced oxygen desaturation in patients with interstitial lung disease.* Ther Adv Respir Dis, 2018. **12**: p. 1753466618793028.

15. Keen, C., et al., *Pulmonary Hypertension and Measurement of Exercise Capacity Remotely: Evaluation of the 1-min Sit-to-Stand Test (PERSPIRE) - a cohort study.* ERJ Open Res, 2023. **9**(1).

16. Kronberger, C., et al., *Functional capacity testing in patients with pulmonary hypertension (PH) using the one-minute sit-to-stand test (1-min STST).* PLoS One, 2023. **18**(3): p. e0282697.

17. Pereira, M.C., et al., *One minute sit-to-stand test as an alternative to measure functional capacity in patients with pulmonary arterial hypertension.* J Bras Pneumol, 2022. **48**(3): p. e20210483.

18. Nakazato, L., et al., *Association of daily physical activity with psychosocial aspects and functional capacity in patients with pulmonary arterial hypertension: a cross-sectional study.* Pulm Circ, 2021. **11**(2): p. 2045894021999955.

19. Zampogna, E., et al., *The 5-Repetition Sit-to-Stand Test as an Outcome Measure for Pulmonary Rehabilitation in Subjects With Asthma.* Respir Care, 2021. **66**(5): p. 769-776.

20. Yilmaz, A., et al., *Comparison of balance and coordination abilities between asthmatic patients and healthy subjects Balance and coordination abilities in asthmatic patients.* Annals of Clinical and Analytical Medicine, 2021.

21. Atalay, O.T., et al., *Whole-Body Vibration or Aerobic Exercise in Patients with Bronchiectasis? A Randomized Controlled Study.* Medicina (Kaunas, Lithuania), 2022. **58**(12).

22. McKeough, Z., et al., *An observational study of self-reported sedentary behaviour in people with chronic obstructive pulmonary disease and bronchiectasis.* Braz J Phys Ther, 2020. **24**(5): p. 399-406.

23. Koczulla, A.R., et al., *Effects of Vibration Training in Interstitial Lung Diseases: A Randomized Controlled Trial.* Respiration, 2020. **99**(8): p. 658-666.

24. Justice, J.N., et al., *Senolytics in idiopathic pulmonary fibrosis: Results from a first-in-human, open-label, pilot study.* EBioMedicine, 2019. **40**: p. 554-563.

25. Bloem, A.E.M., et al., *Validation of 4-meter-gait-speed test and 5-repetitions-sit-to-stand test in patients with pulmonary fibrosis: a clinimetric validation study.* Sarcoidossis Vascukitis and Diffuse Lung Diseases, 2018. **35**: p. 317-326.

26. Mendes, P., et al., *Skeletal muscle atrophy in advanced interstitial lung disease.* Respirology, 2015. **20**(6): p. 953-9.

27. Okamura, M., et al., *Impact of grip strength and gait speed on exercise tolerance in patients with pulmonary hypertension without left heart disease.* Heart Vessels, 2022. **37**(11): p. 1928-1936.
28. Gonzalez-Saiz, L., et al., *Benefits of skeletal-muscle exercise training in pulmonary arterial hypertension: The WHOLEi+12 trial.* Int J Cardiol, 2017. **231**: p. 277-283.
29. Majewski, M., et al., *Evaluation of a Home-Based Pulmonary Rehabilitation Program for Older Females Suffering from Bronchial Asthma.* Adv Clin Exp Med, 2015. **24**(6): p. 1079-83.
30. Sheppard, E., et al., *Functional Tests of Leg Muscle Strength and Power in Adults With Cystic Fibrosis.* Respir Care, 2019. **64**(1): p. 40-47.
31. Chikina, S.Y., et al., *A comparison of informative between 6-minute walking test and sit-to-stand test in patients with fibrosing interstitial lung diseases.* Pulmonologiya, 2022. **32**(2): p. 208-215.
32. Vainshelboim, B., et al., *8-Foot-Up-and-Go Test is Associated with Hospitalizations and Mortality in Idiopathic Pulmonary Fibrosis: A Prospective Pilot Study.* Lung, 2019. **197**(1): p. 81-88.
33. Vainshelboim, B., et al., *Long-term effects of a 12-week exercise training program on clinical outcomes in idiopathic pulmonary fibrosis.* Lung, 2015. **193**(3): p. 345-54.
34. Vainshelboim, B., et al., *Exercise training-based pulmonary rehabilitation program is clinically beneficial for idiopathic pulmonary fibrosis.* Respiration, 2014. **88**(5): p. 378-88.
35. Kahraman, B., et al., *Effects of neuromuscular electrical stimulation in patients with pulmonary arterial hypertension: a randomized controlled pilot study.* J Cardiol, 2020. **75**(6): p. 702-708.
36. Kahraman, B., et al., *Test-retest reliability and validity of the timed up and go test and 30-second sit to stand test in patients with pulmonary hypertension.* Int J Cardiol, 2020. **304**: p. 159-163.
37. Ozsoy, I., et al., *Gait speed predictors and gait-speed cut-off score to discriminate asthma control status and physical activity in patients with asthma.* Adv Respir Med, 2022. **90**(3): p. 164-170.
38. Martínez-García, M.D.M., J.J. Rodríguez-Juan, and J.D. Ruiz-Cárdenas, *Influence of sex gap on muscle strength and functional mobility in patients with cystic fibrosis.* Appl Physiol Nutr Metab, 2020. **45**(4): p. 387-392.
39. Nolan, C.M., et al., *Change in gait speed and adverse outcomes in patients with idiopathic pulmonary fibrosis: A prospective cohort study.* Respirology, 2023.
40. Hirabayashi, R., et al., *The validity and reliability of four-meter gait speed test for stable interstitial lung disease patients: the prospective study.* J Thorac Dis, 2020. **12**(4): p. 1296-1304.

41. Guler, S.A., et al., *Body composition, muscle function, and physical performance in fibrotic interstitial lung disease: a prospective cohort study.* Respir Res, 2019. **20**(1): p. 56-65.

42. Nolan, C.M., et al., *Phenotypic characteristics associated with slow gait speed in idiopathic pulmonary fibrosis.* Respirology, 2018. **23**(5): p. 498-506.

43. Nolan, C.M., et al., *Gait speed and prognosis in patients with idiopathic pulmonary fibrosis: a prospective cohort study.* Eur Respir J, 2019. **53**(2): p. 1801186.

44. Ryerson, C.J., et al., *Pulmonary rehabilitation improves long-term outcomes in interstitial lung disease: a prospective cohort study.* Respiratory Medicine, 2014. **108**(1): p. 203-210.

45. Babar, H.A., et al., *Determine the Functional Limitations in Activities of Daily Living Through Short Physical Performance Battery Test Among Asthmatic Adults.* Pakistan Journal of Medical and Health Sciences, 2022. **16**(9): p. 470-471.

46. Hanada, M., et al., *A comparative study of the sarcopenia screening in older patients with interstitial lung disease.* BMC Pulm Med, 2022. **22**(1): p. 45.

47. Perez, A.A., et al., *Improvements in frailty contribute to substantial improvements in quality of life after lung transplantation in patients with cystic fibrosis.* Pediatr Pulmonol, 2020. **55**(6): p. 1406-1413.

48. Vardar-Yagli, N., et al., *Gait and functional balance in non-CF bronchiectasis.* Physiother Theory Pract, 2022: p. 1-8.

49. Hena, R., et al., *Cardiorespiratory Responses to Glittre ADL Test in Bronchiectasis: A Cross-Sectional Study.* Can Respir J, 2018. **17**: p. 7470387.

50. Reinaldo, G.P., et al., *Validity and reliability of the Glittre-ADL test in individuals with idiopathic pulmonary fibrosis.* Physiother Theory Pract, 2022: p. 1-9.

51. Alexandre, H.F., et al., *Reliability and validity of the Glittre-ADL test to assess the functional status of patients with interstitial lung disease.* Chron Respir Dis, 2021. **18**: p. 14799731211012962.

52. Olson, A.L., et al., *Physical functional capacity in idiopathic pulmonary fibrosis: performance characteristics of the continuous-scale physical function performance test.* Expert Rev Respir Med, 2015. **9**(3): p. 361-7.

53. Rusanov, V., et al., *Use of the 15-steps climbing exercise oximetry test in patients with idiopathic pulmonary fibrosis.* Respir Med, 2008. **102**(7): p. 1080-8.

54. Olivera, C.M., et al., *Asthma self-management model: randomized controlled trial.* Health Educ Res, 2016. **31**(5): p. 639-52.

55. Newhouse, N., et al., *Randomised feasibility study of a novel experience-based internet intervention to support self-management in chronic asthma.* BMJ Open, 2016. **6**(12): p. e013401.

56. Meyer, A., et al., *A 12-month, moderate-intensity exercise training program improves fitness and quality of life in adults with asthma: a controlled trial.* BMC Pulm Med, 2015. **15**: p. 56-64.

57. Pai, H.J., et al., *A randomized, controlled, crossover study in patients with mild and moderate asthma undergoing treatment with traditional Chinese acupuncture.* Clinics, 2015. **70**(10): p. 663-669.

58. Ochmann, U., et al., *Long-term efficacy of pulmonary rehabilitation in patients with occupational respiratory diseases.* Respiration, 2012. **84**(5): p. 396-405.

59. Smith, A.M., et al., *Asthma in the elderly: risk factors and impact on physical function.* Ann Allergy Asthma Immunol, 2012. **108**(5): p. 305-10.

60. Turner, S., et al., *Improvements in symptoms and quality of life following exercise training in older adults with moderate/severe persistent asthma.* Respiration, 2011. **81**(4): p. 302-10.

61. Kligler, B., et al., *Randomized trial of the effect of an integrative medicine approach to the management of asthma in adults on disease-related quality of life and pulmonary function.* Altern Ther Health Med., 2011. **17**(1): p. 10-5.

62. Siroux, V., et al., *Quality-of-life and asthma-severity in general population asthmatics: results of the ECRHS II study.* Allergy, 2008. **63**(5): p. 547-54.

63. Tohda, Y., et al., *Usefulness of QVAR for the treatment of bronchial asthma--with and without use of an inhalation device.* J Asthma, 2006. **43**(8): p. 613-8.

64. McClish, D.K., et al., *Health related quality of life in sickle cell patients: the PiSCES project.* Health Qual Life Outcomes, 2005. **3**: p. 50-57.

65. De Oliveira, M.A., et al., *Validation of a simplified quality-of-life questionnaire for socioeconomically deprived asthma patients.* J Asthma, 2005. **42**(1): p. 41-4.

66. Matheson, M., et al., *Wheeze not current asthma affects quality of life in young adults with asthma.* Thorax, 2012. **57**: p. 165–167.

67. Stavem, K., et al., *The health-related quality of life of patients with epilepsy compared with angina pectoris, rheumatoid arthritis, asthma and chronic obstructive pulmonary disease.* Qual lLife Res, 2000. **9**(7): p. 865-71.

68. Dyer, C.A., et al., *Quality of life in elderly subjects with a diagnostic label of asthma from general practice registers.* Eur Respir J, 1999. **14**(1): p. 39-45.

69. Ware, J., et al., *The Responsiveness of Disease-Specific and Generic Health Measures to Changes in the Severity of Asthma among Adults.* Qual Life Res, 1998. **7**: p. 235-244.

70. Blanc, P., et al., *Pulmonary and allergy subspecialty care in adults with asthma: Treatment, use of services, and health outcomes.* Pulmonary and Allergy Asthma, 1997. **167**(6): p. 398-407.

71. Van der Molen, T., et al., *Discriminative aspects of two generic and two asthma-specific instruments: relation with symptoms, bronchodilator use and lung function in patients with mild asthma.* Quality of Life Research, 1997. **6**: p. 353–361.

72. Okamoto, L.J., et al., *Fluticasone propionate improves quality of life in patients with asthma requiring oral corticosteroids.* Ann Allergy Asthma Immunol 1996. **76**: p. 455–61.

73. Bulcun, E., et al., *Quality of Life and Bronchial Hyper-Responsiveness in Subjects With Bronchiectasis: Validation of the Seattle Obstructive Lung Disease Questionnaire in Bronchiectasis.* Respir Care, 2015. **60**(11): p. 1616-23.

74. Jacques, P.S., et al., *Distância percorrida no teste de caminhada de seis minutos não se relaciona com qualidade de vida em pacientes com bronquiectasias não fibrocísticas.* J Bras Pneumol. , 2012. **38**(2): p. 346-355.

75. Lee, A.L., et al., *Clinical determinants of the 6-Minute Walk Test in bronchiectasis.* Respir Med, 2009. **103**(5): p. 780-5.

76. Guilemany, J.M., et al., *The impact of bronchiectasis associated to sinonasal disease on quality of life.* Respir Med, 2006. **100**(11): p. 1997-2003.

77. Gee, L., et al., *Development of a disease specific health related quality of life measure for adults and adolescents with cystic fibrosis.* Thorax, 2000. **55**: p. 946-954.

78. Sikora, M., et al., *Impact of physical functional capacity on quality of life in patients with interstitial lung diseases.* Respir Physiol Neurobiol, 2023. **313**: p. 104064.

79. Aboelmagd M, F. and S. Moawd, *Efficacy of inspiratory muscle training on inspiratory muscles strength, functional capacity, and quality of life in patients with interstitial lung disease. A single non-controlled clinical study.* Physiotherapy Quarterly, 2022. **30**(2): p. 46-50.

80. Machado, F.V.C., et al., *Relationship between body composition, exercise capacity and health-related quality of life in idiopathic pulmonary fibrosis.* BMJ Open Respir Res, 2021. **8**(1).

81. Aguiar, W.F., et al., *Translation, cross-cultural adaptation, and measurement properties of the Brazilian-Portuguese version of the idiopathic pulmonary fibrosis-specific version of the Saint George's Respiratory Questionnaire (SGRQ-I) for patients with interstitial lung disease.* Braz J Phys Ther, 2021. **25**(6): p. 794-802.
82. Zhao, R., et al., *Associated factors with interstitial lung disease and health-related quality of life in Chinese patients with primary Sjogren's syndrome.* Clin Rheumatol, 2020. **39**(2): p. 483-489.
83. Dalichau, S. and T. Moller, *[Sustainability in Outpatient Pulmonary Rehabilitation in Patients with Asbestosis - Results of an 8-Year Follow Up].* Pneumologie, 2020. **74**(4): p. 201-209.
84. Vis, R., et al., *Randomised, placebo-controlled trial of dexamethasone for quality of life in pulmonary sarcoidosis.* Respir Med, 2020. **165**: p. 105936.
85. Witt, S., et al., *Psychometric properties and minimal important differences of SF-36 in Idiopathic Pulmonary Fibrosis.* Respir Res, 2019. **20**(1): p. 47-58.
86. Yalniz, E., et al., *Are idiopathic pulmonary fibrosis patients more anxious and depressive than patient's with other interstitial lung disease?* Sarcoidosis Vasc Diffuse Lung Dis, 2019. **36**(4): p. 294-301.
87. Pilzak, K., et al., *Physical Functioning and Symptoms of Chronic Fatigue in Sarcoidosis Patients.* Adv Exp Med Biol, 2018. **1040**: p. 13-21.
88. Naz, I., et al., *Efficacy of a Structured Exercise Program for Improving Functional Capacity and Quality of Life in Patients With Stage 3 and 4 Sarcoidosis: A randomized controlled trial.* J Cardiopulm Rehabil Prev, 2018. **38**(2): p. 124-130.
89. Fisher, C.J., et al., *Reliability, construct validity and responsiveness to change of the PROMIS-29 in systemic sclerosis-associated interstitial lung disease.* Clin Exp Rheumatol, 2019. **Suppl 119**(4): p. 49-56.
90. Tomioka, H., et al., *Combined pulmonary fibrosis and emphysema: effect of pulmonary rehabilitation in comparison with chronic obstructive pulmonary disease.* BMJ Open Respir Res, 2016. **3**(1): p. e000099.
91. Dalichau, S., et al., *[Short- and long-term effects of the outpatient medical rehabilitation for patients with asbestosis].* Pneumologie, 2010. **64**(3): p. 163-70.
92. Hinchcliff, M.E., et al., *Longitudinal evaluation of PROMIS-29 and FACIT-dyspnea short forms in systemic sclerosis.* J Rheumatol, 2015. **42**(1): p. 64-72.

93. Alhamad, E.H., *Pirfenidone treatment in idiopathic pulmonary fibrosis: A Saudi experience.* Ann Thorac Med, 2015. **10**(1): p. 38-43.

94. du Bois, R.M., et al., *Forced vital capacity in patients with idiopathic pulmonary fibrosis: test properties and minimal clinically important difference.* Am J Respir Crit Care Med, 2011. **184**(12): p. 1382-9.

95. Lumetti, F., et al., *Quality of life and functional disability in patients with interstitial lung disease related to Systemic Sclerosis.* Acta Biomed, 2015. **86**(2): p. 142-148.

96. Theodore, A.C., et al., *Correlation of cough with disease activity and treatment with cyclophosphamide in scleroderma interstitial lung disease: findings from the Scleroderma Lung Study.* Chest, 2012. **142**(3): p. 614-621.

97. Swigris, J.J., et al., *The UCSD shortness of breath questionnaire has longitudinal construct validity in idiopathic pulmonary fibrosis.* Respir Med, 2012. **106**(10): p. 1447-55.

98. Swigris, J.J., et al., *Benefits of pulmonary rehabilitation in idiopathic pulmonary fibrosis.* Respir Care, 2011. **56**(6): p. 783-9.

99. Krishnan, V., et al., *Sleep quality and health-related quality of life in idiopathic pulmonary fibrosis.* Chest, 2008. **134**(4): p. 693-698.

100. Zimmermann, C., et al., *Comparison of two questionnaires which measure the health-related quality of life of idiopathic pulmonary fibrosis patients.* Braz J Med Biol Res, 2007. **40**(2): p. 179-187.

101. Ohno, S., et al., *Reassessment of the Classification of the Severity in Idiopathic Pulmonary Fibrosis Using SF-36 Questionnaire.* Internal Medicine, 2005. **44**: p. 196-199.

102. Chang, J.A., et al., *Assessment of health-related quality of life in patients with interstitial lung disease.* Chest, 1999. **116**(5): p. 1175-82.

103. Masa, J.F., et al., *Risk factors associated with pulmonary hypertension in obesity hypoventilation syndrome.* J Clin Sleep Med, 2022. **18**(4): p. 983-992.

104. Karapolat, H., et al., *Effects of cardiopulmonary rehabilitation on pulmonary arterial hypertension: A prospective, randomized study.* Turk J Phys Med Rehabil, 2019. **65**(3): p. 278-286.

105. Babu, A.S., et al., *Effects of home-based exercise training on functional outcomes and quality of life in patients with pulmonary hypertension: A randomized clinical trial.* Indian Heart J, 2019. **71**(2): p. 161-165.

106. Kukkonen, M., A. Puhakka, and M. Halme, *Quality of life among pulmonary hypertension patients in Finland.* Eur Clin Respir J, 2016. **3**: p. 26405.

107. Mathai, S.C., et al., *Sex differences in response to tadalafil in pulmonary arterial hypertension.* Chest, 2015. **147**(1): p. 188-197.

108. Laoutaris, I.D., et al., *Benefits of inspiratory muscle training in patients with pulmonary hypertension: A pilot study.* Hellenic J Cardiol, 2016. **20**(16): p. 30155-5.

109. Matura, L.A., A. McDonough, and D.L. Carroll, *Health-related quality of life and psychological states in patients with pulmonary arterial hypertension.* J Cardiovasc Nurs, 2014. **29**(2): p. 178-84.

110. Matura, L.A., A. McDonough, and D.L. Carroll, *Cluster analysis of symptoms in pulmonary arterial hypertension: a pilot study.* Eur J Cardiovasc Nurs, 2012. **11**(1): p. 51-61.

111. Grunig, E., et al., *Safety and efficacy of exercise training in various forms of pulmonary hypertension.* Eur Respir J, 2012. **40**(1): p. 84-92.

112. Gilbert, C., et al., *Estimating a minimally important difference in pulmonary arterial hypertension following treatment with sildenafil.* Chest, 2009. **135**(1): p. 137-142.

113. Pepke-Zaba, J., et al., *Tadalafil therapy and health-related quality of life in pulmonary arterial hypertension.* Curr Med Res Opin, 2009. **25**(10): p. 2479-85.

114. Galie, N., et al., *Ambrisentan for the treatment of pulmonary arterial hypertension: results of the ambrisentan in pulmonary arterial hypertension, randomized, double-blind, placebo-controlled, multicenter, efficacy (ARIES) study 1 and 2.* Circulation, 2008. **117**(23): p. 3010-9.

115. Souza, R., et al., *Effect of sitaxsentan treatment on quality of life in pulmonary arterial hypertension.* Int J Clin Pract, 2007. **61**(1): p. 153-6.

116. Mereles, D., et al., *Exercise and respiratory training improve exercise capacity and quality of life in patients with severe chronic pulmonary hypertension.* Circulation, 2006. **114**(14): p. 1482-9.

117. White, J., et al., *Cognitive, emotional, and quality of life outcomes in patients with pulmonary arterial hypertension.* Respir Res, 2006. **7**: p. 55-10.

118. Souza, R., et al., *Effect of bosentan treatment on surrogate markers in pulmonary arterial hypertension.* Curr Med Res Opin, 2005. **21**(6): p. 907-11.

119. Miozzo, A.P., et al., *Ambulatory oxygen therapy in lung transplantation candidates with idiopathic pulmonary fibrosis referred for pulmonary rehabilitation.* J Bras Pneumol, 2023. **49**(2): p. e20220280.

120. Langer, D., et al., *Determinants of physical activity in daily life in candidates for lung transplantation.* Respir Med, 2012. **106**(5): p. 747-54.

121. Feltrim, M.I., et al., *The quality of life of patients on the lung transplantation waiting list.* Transplant Proc, 2008. **40**(3): p. 819-21.

122. Duruturk, N., M. Acar, and M.I. Dogrul, *Effect of Inspiratory Muscle Training in the Management of Patients With Asthma: A Randomized controlled trial.* J Cardiopulm Rehabil Prev, 2018. **38**(3): p. 198-203.

123. Zadeh, M., et al., *Effects of exercise with lower and upper extremities on respiratory and exercise capacities of asthmatic patients.* Koomesh, 2013. **15**(1): p. 89-101.

124. de Camargo, C.O., et al., *Quality of Life Questionnaire-Bronchiectasis: a study of the psychometric properties of the Brazilian Portuguese version.* Clin Rehabil, 2020. **34**(7): p. 960-970.

125. Lavery, K.A., et al., *Expert patient self-management program versus usual care in bronchiectasis: a randomized controlled trial.* Arch Phys Med Rehabil, 2011. **92**(8): p. 1194-201.

126. Martinez-Garcia, M.A., et al., *Inhaled steroids improve quality of life in patients with steady-state bronchiectasis.* Respir Med, 2006. **100**(9): p. 1623-32.

127. Wilson, C., et al., *Validation of the St. George's Respiratory Questionnaire in Bronchiectasis.* Am J Respir Crit Care Med, 1997. **156**: p. 536-541.

128. Zaki, S., et al., *Does inspiratory muscle training provide additional benefits during pulmonary rehabilitation in people with interstitial lung disease? A randomized control trial.* Physiother Theory Pract, 2023. **39**(3): p. 518-528.

129. Essam, H., et al., *Effects of different exercise training programs on the functional performance in fibrosing interstitial lung diseases: A randomized trial.* PLoS One, 2022. **17**(5): p. e0268589.

130. Fujita, K., et al., *Frequency and impact on clinical outcomes of sarcopenia in patients with idiopathic pulmonary fibrosis.* Chron Respir Dis, 2022. **19**: p. 14799731221117298.

131. Ebihara, K., et al., *Appendicular Skeletal Muscle Mass Correlates with Patient-Reported Outcomes and Physical Performance in Patients with Idiopathic Pulmonary Fibrosis.* Tohoku J Exp Med, 2021. **253**(1): p. 61-68.

132. Janssen, K., et al., *The impact of palliative care on quality of life, anxiety, and depression in idiopathic pulmonary fibrosis: a randomized controlled pilot study.* Respir Res, 2020. **21**(1): p. 2-11.

133. Creamer, A.W. and S.L. Barratt, *Does ambulatory oxygen improve quality of life in patients with fibrotic lung disease? Results from the AmbOx trial.* Breathe (Sheff), 2019. **15**(2): p. 140-143.
134. Santana, P.V., et al., *Diaphragmatic ultrasound findings correlate with dyspnea, exercise tolerance, health-related quality of life and lung function in patients with fibrotic interstitial lung disease.* BMC Pulm Med, 2019. **19**(1): p. 183.
135. Dowman, L.M., et al., *The evidence of benefits of exercise training in interstitial lung disease: a randomised controlled trial.* Thorax, 2017. **72**: p. 610–619.
136. Braz, N.F., et al., *Influence of Cytokines and Soluble Receptors in the Quality of Life and Functional Capacity of Workers Exposed to Silica.* J Occup Environ Med, 2016. **58**(3): p. 272-6.
137. Karadalli, M.N., et al., *Effects of Inspiratory Muscle Training in Subjects With Sarcoidosis: A Randomized Controlled Clinical Trial.* Respir Care, 2016. **61**(4): p. 483-94.
138. Drake, W.P., et al., *Effects of broad-spectrum antimycobacterial therapy on chronic pulmonary sarcoidosis.* Sarcoidosis Vasc Diffuse Lung Dis, 2014. **30**(3): p. 201-211.
139. Sozener, Z., G. Karabiyikoglu, and N. Duzgun, *Evaluation of the functional parameters in scleroderma cases with pulmonary involvement.* Tüberküloz ve Toraks Dergisi 2010. **58**(3): p. 235-241.
140. Alotaibi, M., et al., *Metabolomic Profiles Differentiate Scleroderma-PAH From Idiopathic PAH and Correspond With Worsened Functional Capacity.* Chest, 2023. **163**(1): p. 204-215.
141. Matura, L.A., et al., *Symptom phenotypes in pulmonary arterial hypertension: The PAH "symptome".* Pulm Circ, 2022. **12**(3): p. e12135.
142. Arvanitaki, A., et al., *Quality of Life is Related to Haemodynamics in Precapillary Pulmonary Hypertension.* Heart Lung Circ, 2020. **29**(1): p. 142-148.
143. Karauzum, K., et al., *Bendopnea and Its Clinical Importance in Outpatient Patients with Pulmonary Arterial Hypertension.* Acta Cardiol Sin, 2018. **34**(6): p. 518-525.
144. Aldemir, M., et al., *Primary pulmonary arterial hypertension with preserved right ventricular function leads to lower extremity venous insufficiency.* Vascular, 2018. **26**(2): p. 183-188.
145. Mihai, C., et al., *Factors associated with disease progression in early-diagnosed pulmonary arterial hypertension associated with systemic sclerosis: longitudinal data from the DETECT cohort.* Ann Rheum Dis, 2018. **77**: p. 128-132.

146. Waligora, M., et al., *Mechanism and prognostic role of qR in V1 in patients with pulmonary arterial hypertension.* J Electrocardiol, 2017. **50**(4): p. 476-483.
147. Segrera, S.A., et al., *Open label study of ambrisentan in patients with exercise pulmonary hypertension.* Pulm Circ, 2017. **7**(2): p. 531-538.
148. Tanabe, N., et al., *Efficacy and Safety of an Orally Administered Selective Prostacyclin Receptor Agonist, Selexipag, in Japanese Patients With Pulmonary Arterial Hypertension.* Circ J, 2017. **81**(9): p. 1360-1367.
149. Saha, S.K., S. Soderberg, and P. Lindqvist, *Association of Right Atrial Mechanics with Hemodynamics and Physical Capacity in Patients with Idiopathic Pulmonary Arterial Hypertension: Insight from a Single-Center Cohort in Northern Sweden.* Echocardiography, 2016. **33**(1): p. 46-56.
150. Godinas, L., et al., *Lung capillary blood volume and membrane diffusion in precapillary pulmonary hypertension.* J Heart Lung Transplant, 2016. **35**(5): p. 647-56.
151. Rubin, L.J., et al., *Riociguat for the treatment of pulmonary arterial hypertension: a long-term extension study (PATENT-2).* Eur Respir J, 2015. **45**(5): p. 1211-3.
152. Frost, A.E., et al., *Long-term safety and efficacy of imatinib in pulmonary arterial hypertension.* J Heart Lung Transplant, 2015. **34**(11): p. 1366-75.
153. Webb, D.J., et al., *Sildenafil improves renal function in patients with pulmonary arterial hypertension.* Br J Clin Pharmacol, 2015. **80**(2): p. 235-41.
154. Zhuang, Y., et al., *Randomized study of adding tadalafil to existing ambrisentan in pulmonary arterial hypertension.* Hypertens Res, 2014. **37**(6): p. 507-12.
155. Mouratoglou, S.A., et al., *Duration of interventricular septal shift toward the left ventricle is associated with poor clinical outcome in precapillary pulmonary hypertension: A cardiac magnetic resonance study.* Hellenic J Cardiol, 2014. **61**(2): p. 112-117.
156. Ghofrani, H.A., et al., *Riociguat for the treatment of pulmonary arterial hypertension.* N Engl J Med, 2013. **369**(4): p. 330-40.
157. Oudiz, R.J., et al., *Tadalafil for the treatment of pulmonary arterial hypertension: a double-blind 52-week uncontrolled extension study.* J Am Coll Cardiol, 2012. **60**(8): p. 768-74.
158. Condliffe, R., et al., *Connective tissue disease-associated pulmonary arterial hypertension in the modern treatment era.* Am J Respir Crit Care Med, 2009. **179**(2): p. 151-7.

159. Galie, N., et al., *Tadalafil therapy for pulmonary arterial hypertension.* Circulation, 2009. **119**(22): p. 2894-903.

160. Galie, N., et al., *Ambrisentan therapy for pulmonary arterial hypertension.* J Am Coll Cardiol, 2005. **46**(3): p. 529-35.

161. Galiè, N., et al., *Sildenafil citrate therapy for pulmonary arterial hypertension.* N Engl J Med, 2005. **356**: p. 2148-57.

162. Kaptan Ozen, D., et al., *The effect of global longitudinal strain on impaired six-minute walk test performance in patients with sarcoidosis.* Sarcoidosis Vasc Diffuse Lung Dis, 2020. **37**(1): p. 66-73.

163. Unlu, S., et al., *Right ventricular strain related to pulmonary artery pressure predicts clinical outcome in patients with pulmonary arterial hypertension.* Eur Heart J Cardiovasc Imaging, 2023. **24**(5): p. 635-642.

164. Bunclark, K., et al., *A minimal clinically important difference measured by the Cambridge Pulmonary Hypertension Outcome Review for patients with idiopathic pulmonary arterial hypertension.* Pulm Circ, 2021. **11**(2): p. 2045894021995055.

165. Karelkina EV, G.N., Simakova MA, Moiseeva OM, *[Experience with Selexipag to Treat Pulmonary Arterial Hypertension].* Kardiologiia, 2020. **60**(4): p. 36-42.

166. Naal, T., et al., *Serum Chloride Levels Track With Survival in Patients With Pulmonary Arterial Hypertension.* Chest, 2018. **154**(3): p. 541-549.

167. Ozpelit, E., et al., *Prognostic value of neutrophil-to-lymphocyte ratio in pulmonary arterial hypertension.* J Int Med Res, 2015. **43**(5): p. 661-71.

168. Chueamuangphan, N., et al., *Benefits of chronic blood transfusion in hemoglobin E/beta thalassemia with pulmonary arterial hypertension.* Int J Gen Med, 2014. **7**: p. 411-6.

169. Malik, N., K. McCarthy, and O.A. Minai, *Prevalence and significance of decreased bone density in pulmonary arterial hypertension.* South Med J, 2012. **105**(7): p. 344-9.

170. Benza, R.L., et al., *Long-term effects of inhaled treprostinil in patients with pulmonary arterial hypertension: the Treprostinil Sodium Inhalation Used in the Management of Pulmonary Arterial Hypertension (TRIUMPH) study open-label extension.* J Heart Lung Transplant, 2011. **30**(12): p. 1327-33.

171. Tokgozoglu, L., et al., *Two years of multidisciplinary diagnostic and therapeutic experience in patients with pulmonary arterial hypertension.* Arch Turk Soc Cardiol 2009. **37**(6): p. 378-383.

172. Minai, O.A., et al., *Predictors of nocturnal oxygen desaturation in pulmonary arterial hypertension.* Chest, 2007. **131**(1): p. 109-17.

173. Chau, E.M., K.Y. Fan, and W.H. Chow, *Effects of chronic sildenafil in patients with Eisenmenger syndrome versus idiopathic pulmonary arterial hypertension.* Int J Cardiol, 2007. **120**(3): p. 301-5.

174. Zafrir, N., et al., *Use of noninvasive tools in primary pulmonary hypertension to assess the correlation of right ventricular function with functional capacity and to predict outcome.* Int J Cardiovasc Imaging, 2007. **23**(2): p. 209-15.

175. Souza, R., et al., *NT-proBNP as a tool to stratify disease severity in pulmonary arterial hypertension.* Respir Med, 2007. **101**(1): p. 69-75.

176. McLaughlin, V.V., et al., *Randomized study of adding inhaled iloprost to existing bosentan in pulmonary arterial hypertension.* Am J Respir Crit Care Med, 2006. **174**(11): p. 1257-63.

177. Cenedese, E., et al., *Measurement of quality of life in pulmonary hypertension and its significance.* Eur Respir J, 2006. **28**(4): p. 808-15.

178. Shen, J., B. He, and B. Wang, *Effects of lipo-prostaglandin E1 on pulmonary hemodynamics and clinical outcomes in patients with pulmonary arterial hypertension.* Chest, 2005. **128**(2): p. 714-9.

179. Badesch, D.B., et al., *Continuous intravenous epoprostenol for pulmonary hypertension due to the scleroderma spectrum of disease.* Ann Intern Med, 2000. **132**(6): p. 425-434.

180. Yigla, M., et al., *Unexplained severe pulmonary hypertension in the elderly: report on 14 patients.* Isr Med Assoc J, 1997. **6**(2): p. 78-81.

181. Calik-Kutukcu, E., et al., *Validity and reliability of 6-minute pegboard and ring test in patients with asthma.* J Asthma, 2022. **59**(7): p. 1387-1395.

182. Freeman, A., et al., *Exercise Training Induces a Shift in Extracellular Redox Status with Alterations in the Pulmonary and Systemic Redox Landscape in Asthma.* Antioxidants (Basel), 2021. **10**(12): p. 1926.

183. Lage, S.M., et al., *Efficacy of inspiratory muscle training on inspiratory muscle function, functional capacity, and quality of life in patients with asthma: A randomized controlled trial.* Clin Rehabil, 2021. **35**(6): p. 870-881.

184. Evaristo, K.B., et al., *Effects of Aerobic Training Versus Breathing Exercises on Asthma Control: A Randomized Trial.* J Allergy Clin Immunol Pract, 2020. **8**(9): p. 2989-2996 e4.

185. Majd, S., et al., *A Feasibility Study of a Randomized Controlled Trial of Asthma-Tailored Pulmonary Rehabilitation Compared with Usual Care in Adults with Severe Asthma.* J Allergy Clin Immunol Pract, 2020. **8**(10): p. 3418-3427.

186. Freitas, P.D., et al., *The Role of Exercise in a Weight-Loss Program on Clinical Control in Obese Adults with Asthma. A Randomized Controlled Trial.* Am J Respir Crit Care Med, 2017. **195**(1): p. 32-42.

187. Refaat, A. and M. Gawish, *Effect of physical training on health-related quality of life in patients with moderate and severe asthma.* Egyptian Journal of Chest Diseases and Tuberculosis, 2015. **64**(4): p. 761-766.

188. Pakhale, S., et al., *Effects of weight loss on airway responsiveness in obese adults with asthma: does weight loss lead to reversibility of asthma?* Chest, 2015. **147**(6): p. 1582-1590.

189. Rondinel, T.Z., et al., *Incentive spirometry combined with expiratory positive airway pressure improves asthma control and quality of life in asthma: a randomised controlled trial.* J Asthma, 2015. **52**(2): p. 220-6.

190. Vempati, R., R.L. Bijlani, and K.K. Deepak, *The efficacy of a comprehensive lifestyle modification programme based on yoga in the management of bronchial asthma: a randomized controlled trial.* BMC Pulm Med, 2009. **9**: p. 37.

191. Choi, J.-S., et al., *Effect of high dose inhaled glucocorticoids on quality of life in patients with moderate to severe asthma.* J Korean Med Sci, 2005. **20**: p. 586-90.

192. Riccioni, G., et al., *Efectiveness of montelukast versus budesonide on quality of life and bronchial reactivity in subjects with mild-persistent asthma.* International Journal of Immunopathology and Pharmacology, 2002. **15**(2): p. 149-155.

193. Busse, W., et al., *Efficacy, safety, and impact on quality of life of salmeterol in patients with moderate persistent asthma.* Am J Manag Care, 1988. **4**(11): p. 1579-87.

194. van der Molen, T., et al., *Quality of life during formoterol treatment: comparison between asthma-specific and generic questionnaires. Canadian and the Dutch Formoterol Investigators.* Eur Respir J, 1998. **12**(1): p. 30-4.

195. Juniper, E., et al., *Determining a minimal important change in a disease-specific quality of life questionnaire.* J Clin Epidemiol, 1994. **47**(1): p. 81-87.
196. Juniper, E., et al., *Measuring quality of life in asthma.* Am Rev Respir Dis, 1993. **147**: p. 832-838.
197. Knudsen, K.B., et al., *Coach to cope: feasibility of a life coaching program for young adults with cystic fibrosis.* Patient Prefer Adherence, 2017. **11**: p. 1613-1623.
198. Aguiar, K.C.A., et al., *Physical performance, quality of life and sexual satisfaction evaluation in adults with cystic fibrosis: An unexplored correlation.* Rev Port Pneumol (2006), 2017. **23**(4): p. 179-192.
199. Ribeiro Moco, V.J., et al., *Pulmonary function, functional capacity and quality of life in adults with cystic fibrosis.* Rev Port Pneumol (2006), 2015. **21**(4): p. 198-202.
200. Penafortes, J.T., et al., *Association among posture, lung function and functional capacity in cystic fibrosis.* Rev Port Pneumol, 2013. **19**(1): p. 1-6.
201. Dill, E.J., et al., *Longitudinal trends in health-related quality of life in adults with cystic fibrosis.* Chest, 2013. **144**(3): p. 981-989.
202. Kelemen, L., et al., *Pain impacts on quality of life and interferes with treatment in adults with cystic fibrosis.* Physiother Res Int, 2012. **17**(3): p. 132-41.
203. Sandsund, C.A., et al., *Musculoskeletal techniques for clinically stable adults with cystic fibrosis: a preliminary randomised controlled trial.* Physiotherapy, 2011. **97**(3): p. 209-17.
204. Young, A.C., et al., *Randomised placebo controlled trial of non-invasive ventilation for hypercapnia in cystic fibrosis.* Thorax, 2008. **63**(1): p. 72-7.
205. Liu, W.T., et al., *A mobile telephone-based interactive self-care system improves asthma control.* Eur Respir J, 2011. **37**(2): p. 310-7.
206. Yount, S.E., et al., *Health-Related Quality of Life in Patients with Idiopathic Pulmonary Fibrosis.* Lung, 2016. **194**(2): p. 227-34.
207. Mendes, F.A., et al., *Association between maximal aerobic capacity and psychosocial factors in adults with moderate-to-severe asthma.* J Asthma, 2013. **50**(6): p. 595-9.
208. Koudstaal, T., et al., *The Effects of a 10-wk Outpatient Pulmonary Rehabilitation Program on Exercise Performance, Muscle Strength, Soluble Biomarkers, and Quality of Life in Patients With Pulmonary Hypertension.* J Cardiopulm Rehabil Prev, 2019. **39**(6): p. 397-402.

209. Lee, A.L., et al., *The clinical impact of self-reported symptoms of chronic rhinosinusitis in people with bronchiectasis.* Immun Inflamm Dis, 2022. **10**(1): p. 101-110.

210. Jose, A., et al., *Home-based pulmonary rehabilitation in people with bronchiectasis: a randomised controlled trial.* ERJ Open Res, 2021. **7**(2).

211. Georga, G., et al., *The effect of stress management incorporating progressive muscle relaxation and biofeedback-assisted relaxation breathing on patients with asthma: a randomised controlled trial.* Advances in Integrative Medicine, 2019. **6**(2): p. 73-77.

212. Ma, J., et al., *Pilot randomised trial of a healthy eating behavioural intervention in uncontrolled asthma.* Eur Respir J, 2016. **47**(1): p. 122-32.

213. Aslan, G.K., et al., *A randomized controlled trial on inspiratory muscle training in pulmonary hypertension: Effects on respiratory functions, functional exercise capacity, physical activity, and quality of life.* Heart Lung, 2020. **49**(4): p. 381-387.

214. McCormack, F.X., et al., *Efficacy and safety of sirolimus in lymphangioleiomyomatosis.* N Engl J Med, 2011. **364**(17): p. 1595-606.

215. Swigris, J.J., et al., *Assessing dyspnea and its impact on patients with connective tissue disease-related interstitial lung disease.* Respir Med, 2010. **104**(9): p. 1350-5.

216. Mena-Vazquez, N., et al., *Analysis of comorbidity in rheumatoid arthritis-associated interstitial lung disease: a nested case-cohort study.* Biomed Pharmacother, 2023. **157**: p. 114049.

217. Ricotti, S., et al., *Changes in quality of life and functional capacity after lung transplantation: A single-center experience.* Monaldi Arch Chest Dis, 2017. **87**(3): p. 123-129.

218. Nelsen, L.M., et al., *Evaluation of the psychometric properties of the St George's Respiratory Questionnaire in patients with severe asthma.* Respir Med, 2017. **128**: p. 42-49.
